# Germline-Dependent Antibody Paratope States and Pairing Specific V_H_-V_L_ Interface Dynamics

**DOI:** 10.3389/fimmu.2021.675655

**Published:** 2021-08-10

**Authors:** Monica L. Fernández-Quintero, Katharina B. Kroell, Lisa M. Bacher, Johannes R. Loeffler, Patrick K. Quoika, Guy Georges, Alexander Bujotzek, Hubert Kettenberger, Klaus R. Liedl

**Affiliations:** ^1^Department of General, Inorganic and Theoretical Chemistry, and Center for Molecular Biosciences Innsbruck (CMBI), University of Innsbruck, Innsbruck, Austria; ^2^Roche Pharma Research and Early Development, Large Molecule Research, Roche Innovation Center Munich, Penzberg, Germany

**Keywords:** antibodies, germlines, V_H_-V_L_ pairings, paratope states in solution, backbone *vs*. sidechain flexibility

## Abstract

Antibodies have emerged as one of the fastest growing classes of biotherapeutic proteins. To improve the rational design of antibodies, we investigate the conformational diversity of 16 different germline combinations, which are composed of 4 different kappa light chains paired with 4 different heavy chains. In this study, we systematically show that different heavy and light chain pairings strongly influence the paratope, interdomain interaction patterns and the relative V_H_-V_L_ interface orientations. We observe changes in conformational diversity and substantial population shifts of the complementarity determining region (CDR) loops, resulting in distinct dominant solution structures and differently favored canonical structures. Additionally, we identify conformational changes in the structural diversity of the CDR-H3 loop upon different heavy and light chain pairings, as well as upon changes in sequence and structure of the neighboring CDR loops, despite having an identical CDR-H3 loop amino acid sequence. These results can also be transferred to all CDR loops and to the relative V_H_-V_L_ orientation, as certain paratope states favor distinct interface angle distributions. Furthermore, we directly compare the timescales of sidechain rearrangements with the well-described transition kinetics of conformational changes in the backbone of the CDR loops. We show that sidechain flexibilities are strongly affected by distinct heavy and light chain pairings and decipher germline-specific structural features co-determining stability. These findings reveal that all CDR loops are strongly correlated and that distinct heavy and light chain pairings can result in different paratope states in solution, defined by a characteristic combination of CDR loop conformations and V_H_-V_L_ interface orientations. Thus, these results have broad implications in the field of antibody engineering, as they clearly show the importance of considering paired heavy and light chains to understand the antibody binding site, which is one of the key aspects in the design of therapeutics.

## Introduction

Antibodies are a crucial component of the adaptive immune system and are now a major class of biopharmaceuticals (1). The high diversity in the antibody repertoire facilitates the recognition of a wide variety of different antigens. Understanding the antibody-antigen binding interface has become a key factor for advancing the use of antibodies as biotherapeutics, and accordingly the importance of characterizing and engineering the structure of antibodies to optimize affinity, specificity, and certain biophysical properties has increased substantially in the past decades (2).

An antibody usually consists of two heavy and two light chains connected *via* disulfide bonds. In mammals exist five heavy chain isotypes (IgM, IgD, IgG, IgA and IgE) and two light chains isotypes kappa (κ) and lambda (λ), which can result in distinct physicochemical and structural properties (3, 4).

The antigen binding fragment (Fab) consists of a heavy and a light chain and can be divided into a constant (C_H_1 and C_L_) and a variable domain Fv (V_H_ and V_L_). These two domains have a common folding pattern, often referred to as immunoglobulin fold, which is formed by the packing of two anti-parallel β-sheets (2, 5). The antigen-binding site, the paratope, is shaped by a pairing of the V_H_ and V_L_ domains (6). The paratope is composed of up to six hypervariable loops, also known as the complementarity determining regions (CDRs), which contribute to the diversity in sequence and structure of the antibody repertoire (7, 8). In this study, for comparability of all antibodies, the term paratope is defined by all six CDR loops.

The high diversity in length, sequence and structure of the CDR loops presents a challenge to antibody engineering. Five of these six CDR loops have been classified into so-called canonical clusters, assuming that they can only adopt a limited number of main-chain conformations (7, 9–11). No canonical cluster can be assigned to the CDR-H3 loop, because of its huge diversity (12–17). Thus, structure prediction remains challenging. However, to functionally characterize and capture the high flexibility not only of the CDR-H3 loop but of all CDR loops, they are more adequately described as conformational ensembles in solution. Apart from sampling the majority of canonical cluster structures within these ensembles, also additional dominant solution structures have been identified, which are not apparent from X-ray structures, mostly due to crystal packing effects (18, 19). Another crucial aspect co-determining the shape of the antigen binding site is the relative V_H_-V_L_ interdomain orientation. The V_H_-V_L_ interface significantly contributes to the stability of the Fv and has been shown to affect antigen binding kinetics (6, 20, 21). Pairing of the heavy and light chains is an additional way of generating antibody diversity. Still, little is known about the unique mechanism governing V_H_-V_L_ pairing. Various studies tried to elucidate pairing preferences of certain V_H_ and V_L_ gene families and concluded that the heavy and light chain pairings occur randomly (22, 23). However, the importance of understanding the pairing preferences of a particular V_H_ with various distinct light chain sequences, as well as the respective consequences on the antigen binding site, specificity and stability should not be underestimated (24). Thus, together with the CDR loops, the V_H_-V_L_ interface determines the shape and diversity of the paratope. Already a small number of mutations in the framework regions, in particular in the V_H_-V_L_ interface, can result in structural changes of the binding site, which consequently influences antigen recognition and can lead to allosteric conformational rearrangements in the constant domains and the elbow angle (25–30). The majority of Fab interface dynamics have been reported to occur in the low nanosecond timescale, while slower components of the movements are dominated by conformational rearrangements in the CDR loops in the micro-to-millisecond timescales (18, 31, 32). Based on these observations, antibodies were previously described as ensembles of paratope states in solution, which are characterized by a combination of correlated CDR loop conformations and interdomain orientations, which interconvert into each other by synchronous loop and interdomain rearrangements (33).

In this study, we use molecular dynamic simulations to systematically characterize consequences of different heavy and light chain pairings on the antibody paratope in atomistic detail and quantify backbone and side-chain flexibilities.

## Methods

### Dataset

As starting structures for our simulations, we used 16 germline Fab crystal structures from the same library. We chose this dataset as it allows to systematically investigate the influence of different heavy and light chain pairings. The phage library is composed of 4 heavy chain germlines IGHV1-69 (H1-69), IGHV3-23 (H3-23), IGHV5-51 (H5-51) and IGHV3-53 (H5-53) and 4 light chain germlines (all κ) IGKV1-39 (L1-39), IGKV3-11 (L3-11), IGKV3-20 (L3-20) and IGKV4-1 (L4-1). These genes were selected based on the frequency of their use, their cognate canonical structures, which can recognize proteins and peptides and their ability to be expressed in bacteria. Another exciting aspect of this dataset is that all 16 Fabs have the same CDR-H3 loop.

The 16 Fab structures were protonated using the Protonate3D tool (34, 35). Charge neutrality was ensured by utilizing the uniform background plasma approach in AMBER (36, 37). Using the tleap tool of the AmberTools20 (38) package, the crystal structures were soaked in cubic water boxes of TIP3P water molecules with a minimum wall distance of 10 Å to the protein (39). The structures were described with the AMBER force field 14SB (40). The antibody fragments were carefully equilibrated using a multistep equilibration protocol (41).

### Metadynamics Simulations

To enhance the sampling of the conformational space, well-tempered bias-exchange metadynamics (42–44) simulations were performed in GROMACS (45, 46) with the PLUMED 2 implementation (47). We chose metadynamics as it enhances sampling on predefined collective variables (CV). The sampling is accelerated by a history-dependent bias potential, which is constructed in the space of the CVs (42, 44, 48). As collective variables, we used a well-established protocol, boosting a linear combination of sine and cosine of the ψ torsion angles of all six CDR loops calculated with functions MATHEVAL and COMBINE implemented in PLUMED 2 (14, 19, 28, 47, 49, 50). As discussed previously, the ψ torsion angle captures conformational transitions comprehensively (51). The underlying method presented in this paper has been validated in various studies against a large number of experimental results. The simulations were performed at 300 K in an NpT ensemble using the GPU implementation of the pmemd module (52) to be as close to the experimental conditions as possible and to obtain the correct density distributions of both protein and water. We used a Gaussian height of 10.0 kJ/mol and a width of 0.3 rad. Gaussian deposition occurred every 1000 steps and a biasfactor of 10 was used. 500 ns of bias-exchange metadynamics simulations were performed for the prepared Fab structures. The resulting trajectories were aligned to the whole Fv and clustered with the program cpptraj (36, 53) using the average linkage hierarchical clustering algorithm with a RMSD cut-off criterion of 1.2 Å resulting in a large number of clusters. The cluster representatives for the antibody fragments were equilibrated and simulated for 100 ns using the AMBER 20 (38) simulation package. The number of clusters and the accumulated simulation time for the 16 Fab fragments are summarized in **SI Table S1**.

### Molecular Dynamics Simulations and Further Analyses

Molecular dynamics simulations were performed in an NpT ensemble using the pmemd.cuda module of AMBER 20 (36). Bonds involving hydrogen atoms were restrained with the SHAKE algorithm (54), allowing a time step of 2.0 fs. Atmospheric pressure (1 bar) of the system was set by weak coupling to an external bath using the Berendsen algorithm (55). The Langevin thermostat (56) was used to maintain the temperature during simulations at 300 K.

With the obtained trajectories we performed a time-lagged independent component analysis (tICA) using the python library PyEMMA 2 employing a lag time of 10 ns. tICA was applied to identify the slowest movements of the investigated Fab fragments and consequently to obtain a kinetic discretization of the sampled conformational space (57). tICA is a dimensionality reduction technique, detecting the slowest-relaxing degrees of freedom and facilitating the kinetic clustering, which is crucial for building a Markov-state model. It linearly transforms a set of high-dimensional input coordinates to a set of output coordinates, by finding a subspace of “good reaction coordinates”. Thereby, tICA finds coordinates of maximal autocorrelation at a given lag time. The lag time sets a lower limit to the timescales considered in the tICA and the Markov-state model. Accordingly, tIC1 and tIC2 represent the two slowest degrees of freedom of the systems.

Based on the tICA conformational spaces, thermodynamics and kinetics were calculated with a Markov-state model (58) by using PyEMMA 2, which uses the k-means clustering algorithm (59) to define microstates and the PCCA+ clustering algorithm (60) to coarse-grain the microstates to macrostates. Markov-state models are network models which provide valuable insights for conformational states and transition probabilities between them, as it is possible to sufficient accurately identify the boundaries between two states (58). The states are defined based on kinetic criteria, which allow to identify the boundaries between free energy wells. Basically, MSMs coarse-grain the system’s dynamics, which reflect the free energy surface and ultimately determine the system’s structure and dynamics. Thus, MSMs provide important insights and enhance the understanding of states and transition probabilities and facilitates a quantitative connection with experimental data (58, 61).

We performed tICA analyses and calculated Markov-state models for all 16 different germline pairs of the paratope and for all individual CDR loops following the IMGT nomenclature (62).

The sampling efficiency and the reliability of the Markov-state model (e.g., defining optimal feature mappings) can be evaluated with the Chapman-Kolmogorov test (63, 64), by using the variational approach for Markov processes (65) and monitoring the fraction of states used, since the network states must be fully connected to calculate probabilities of transitions and the relative equilibrium probabilities. To build the Markov-state model we used the backbone torsions of the respective CDR loop, defined 150 microstates using the k-means clustering algorithm and applied a lag time of 10 ns.

The canonical cluster representatives for each CDR loop, extracted from the PyIgClassify database (10), were projected into the free energy surfaces of all individual CDR loops. We then used the respective macrostate ensembles to investigate correlations between the different paratope states and the relative V_H_ and V_L_ orientations.

**Figure 1:**
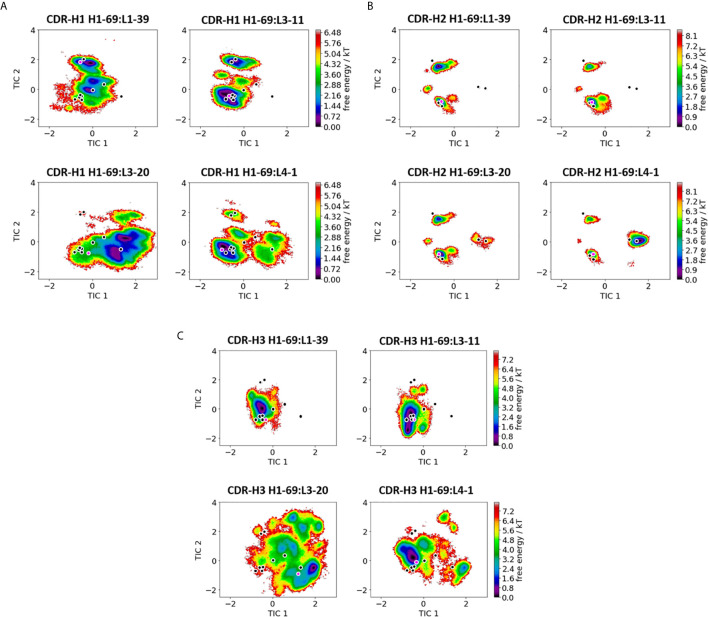
Comparison of all V_H_-CDR loops consisting of the heavy chain germline H1-69. **(A)** Shows the free energy surfaces of the CDR-H1 loop paired with different light chain germlines in the same coordinate system. The available canonical cluster structure representatives from the PyIg database for the CDR-H1 loop of length 13 are projected into the free energy surface and are depicted in black. In pink the respective crystal structures, which were used as starting structures are illustrated (PDB accession codes: 5I15, 5I16, 5I17 and 5I18). **(B)** Displays the resulting free energy landscapes for the CDR-H2 loop projected into the same coordinate system. The canonical cluster structures of the CDR-H2 loop of length 10 are projected and illustrated in black. The pink dot represents the starting X-ray structures for the simulations. **(C)** Shows the free energy landscapes of the CDR-H3 loop. As for the CDR-H3 loop no canonical cluster structures are available, we projected the 16 CDR-H3 loop crystal structures, which were investigated in this study into the free energy landscape. The pink dot shows the CDR-H3 loop starting structure for the simulations.

**Figure 2:**
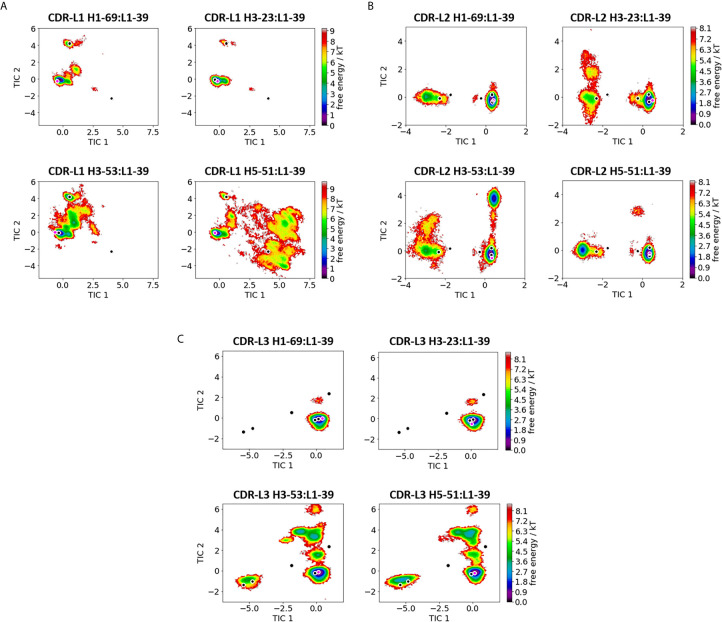
Comparison of all V_L_-CDR loops consisting of the heavy chain germline L1-39. **(A)** Shows the free energy surfaces of the CDR-L1 loop paired with different heavy chain germlines in the same coordinate system. The available canonical cluster structures for the CDR-L1 loop of length 11 are projected into the free energy surface and are depicted in black. In pink the respective crystal structures, which were used as starting structures are illustrated (PDB accession codes: 5I15, 5I19, 5I1E and 4KMT). **(B)** Displays the resulting free energy landscapes for the CDR-L2 loop projected into the same coordinate system. The canonical cluster structures of the CDR-L2 loop of length 8 are projected and illustrated in black. The pink dot represents the starting X-ray structures for the simulations. **(C)** Shows the free energy landscapes of the CDR-L3 loop. The canonical cluster representatives of the CDR-L3 loop, consisting of 9 residues, are projected into the free energy surfaces and colored in black. The pink dot shows the CDR-L3 loop starting structure for the simulations.

To quantify the CDR loop flexibilities, we performed a clustering for the individual CDR loops presented in **Figures 1**, **2**. To cluster the individual CDR loops, we aligned on the respective heavy or light chain. We used the average-linkage clustering algorithm and applied a RMSD distance cut-off criterion of 1.5 Å for the light chain CDR loops and a RMSD distance cut-off criterion of 2.5 Å for the heavy chain CDR loops. We decided to use a different distance cut-off criterion as the heavy chain CDR loops reveal a higher flexibility compared to the light chain CDR loops.

### Quantification of Sidechain Orientations and Flexibilities

To eradicate the effect of the backbone conformation on the sidechain orientation, we performed a residue-wise alignment. Therefore, the backbone nitrogen, Cα and carbonyl carbon atoms of the respective residue have to be aligned in all frames of the trajectory. In the next step, the vector from the Cα atom to the center of mass of the respective sidechain is calculated for every frame of the trajectory. These calculated vectors reflect the orientations and the flexibility of the sidechain during the simulation. To facilitate the comparison between the sidechain orientations of different residues, we hereby provide an internal coordinate system for every sidechain. To this end, we rotated the internal coordinate systems in a standardized orientation in the unit sphere: The average vectors from the Cα to the Cβ atoms are aligned with the x-axis and the Cα to the carbonyl carbon atoms are oriented in the xy-plane. Thus, the main advantage from our calculations is that we do not lose the information of the sidechain orientation and the flexibility.

### Relative V_H_ and V_L_ Orientations Using ABangle

ABangle is a computational tool (6, 20, 21, 32) to characterize the relative orientations between the antibody variable domains (V_H_ and V_L_) using six measurements (five angles and a distance). A plane is projected on each of the two variable domains. To define these planes, the first two components of a principal component analysis of 240 reference coordinates were used for V_H_ and V_L_ each. The reference coordinate set consists of Cα coordinates of eight conserved residues for 30 cluster representatives from a sequence clustering of the nonredundant ABangle antibody data set. The planes were then fit with those 240 coordinates, and consensus structures consisting of 35 structurally conserved Cα positions were created for the V_H_ and V_L_ domain. Between these two planes, a distance vector C is defined. The six measures are then two tilt angles between each plane (HC1, HC2, LC1, LC2) and a torsion angle (HL) between the two planes along the distance vector C (dc). The ABangle script can calculate these measures for an arbitrary Fv region by aligning the consensus structures to the found core set positions and fitting the planes and distance vector from this alignment. This online available tool was combined with an in‐house python script to reduce computational effort and to visualize our simulation data over time. The in‐house script makes use of ANARCI (66) for fast local annotation of the Fv region and pytraj from the AmberTools package (38) for rapid trajectory processing. To better visualize shifts in the relative V_H_-V_L_ interdomain orientation we performed the Gaussian kernel density estimation (KDE) on the HL angle, to obtain probability density distributions. To calculate the KDE we used the recently published implementation of KDE in C++ (67).

## Results

We applied a well-established protocol combining enhanced sampling techniques with classical molecular dynamics simulations to systematically elucidate the effect of different heavy and light chain pairings on the antibody binding site and the relative V_H_-V_L_ interface (14, 15). As starting structures, we used the available 16 Fab structures, which were generated combining four different heavy and four light chain germline genes, all originating from the same human germline library (68). All 16 Fab fragments have the same CDR-H3 loop sequence, while the other CDR loops vary in their loop length and sequence composition.

As described in the methods section, we performed 500 ns of bias-exchange simulations for all 16 Fabs. We clustered the trajectories individually and used the resulting cluster representatives as starting structures for each 100 ns of molecular dynamics simulations.

**SI Table S1** summarizes the obtained number of clusters and the aggregated simulation time for all 16 antigen-binding fragments. To directly investigate the effect of different heavy and light chain pairings, we compare the CDR loop dynamics of the different antibodies with the same heavy chain and light chains respectively.

We present the results of one heavy chain (H1-69) and one light chain germline in detail in **Figures 1**, **2**, while all other germline comparisons can be found in the supporting information (**SI Figures S1–S35**). **Figure 1** shows the free energy surfaces of the CDR-H1, CDR-H2 and CDR-H3 loops of four antibodies with the heavy chain germline H1-69 (PDB accession codes: 5I15, 5I16, 5I17 and 5I18 – **SI Table S1**). The free energy landscapes in **Figure 1A** show strong population shifts of the dominant solution structures of the CDR-H1 loop upon exchanging the paired light chain germlines. The strongest effect can be seen for the CDR-H1 loop of the H1-69:L3-20 (5I17) germline pairing. Even though, the CDR-H1 loop has the same length and sequence in all four free energy landscapes, we observe significant differences in flexibility, which are also reflected in differently sampled canonical clusters and shifts in dominant solution structures. The majority of available canonical clusters (H1-13) are present within our ensemble, only with varying probabilities. Especially interesting are the CDR-H1 loops of the H1-69:L3-20 and H1-69:L4-1 (5I17 and 5I18), as they sample an additional canonical cluster (H1-13-6), which is not captured within the ensembles of other CDR-H1 loops. **Figure 1B** illustrates the free energy landscapes of the CDR-H2 loops. Also, for the CDR-H2 loop an effect of different light chain pairings on the respective CDR-H2 loop ensembles can be identified. In line with the observations for the CDR-H1 loop, we find strong pairing specific population shifts of the CDR-H2 loops. This is especially true for the H1-69:L3-20 and H1-69:L4-1 (5I17 and 5I18) Fab fragments, where we sample two additional canonical clusters (H2-10-2 and H2-10-4), compared to the other CDR-H2 loops consisting of germline H1-69. The free energy landscapes of the CDR-H3 loop are depicted in **Figure 1C**. While the CDR-H3 loops of H1-69:L1-39 and H1-69:L3-11 (5I15 and 5I16) cover a similar conformational space, again H1-69:L3-20 and H1-69:L4-1 (5I17 and 5I18) differ substantially in their flexibility and state populations. As for the CDR-H3 loop no canonical clusters could be assigned, we projected the available 16 X-ray structures into the tICA space, which already reveal a high conformational diversity. The majority of these 16 Fab crystal structures are present within the obtained CDR-H3 loop ensembles in solution and additional pairing specific CDR-H3 loop solution structures can be observed. The results presented in **Figure 1** clearly show a strong correlation between the three heavy chain CDR loops and reveal a strong dependency of the V_H_ - CDR loop ensembles on the respective light chain pairing.

On the other hand, **Figure 2** depicts the direct comparison of all V_L_ - CDR loop ensembles containing the L1-39 germline (5I15, 5I19, 5I1E and 4KMT). The free energy surfaces of the CDR-L1 loop projected into the same coordinate system clearly show a strong influence of paired heavy chains. While the two antibodies H1-69:L1-39 and H3-23:L1-39 (5I15 and 5I19) with the highest experimentally determined melting temperatures (**SI Table S1**) are mainly restricted to one dominant minimum in solution, H3-53:L1-39 and H5-51:L1-39 (5I1E and 4KMT) reveal a substantially higher flexibility, which is also reflected in the higher number of clusters (**SI Table S2**). Apart from the presented free energy surfaces, we quantified flexibility by clustering on the individual CDR loops and aligning on the respective heavy or light chain. For the clustering we used the average linkage clustering algorithm and applied a RMSD distance cut-off criterion of 2.5 Å for all heavy chain CDR loops and a RMSD distance cut-off criterion of 1.5 Å for all light chain CDR loops (**Table S2**). Three canonical clusters exist for the CDR-L1 loop with a loop length of 11 residues (L1-11). Two of these three canonical clusters contain κ light chain antibodies (L1-11-1, L1-11-2), while the third is composed of mainly λ light chain antibodies (L1-11-3) (10, 11). Astonishingly, depending on the paired heavy chain germline, all three canonical clusters become accessible, as can be seen for the H5-51:L1-39 antibody. **Figure 2B** shows the free energy landscapes of the CDR-L2 loops with the same L1-39 germline. Also, for the CDR-L2 loop we observe that the same sequence can adopt different solution structures, depending on correlated CDR loop movements and germline pairings. We do not only sample the majority of available canonical clusters, but also identify other dominant minima in solution. The CDR-L3 loop conformational space is illustrated in **Figure 2C** and clearly shows, in line with all other observations, germline-pairing specific ensembles in solution. Again, the flexibility of the two most stable Fab variants (5I15 and 5I19) reveal only one distinct CDR-L3 loop minimum, while the other two Fabs cover a broader conformational space and additional minima in solution. All of these individually described germline-pairing specific conformational changes in the CDR loops can be combined to paratope states in solution.

**Figure 3:**
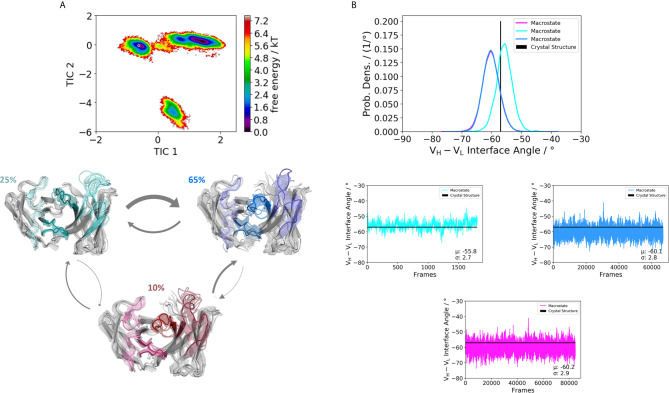
Germline pairing specific paratope states in solution for the H1-69:L1-39 (5I15) Fab. **(A)** Shows the resulting paratope states, the free energy landscape, the macrostate ensembles and the respective V_H_ and V_L_ orientations for the 5I15 antibody. The thickness of the arrows corresponds to the transition timescales. The ticker the arrows the faster the transition. The macrostate ensembles are arranged according to the shape of the tICA. **(B)** We also observe a significant shift in the interdomain angle distributions upon conformational changes in the paratope, which are visualized as probability density distributions. The starting X-ray structure for the underlying simulations is depicted as black dot in the free energy surfaces and as black line in the plots showing the V_H_-V_L_ angle distributions.

Furthermore, **Figure 3** illustrates the respective paratope free energy surface of the Fab H1-69:L1-39 (5I15), with the corresponding macrostate representatives, state probabilities and V_H_-V_L_ interface angle distributions. We observe a shift in the relative V_H_-V_L_ distribution upon conformational rearrangements in the paratope and identify other dominant paratope ensembles in solution (**Figure 3B**). Additionally, we investigated the Fab H5-51:L1-39 (4KMT), which has the same light chain germline as the 5I15.

**Figure 4:**
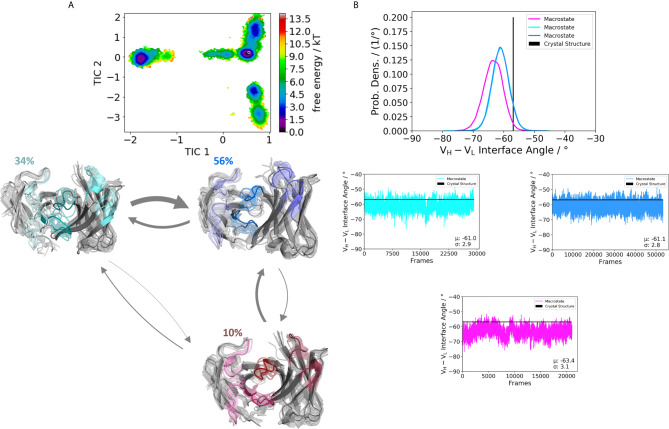
Germline pairing specific paratope states in solution for the H5-51:L1-39 (4KMT) Fabs. **(A)** Illustrates the paratope states, free energy surface, macrostate ensemble and the respective V_H_ and V_L_ orientations for the 4KMT antibody. **(B)** We observe shifts in the V_H_ and V_L_ orientations upon rearrangements in the paratope, which are visualized as probability density distributions. The starting X-ray structure for the underlying simulations is depicted as black dot in the free energy surfaces and as black line in the plots showing the V_H_-V_L_ angle distributions.

**Figure 5:**
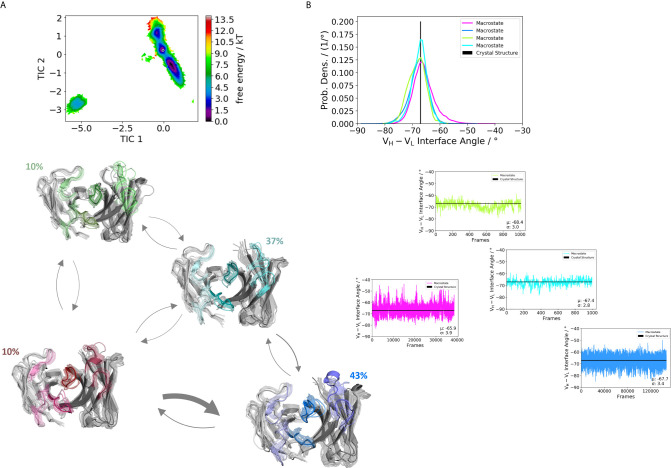
Germline pairing specific paratope states in solution for the H3-23:L3-20 (5I1C) Fab. **(A)** Shows the paratope states, the free energy landscape, the macrostate ensembles and the respective VH and VL orientations for the 5I1C antibody. The thickness of the arrows corresponds to the obtained transition timescales. The thicker the arrow the faster the transition. The macrostate ensembles are arranged according to the shape of the tICA. **(B)** We also observe a small shift in the interdomain angle distributions upon conformational changes in the paratope, which is visualized as probability density distributions. The starting X-ray structure for the underlying simulations is depicted as black dot in the free energy surfaces and as black line in the plots showing the VH-VL angle distributions.

**Figure 6:**
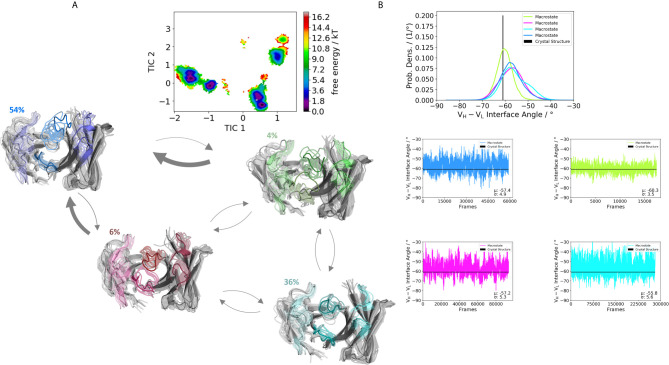
Germline pairing specific paratope states in solution for the H3-53:L4-1 (5I1I) Fab. **(A)** Illustrates the paratope states, free energy surface, macrostate ensemble and the respective V_H_ and V_L_ orientations for the 5I1I antibody. **(B)** Also, here we find small shifts in the V_H_ and V_L_ orientations upon rearrangements in the paratope. Especially interesting is the fact that different germline pairings favor specific interdomain orientations. The highest variations in these interdomain angle distributions can be observed for the 5I1I, which is also one of the least stable Fab fragments. The starting X-ray structure for the underlying simulations is depicted as black dot in the free energy surfaces and as black line in the plots showing the V_H_-V_L_ angle distributions.

We obtained three paratope states and find changes in the relative V_H_-V_L_ orientations upon rearrangements in the CDR loops (**Figure 4B**). By comparing the two Fabs 5I15 and 4KMT, we observe a small shift in the interface angle upon different heavy chain germline pairings. The crystal structure interface angle differed only 1.8°, while we capture a substantially broader conformational variability in this angle and observe a shift between different macrostates of up to 8°. **Figure 5** illustrates the paratope states of H3-23:L3-20 Fab (5I1C), which represents an example of completely differently paired heavy and light chain germlines, compared to **Figure 3**. Here, we observe four paratope states with small shifts in the V_H_-V_L_ interface angle distributions of about 3° as a consequence of conformational changes in the paratope (**Figure 5B**). **Figure 6** shows the free energy surfaces and interface angle distributions of the paratope states of the 5I1I (H3-53:L4-1). Compared to the results in **Figures 3**–**5**, we again find pairing specific interface angles (**Figure 6B**). The relative interdomain orientation calculated with ABangle, which is determined by six measures, also reveals substantially higher variations (6). This is in line with the experimentally determined stability measurements for the 5I1I, as it is one of the least stable variants (**SI Table S1**) (68). We provided all six interdomain orientation descriptors, for all 16 Fab fragments in **SI Figure S36**, showing the mean and standard error. We find that the variances in the distance vector between the two domains might be indicators for thermal stability, as we see a correlation between the experimentally determined melting temperatures and the fluctuations in the distance (**SI Figure S37**).

Apart from capturing the backbone rearrangements of the CDR loops we were also interested in investigating pairing specific sidechain conformations and their respective flexibilities.

**Figure 7:**
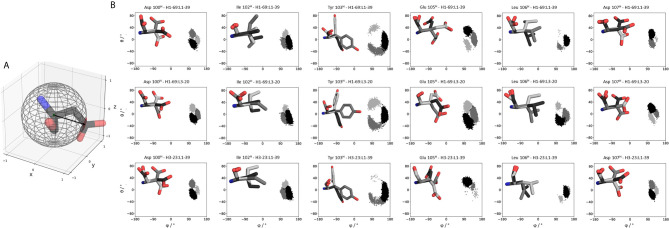
Sidechain flexibilities of the CDR-H3 loop. **(A)** Exemplary illustration of an amino acid projected into the sphere from which the polar coordinates (θ and φ) are calculated. **(B)** The same CDR-H3 loop can adopt pairing specific CDR-H3 loop conformations. Here we provide a detailed analysis of sidechain orientations and which sidechains are strongly influenced by different light and heavy chain pairings respectively. We also color-coded the different sidechain orientations according to their populations: dark grey represents the highest populated sidechain conformation, while in light grey the least populated one is illustrated. On the left, the respective sidechain orientations and conformations are depicted and colored respectively. The X and Y axes show the projection of the vectors on the polar angle and azimuth angle on the surface of a unit sphere (spherical coordinates). This allows to determine not only the flexibility of the sidechain but also the orientation. A more detailed description can be found in the methods section.

**Figure 7** depicts the conformational states of the different CDR-H3 loop sidechains. We analyzed the sidechain flexibilities of the CDR-H3 loop, since all 16 Fabs share the same CDR-H3 loop sequence. We included in **Figure 7** the CDR-H3 loop chains of the 5I15, 5I17 and 5I19. We chose these Fabs as they either differ in their paired light chain or heavy chain. As a consequence of different pairings, the strongest population shifts and biggest conformational variations of the CDR-H3 loop can be seen for the glutamate 105, leucine 106, aspartate 107 and tyrosine 103. The reason for the different sidechain conformations can be explained by different interaction partners in the light and heavy chain, which consequently also contribute to distinct CDR loop conformations and interface orientations.

## Discussion

This study presents a structural and dynamic characterization of a phage germline library, by investigating the effect of different heavy and light chain pairings on the antibody paratope and the V_H_-V_L_ interface distributions. We provide a new understanding of the antibody paratope and show that both sidechain and backbone CDR conformations can vary depending on the paired heavy or light chain. Antibody CDR loops are flexible and can adopt various distinct conformations in solution (15). Recent studies also revealed that various biophysical properties of antibodies are governed by their conformational diversity (14, 31, 69–71). To capture this high flexibility and diversity of the CDR loops, they need to be described as conformational ensembles in solution (18). For all CDR loops conformational transitions between different canonical clusters and additional dominant solution structures have been observed. In previous simulation studies these conformational transitions between different CDR loop conformations have been shown to occur in the micro-to-millisecond timescale (15, 18). **Figures 1**, **2** are in perfect agreement with these findings and emphasize that one single static structure is not sufficient to capture the high conformational variability of the CDR loops. **SI Figures S1–S35** show very similar findings and consistently show pairing specific CDR loop conformations and differently favored canonical cluster structures. The pink dot projected into the free energy surfaces represents the starting X-ray structure. However, the structure characterizing an antibody the best is the dominant conformation in solution, which not necessarily coincides with the apo X-ray structure. Especially, since the apo crystal structure can be distorted by crystal packing effects.

Additionally, we show that even identical sequences can adopt different germline-specific conformations depending on the type of paired heavy and light chain, respectively. This is especially interesting for the CDR-H1 and CDR-H2 loop, as these loops are not directly interfacial with the paired light chain, however, their conformational variability is still affected. The reason for that is the strong structural correlation between the CDR-H3 and the CDR-H1 loop. These results emphasize that a different understanding from single static canonical structures to dynamic ensembles in solution is inevitable, as antibody specificity and affinity are strongly dominated by the shape and dynamics of the binding site.

Apart from the CDR loops also the relative interdomain and elbow angle orientations have been shown to contribute substantially to the flexibility of the antigen-binding site (20, 31, 32, 71, 72). By combining all these findings, the antibody binding site exists as multiple paratope states in solution, which are characterized by strongly correlated CDR loop and interdomain movements (**SI Figure S40**) (33). These backbone rearrangements in the paratope occur in the micro-to-millisecond timescale. **Figures 3**–**6** depict paratope states in solution of different germline pairings and do not only describe shifts in the V_H_-V_L_ interface distributions, but also reveal germline pairing specific interface orientations. These characteristic V_H_-V_L_ orientations are defined by CDR loop backbone and sidechain rearrangements, which result in unique interactions stabilizing the V_H_-V_L_ interface (33, 73). Astonishingly, we observe the highest variations in the relative V_H_-V_L_ distributions for the least stable Fab. This can be explained by different interactions between the CDR loops and higher variability in interfacial contacts.

Thus, apart from interdomain interactions of the CDR loops, also contacts within the interface are involved in V_H_-V_L_ pairing. Among all human antibodies exist a small set of interdomain interactions that are conserved (L-Gln38 und H-Gln39, H-Leu45 and L-Phe98, L-Pro44 and H-Trp103, L-Ala43 and H-Tyr91). These interactions ensure a stable structural basis to the V_H_-V_L_ dimer to even tolerate variations in the amino acid sequence of the CDR loops, in particular the CDR-L3 and CDR-H3 loops (68). The occurrence of these contacts for all the investigated 16 Fab fragments is illustrated in **SI Table S3**. We find that upon changes in the relative V_H_-V_L_ interdomain orientation, these core interactions are maintained, however the duration and fluctuations in these contacts can be higher, depending on the paired germlines of the respective antibody (**SI Table S3**). By considering not only the occurrence of the core interactions, but actually the fluctuations of all interdomain interactions formed between the differently paired heavy and light chains, we find that also the variability of the contacts in the interface are determinants for stability (**SI Figure S38**). While the paratope states presented in **Figures 3**–**6** kinetically and thermodynamically describe the backbone rearrangements of the CDR loops, **Figure 7** displays the influence of different heavy and light chain pairings on the resulting sidechain dynamics of the CDR-H3 loop. We observe substantial shifts in the populations of certain sidechain conformations of the identical CDR-H3 loop as a consequence of different germline pairings. Residue E105, as well as the D100 and D107 form hydrogen bond interactions with the neighboring CDR-L1, CDR-L2 and CDR-L3. For the Fabs 5I15 and 5I19, which are paired with the same light chain, we observe hydrogen bond interactions of the CDR-H3 loop residues (D100, E105 and D107) with the tyrosine (Y32) and the asparagine (N34) located at the CDR-L1 loop (**SI Figure S39**). Additionally, also the backbone of leucine 106 interacts with the sidechain of tyrosine 32. A crucial residue that distinguishes the L1-39 germline from all other investigated germline light chains is the N34 at the end of the CDR-L1 loop (**Table S4**). This residue strengthens the interaction network in the V_H_-V_L_ interface and thus, contributes to the high stability of both 5I15 and 5I19. Additionally, also the glutamine 55 (Q55) located at the CDR-L2 loop forms a hydrogen bond with the D107 of the CDR-H3 loop (**SI Figure S39**). Both residues are unique for the L1-39 germline and a more detailed and quantitative analysis of the interaction network formed is depicted in **Table S4**. Astonishingly, even though the L4-1 light chain contains a glutamate at position 55, no hydrogen bonds or salt bridges are formed with the heavy chain, as all potential interaction partners in the close proximity are negatively charged (**SI Table S5**). Furthermore, the CDR-L3 loop contains unique residues, which play a central role in stabilizing the interface between the heavy and the light chain. Residue S91 forms hydrogen bonds with Y103 and E105, respectively, and thereby contributes to stabilize the interface between the two chains. Q89 makes mainly backbone interactions with the CDR-H3 loops. Another aspect that might contribute to the increase in Fab stability of germline L1-39, compared to other germlines, is that the CDR-L3 loop contains smaller residues at position 91 and 94, which allow more room to accommodate the CDR-H3 loop (68). Some of these key residues contributing to specific interdomain and CDR loop conformations are missing in the 5I17 Fab (H1-69:L3-20). Even though Y103 and D107 of the CDR-H3 loop can still form an interaction with Y32 of the CDR-L1 loop, the key interaction partner N34 at the end of the CDR-L1 loop is missing, which might contribute for the decrease in stability (**SI Table S4**). Also, instead of smaller residues in the center of the CDR-L3 loop, we find bulky residues, which might be less beneficial for the interplay with the CDR-H3 loop. Thus, interactions with the V_L_-CDR loops can influence the sidechain flexibility of the CDR-H3 loop and help to elucidate structural determinants for differences in stability.

Apart from the interdomain interactions of the CDR loops that substantially influence the V_H_-V_L_ interface, also certain framework residues have been discussed to have an effect on the paratope (24, 26, 33, 71, 72, 74–77). Various studies have already investigated the role of framework mutations on the CDR loops and the relative V_H_-V_L_ interdomain orientations based on X-ray structures (68, 78, 79). Even allosteric effects involving mutations in the C_H_1-C_L_ and the elbow angle have been reported to impact the antibody binding site and consequentially affinity and specificity (24, 26, 33, 71, 72, 74–77). In particular, residue 71^H^ [Kabat nomenclature (80)], has been shown to co-determine the canonical conformation of the CDR-H2 loop, according to whether there is a bulky residue or a small side-chain present and thus bringing the CDR-H1 and CDR-H2 loops closer to each other (27, 30, 81). Especially interesting is that the 71^H^ residue is part of the Vernier-zone residues, which have been reported to play a critical role in the humanization process and for rational design of antibodies in general as they can influence antibody specificity and affinity (73, 79, 82, 83). Differences in these framework residues might contribute to the distinct backbone and sidechain dynamics observed in the 5I15 and 5I19. The 5I19 Fab contains an arginine at Kabat position 71^H^, while 5I15 has an alanine at this position. Even though they have the same CDR-H2 loop length, distinct CDR-H2 loop conformations can be observed. What becomes apparent is that already single amino acid residues can result in changes in the dynamics of the whole paratope. Thus, to determine the influence of distinct heavy and light chain germline pairings, dominant solution structures should be considered. This dataset was particularly notable, because the identical CDR-H3 loop grafted on different heavy chains and paired with diverse light chains allowed to directly compare the obtained dynamics and ensembles of this loop. We find that different heavy and light chain pairings result in different CDR-H3 loop dynamics, which can also lead to different paratope states favoring distinct interdomain orientations. The antibodies investigated were designed and chosen to study the influence of different germline pairings. This synthetic human germline library lacks binding data but provides very valuable structural information showing no obvious difference to natural antibodies. Thus, despite the lack of binding data, we assume that our findings are also applicable to natural antibodies. The presented results show that there are indeed cases where considering unpaired sequences is not sufficient to structurally and dynamically understand the respective antibody functions and properties.

The flexibility of the antibody binding site has already been considered in antibody structure prediction and in antibody-antigen docking (84). However, characterizing conformational ensembles obtained from molecular dynamics simulations, allows to identify the dominant structure in solution and to retain the probabilities of the respective conformations. Thus, especially antibody-antigen docking might profit from including these probabilities, as not every conformation is equally probable and involved in the antigen-binding process. As the dominant solution structure has already been shown to frequently coincide with the binding competent conformation, ensembles can also guide the antibody humanization process, by elucidating the influence of the antibody framework or single point mutations on the paratope (85, 86). Thereby, conformational shifts and differences in flexibilities might be indicators for changes in antigen-recognition and/or differing biophysical properties such as specificity, which would allow to anticipate unfavorable effects upon antibody humanization (28, 87).

## Conclusion

In conclusion, we observe that identical loop sequences can result in distinct conformational CDR loop ensembles, depending on the paired heavy or light chain, respectively. Different heavy and light chain pairings do not only affect the CDR loop backbone and sidechain conformations but also favor specific V_H_-V_L_ interface orientations.

However, we emphasize that sequence information alone is not sufficient to describe the strongly structurally correlated CDR loop dynamics and V_H_-V_L_ pairing specific conformational states. We find germline pairing specific paratope states in solution that should lead to a change in the field of antibody engineering and design as they escape the view of single static structures to ensembles in solution, which are characterized by correlated CDR loop rearrangements and specific V_H_-V_L_ interface orientations. Additionally, we discuss potential determinants for stability and find germline-specific interactions in the CDR loops which help to explain differences in stability. These kinetically dominant conformational ensembles in solution do not only help to elucidate the effect of different heavy and light chain pairings but can further be used to fine-tune antibodies in terms of their specificity and developability and might lead to improvements of protein-protein docking and antibody humanization.

## Data Availability Statement

The original contributions presented in the study are included in the article/**Supplementary Material**. Further inquiries can be directed to the corresponding author.

## Author Contributions

MF-Q performed research and wrote the manuscript. KK, LB, JL, and PQ performed research and analyzed data. KL, GG, AB, and HK advised and supervised the research. All authors contributed to the article and approved the submitted version.

## Funding

This work was supported by the Austrian Science Fund (FWF) *via* the grants P30565, P30737 and P30402, as well as DOC 30. Furthermore, this project has received funding from the European Union’s Horizon 2020 Research and Innovation Program under grant agreement no. 764958.

## Conflict of Interest

The authors declare that the research was conducted in the absence of any commercial or financial relationships that could be construed as a potential conflict of interest.

## Publisher’s Note

All claims expressed in this article are solely those of the authors and do not necessarily represent those of their affiliated organizations, or those of the publisher, the editors and the reviewers. Any product that may be evaluated in this article, or claim that may be made by its manufacturer, is not guaranteed or endorsed by the publisher.
