# Supplementary material for: Germline-Dependent Antibody Paratope States and Pairing Specific VH-VL Interface Dynamics
Source: Front Immunol. 2021 Aug 10;12:675655. doi: 10.3389/fimmu.2021.675655 (PMC8382685; doi:10.3389/fimmu.2021.675655)
Supplement: Supplementary file 1 [file DataSheet_1.docx]

**Supporting Information**

Table S1: Summary table of the investigated germline pairings with the respective experimentally determined melting temperatures.


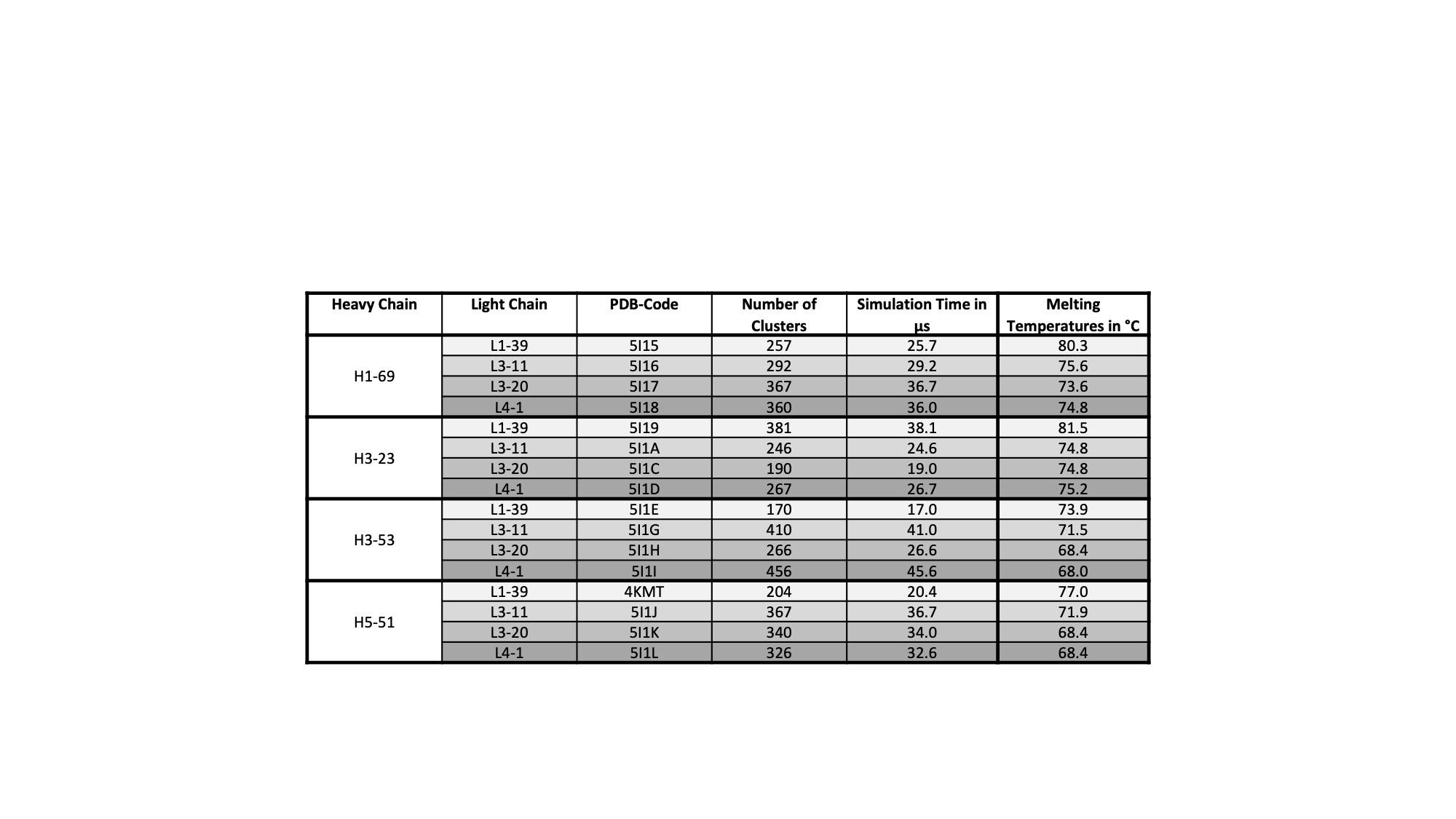


**
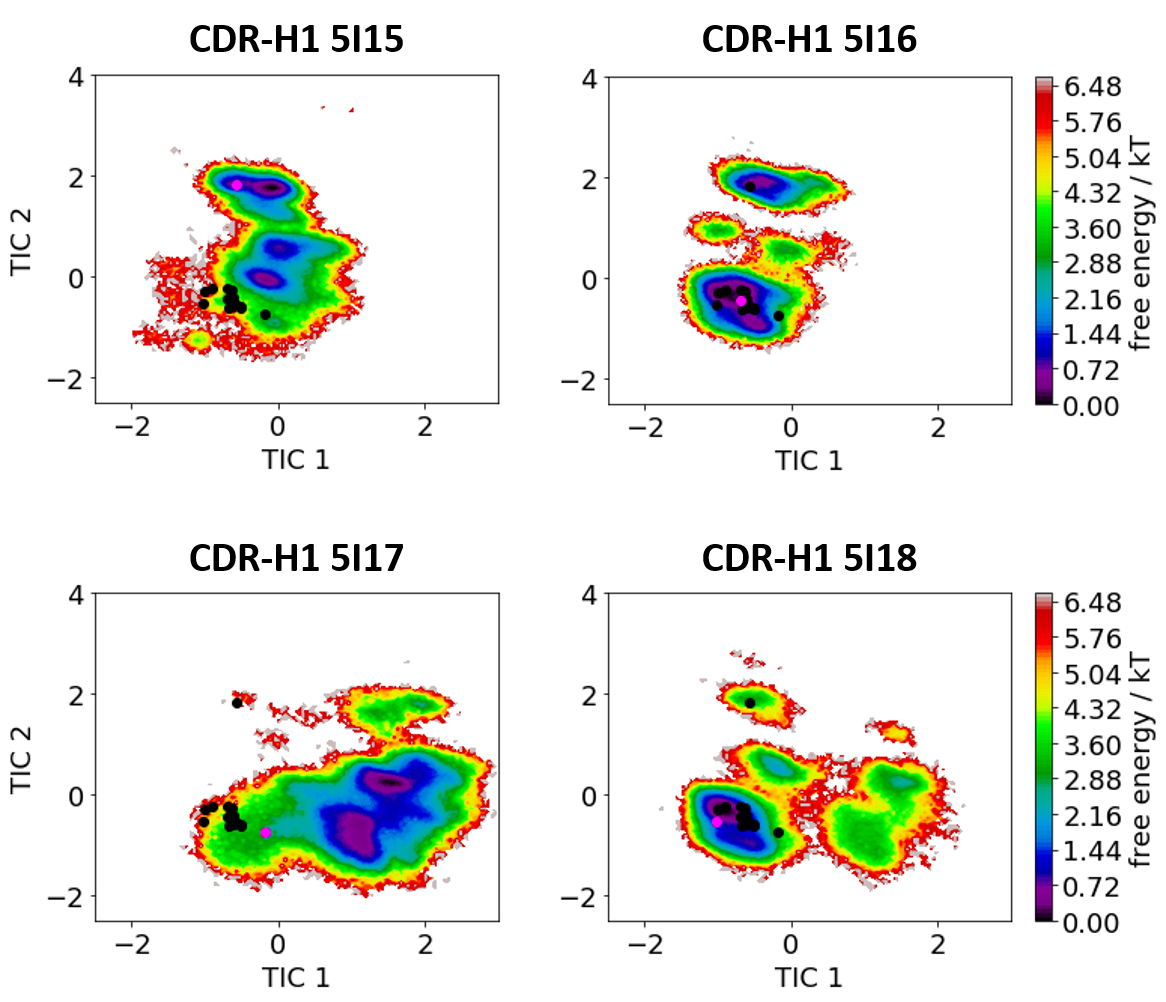
**

SI Figure S1: Comparison of the CDR-H1 loops consisting of the heavy chain germline H1-69. The free energy surface of the CDR-H1 loop paired with different light chain germlines in the same coordinate system is illustrated. The available 16 CDR-H1 loop conformations, resulting from different heavy and light chain pairings, are depicted in black. In pink the respective crystal structures, which were used as starting structures are illustrated (PDB accession codes: 5I15, 5I16, 5I17 and 5I18).

**
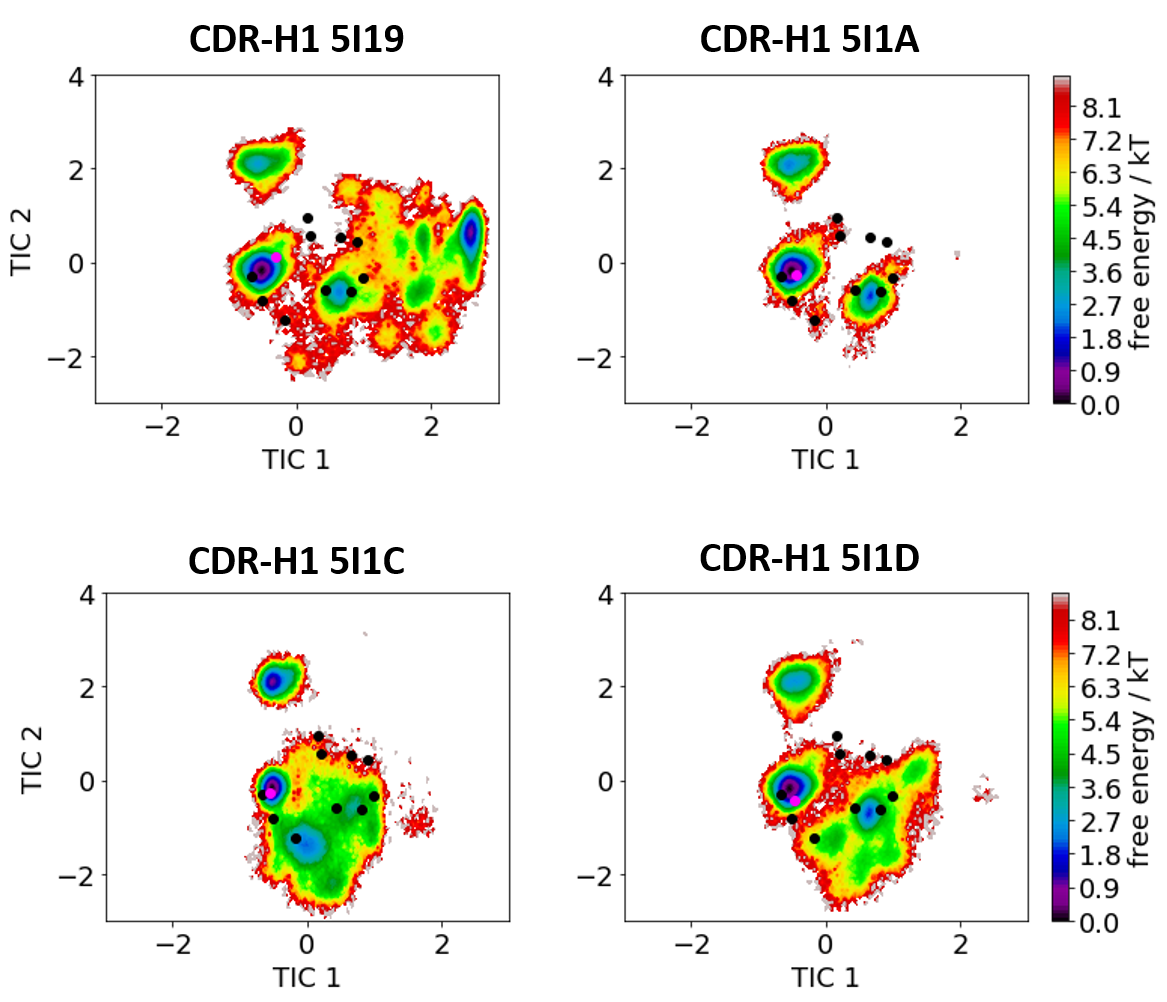
**

SI Figure S2: Comparison of the CDR-H1 loops consisting of the heavy chain germline H3-23. The free energy surface of the CDR-H1 loop paired with different light chain germlines in the same coordinate system is illustrated. The available canonical cluster structure representatives for the CDR-H1 loop of length 13 are projected into the free energy surface and are depicted in black. In pink the respective crystal structures, which were used as starting structures are illustrated (PDB accession codes: 5I19, 5I1A, 5I1C and 5I1D).

**
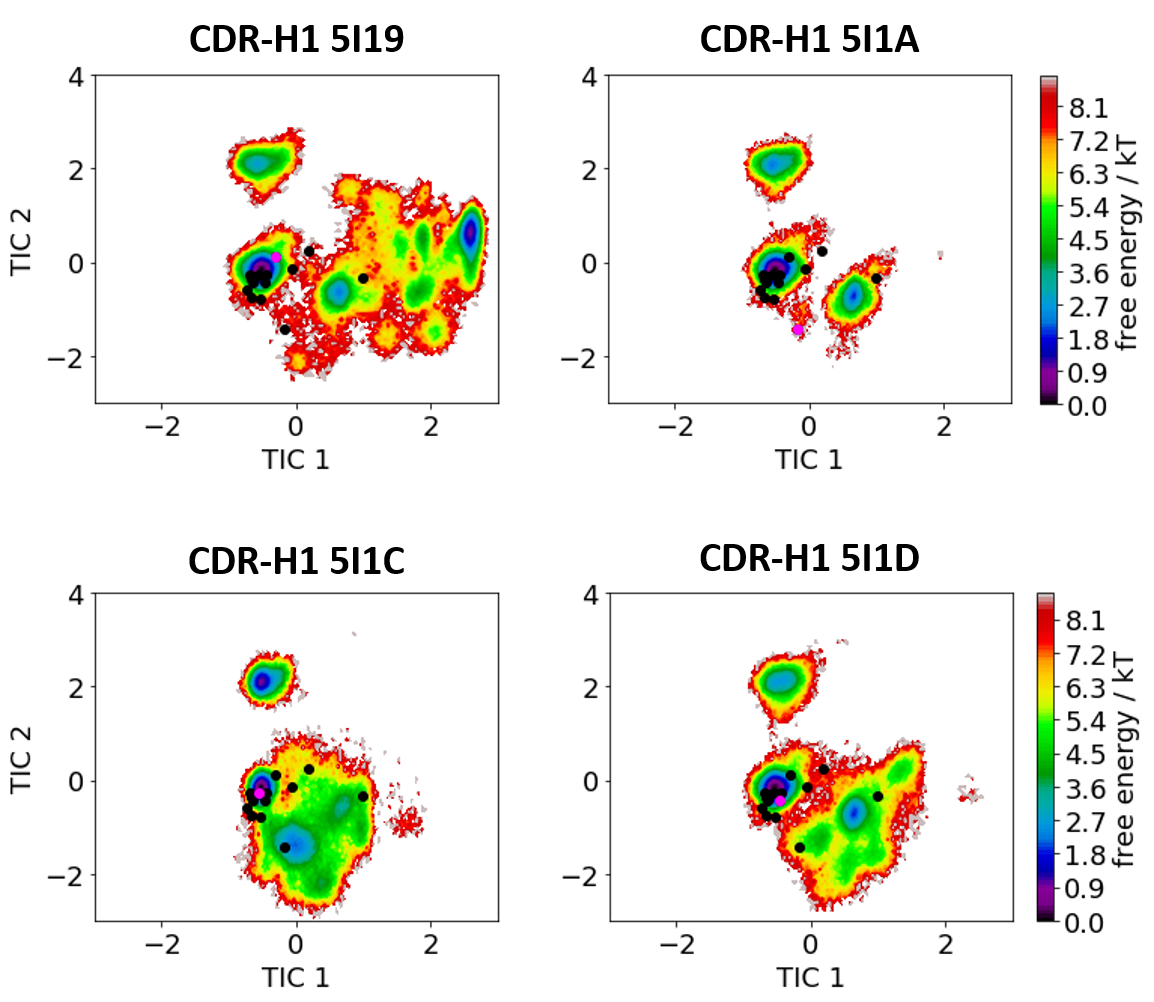
** SI Figure S3: Comparison of the CDR-H1 loops consisting of the heavy chain germline H3-23. The free energy surface of the CDR-H1 loop paired with different light chain germlines in the same coordinate system is illustrated. The available 16 CDR-H1 loop conformations, resulting from different heavy and light chain pairings, are depicted in black. In pink the respective crystal structures, which were used as starting structures are illustrated (PDB accession codes: 5I19, 5I1A, 5I1C and 5I1D).

**
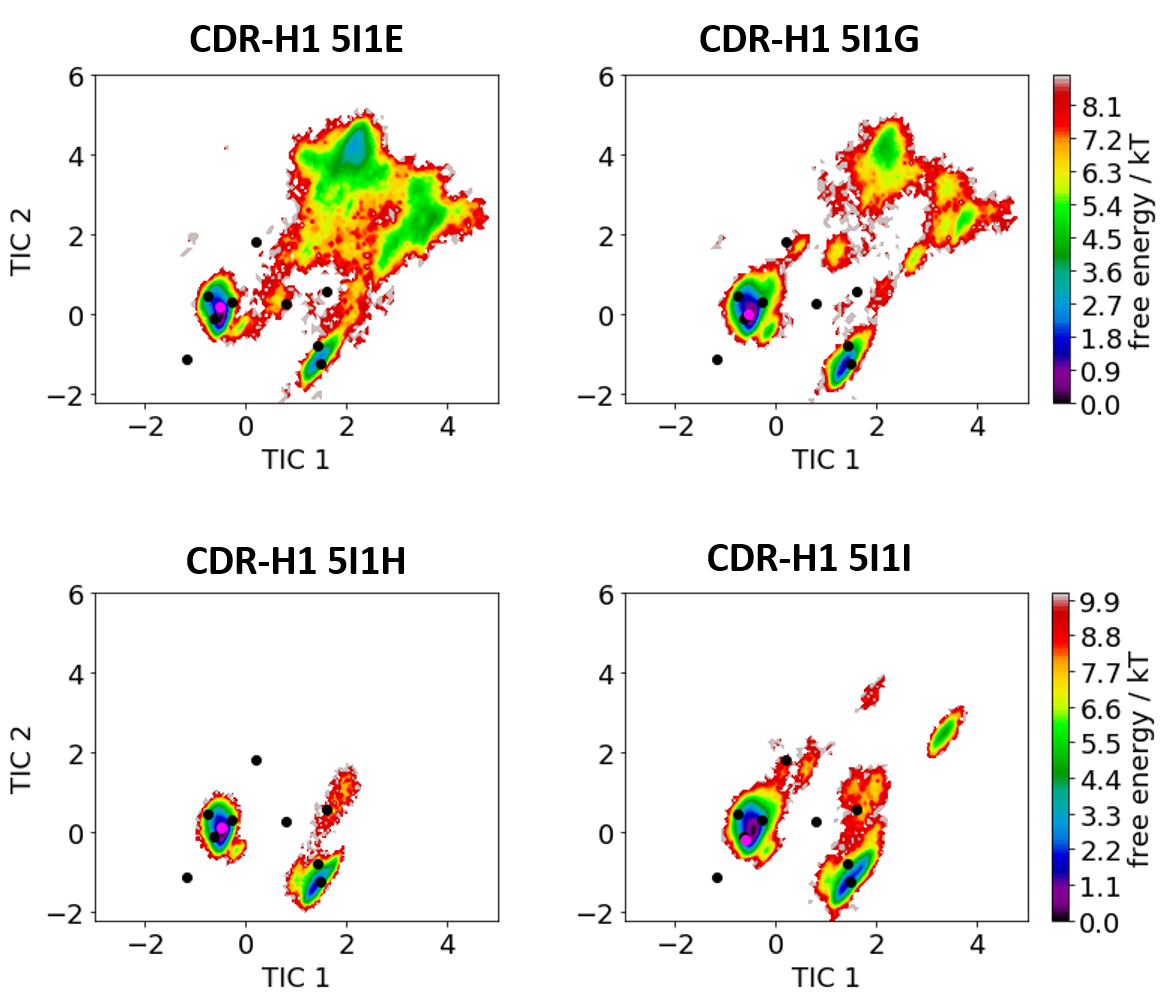
**

SI Figure S4: Comparison of the CDR-H1 loops consisting of the heavy chain germline H3-53. The free energy surface of the CDR-H1 loop paired with different light chain germlines in the same coordinate system is illustrated. The available canonical cluster structure representatives for the CDR-H1 loop of length 13 are projected into the free energy surface and are depicted in black. In pink the respective crystal structures, which were used as starting structures are illustrated (PDB accession codes: 5I1E, 5I1G, 5I1H and 5I1I).

**
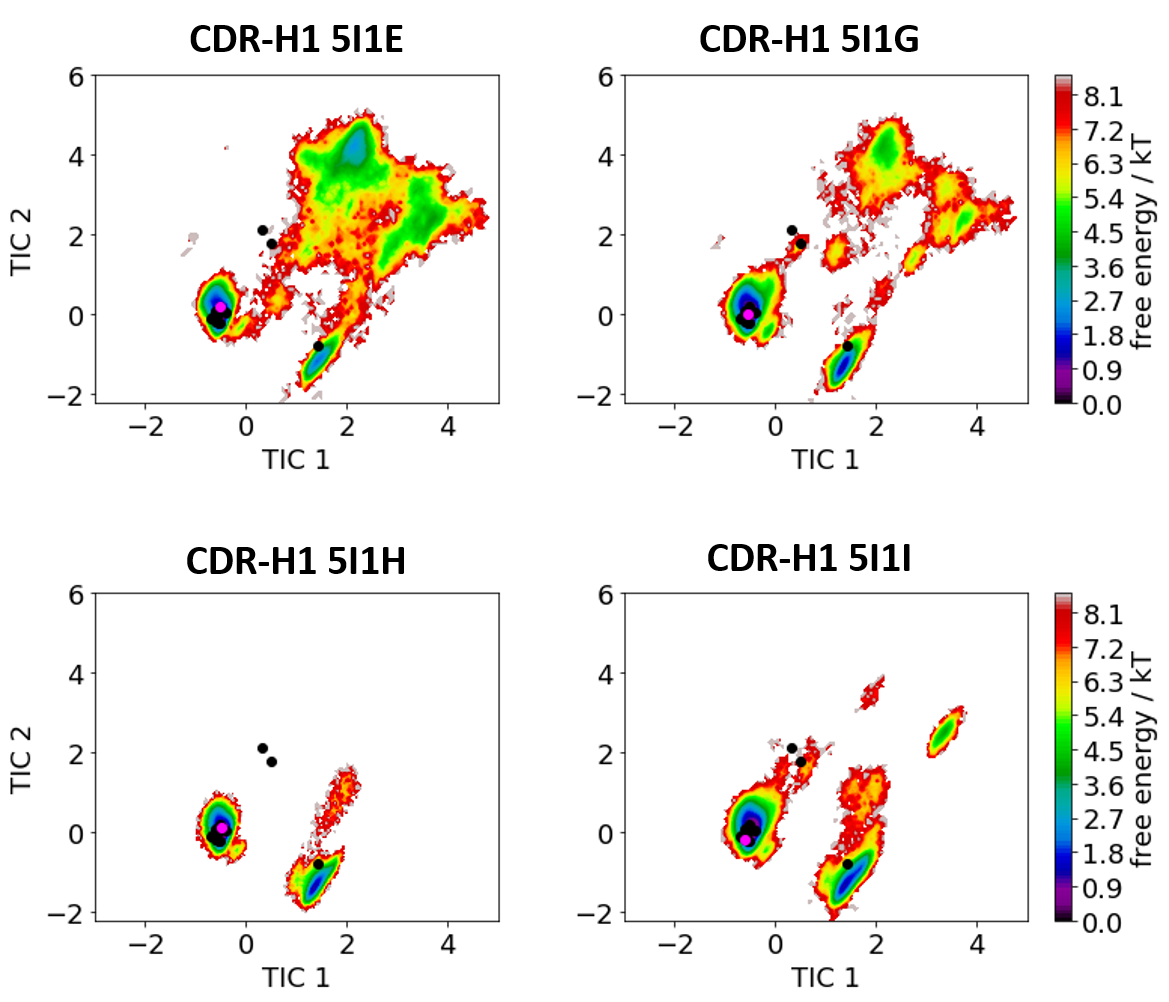
**

SI Figure S5: Comparison of the CDR-H1 loops consisting of the heavy chain germline H3-53. The free energy surface of the CDR-H1 loop paired with different light chain germlines in the same coordinate system is illustrated. The available 16 CDR-H1 loop conformations, resulting from different heavy and light chain pairings, are depicted in black. In pink the respective crystal structures, which were used as starting structures are illustrated (PDB accession codes: 5I1E, 5I1G, 5I1H and 5I1I).

**
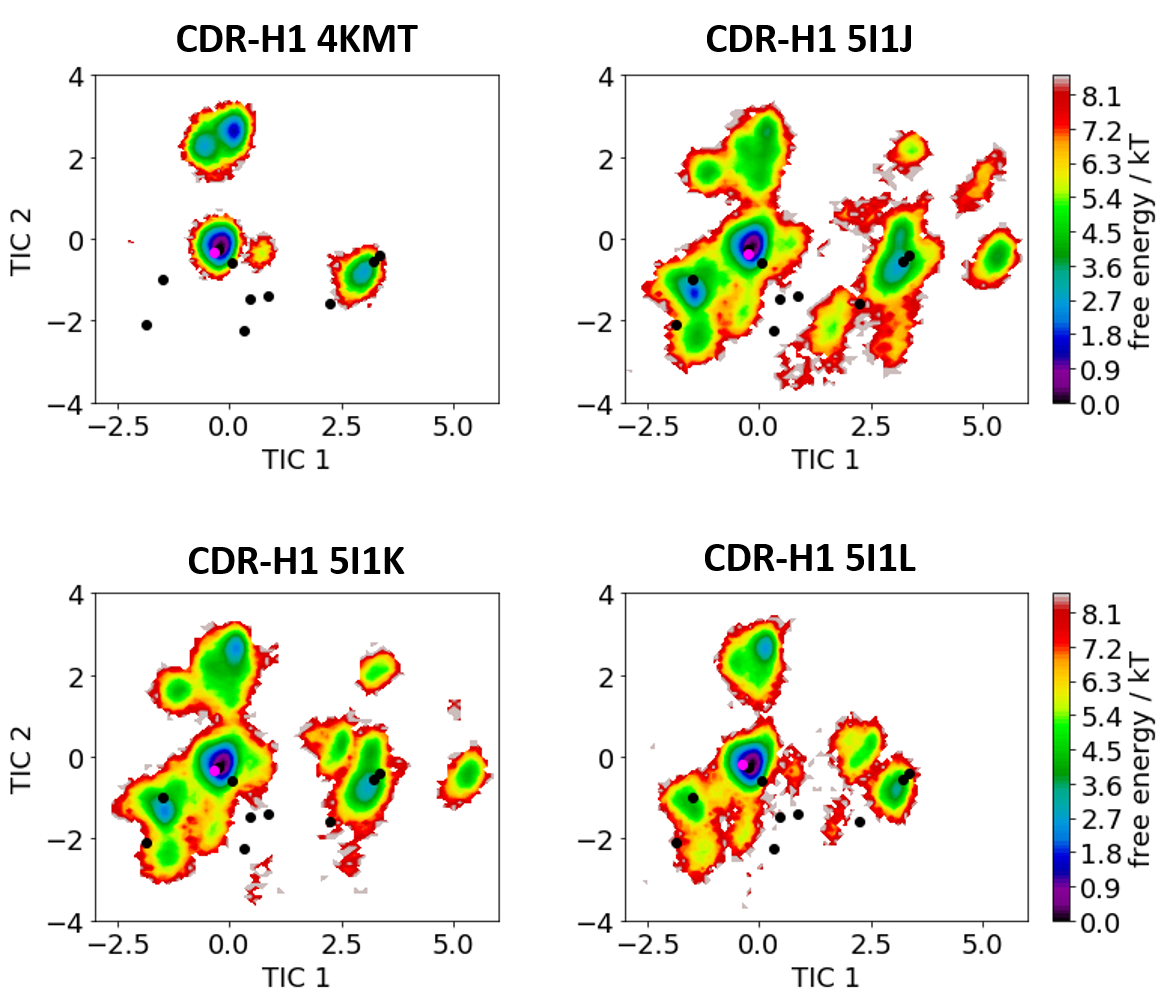
**

SI Figure S6: Comparison of the CDR-H1 loops consisting of the heavy chain germline H5-51. The free energy surface of the CDR-H1 loop paired with different light chain germlines in the same coordinate system is illustrated. The available canonical cluster structure representatives for the CDR-H1 loop of length 13 are projected into the free energy surface and are depicted in black. In pink the respective crystal structures, which were used as starting structures are illustrated (PDB accession codes: 4KMT, 5I1J, 5I1K and 5I1L).

**
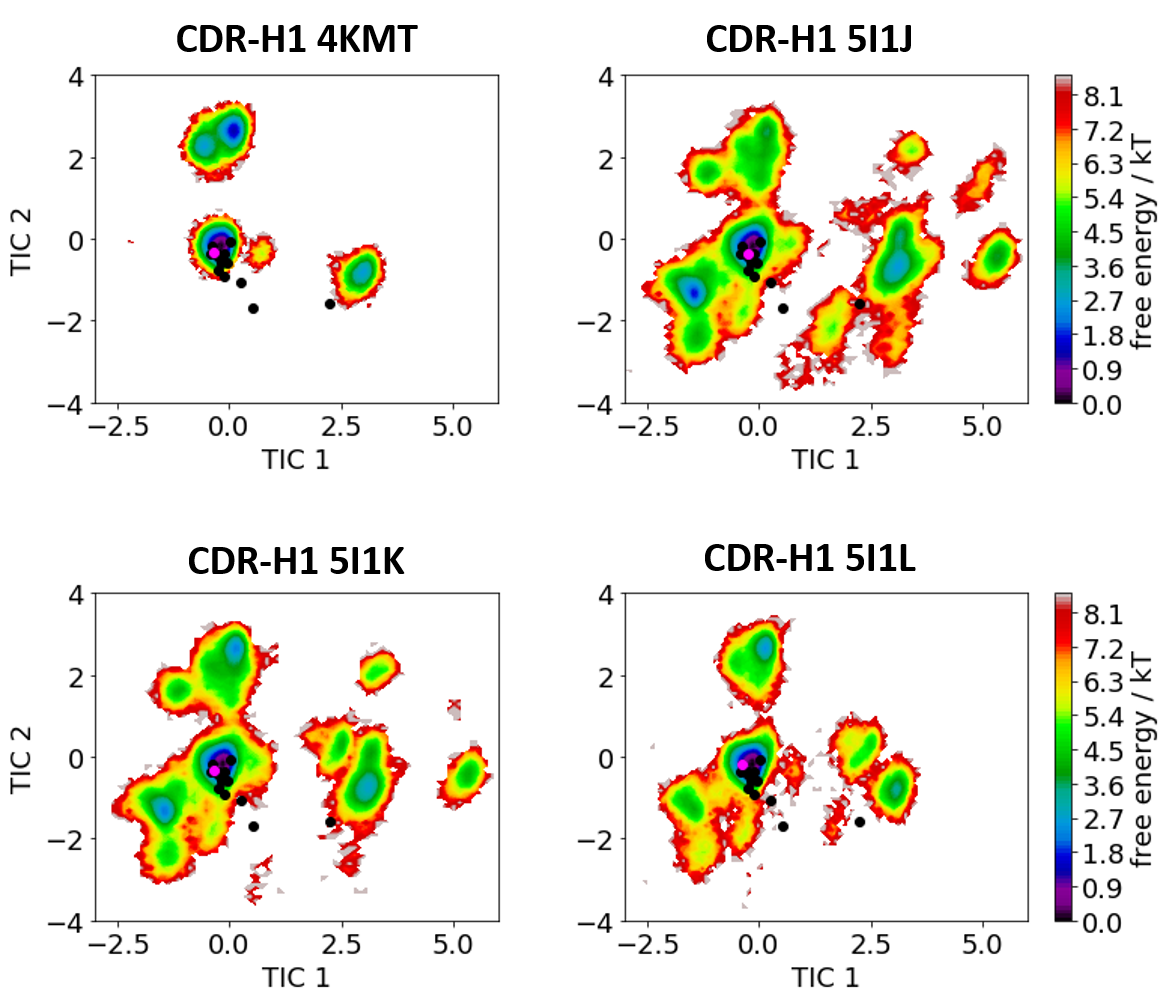
**

SI Figure S7: Comparison of the CDR-H1 loops consisting of the heavy chain germline H5-51. The free energy surface of the CDR-H1 loop paired with different light chain germlines in the same coordinate system is illustrated. The available 16 CDR-H1 loop conformations, resulting from different heavy and light chain pairings, are depicted in black. In pink the respective crystal structures, which were used as starting structures are illustrated (PDB accession codes: 4KMT, 5I1J, 5I1K and 5I1L).

**
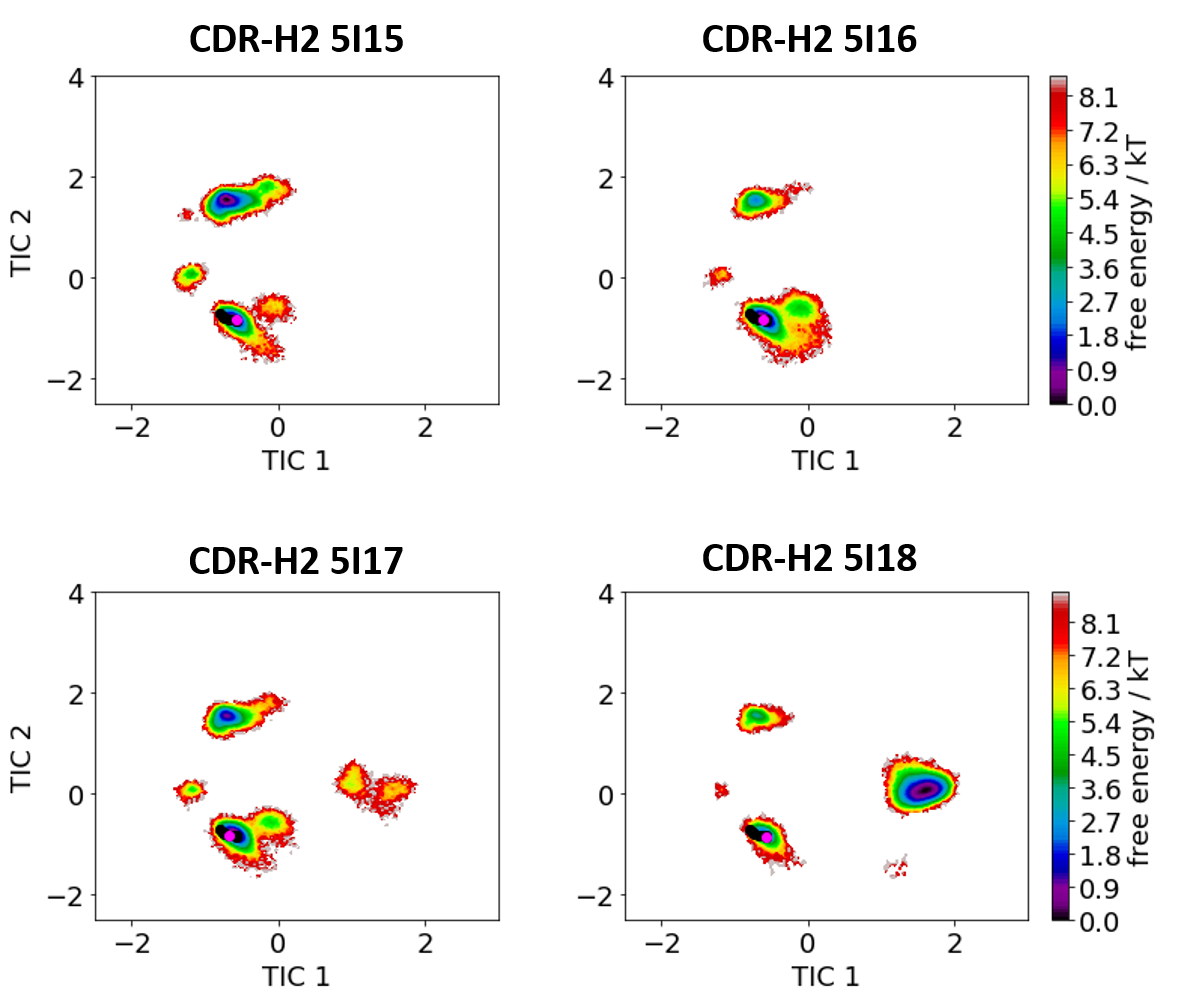
**

SI Figure S8: Comparison of the CDR-H2 loops consisting of the heavy chain germline H1-69. The free energy surface of the CDR-H2 loop paired with different light chain germlines in the same coordinate system is illustrated. The available 16 CDR-H2 loop conformations, resulting from different heavy and light chain pairings, are depicted in black. In pink the respective crystal structures, which were used as starting structures are illustrated (PDB accession codes: 5I15, 5I16, 5I17 and 5I18).

**
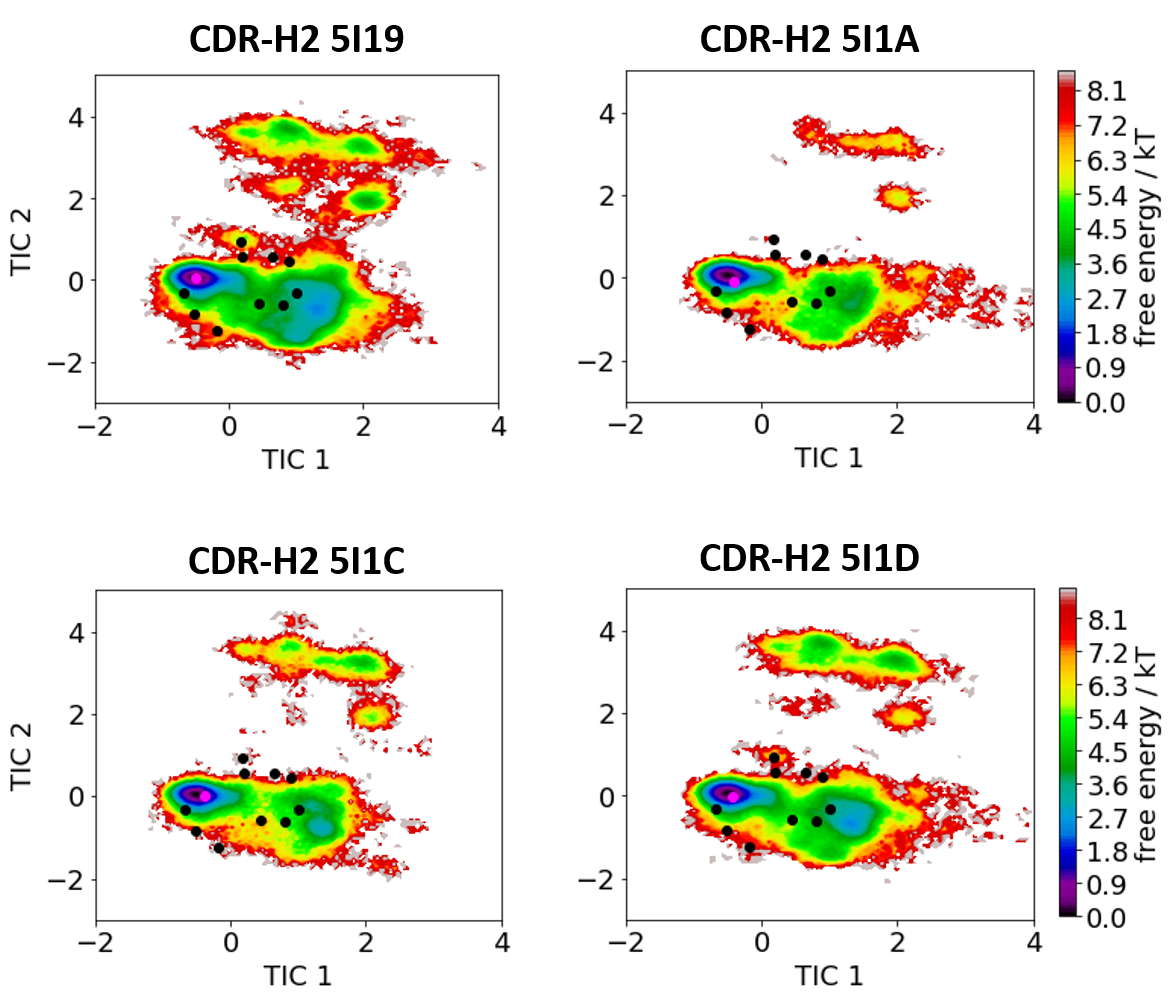
**

SI Figure S9: Comparison of the CDR-H2 loops consisting of the heavy chain germline H1-69. The free energy surface of the CDR-H2 loop paired with different light chain germlines in the same coordinate system is illustrated. The available canonical cluster structure representatives for the CDR-H2 loop of length 10 are projected into the free energy surface and are depicted in black. In pink the respective crystal structures, which were used as starting structures are illustrated (PDB accession codes: 5I19, 5I1A, 5I1C and 5I1D).

**
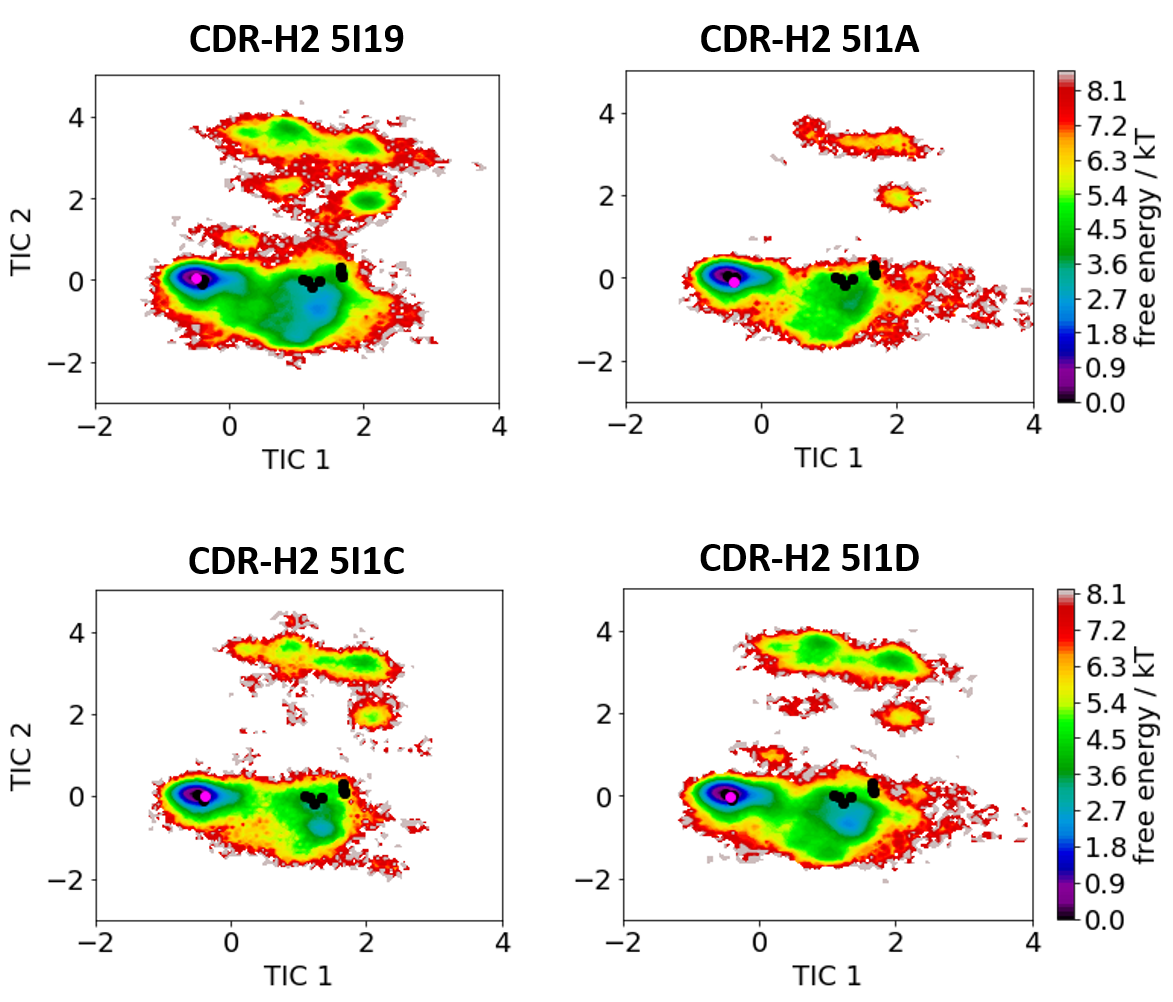
**

SI Figure S10: Comparison of the CDR-H2 loops consisting of the heavy chain germline H3-23. The free energy surface of the CDR-H2 loop paired with different light chain germlines in the same coordinate system is illustrated. The available 16 CDR-H2 loop conformations, resulting from different heavy and light chain pairings, are depicted in black. In pink the respective crystal structures, which were used as starting structures are illustrated (PDB accession codes: 5I19, 5I1A, 5I1C and 5I1D).

**
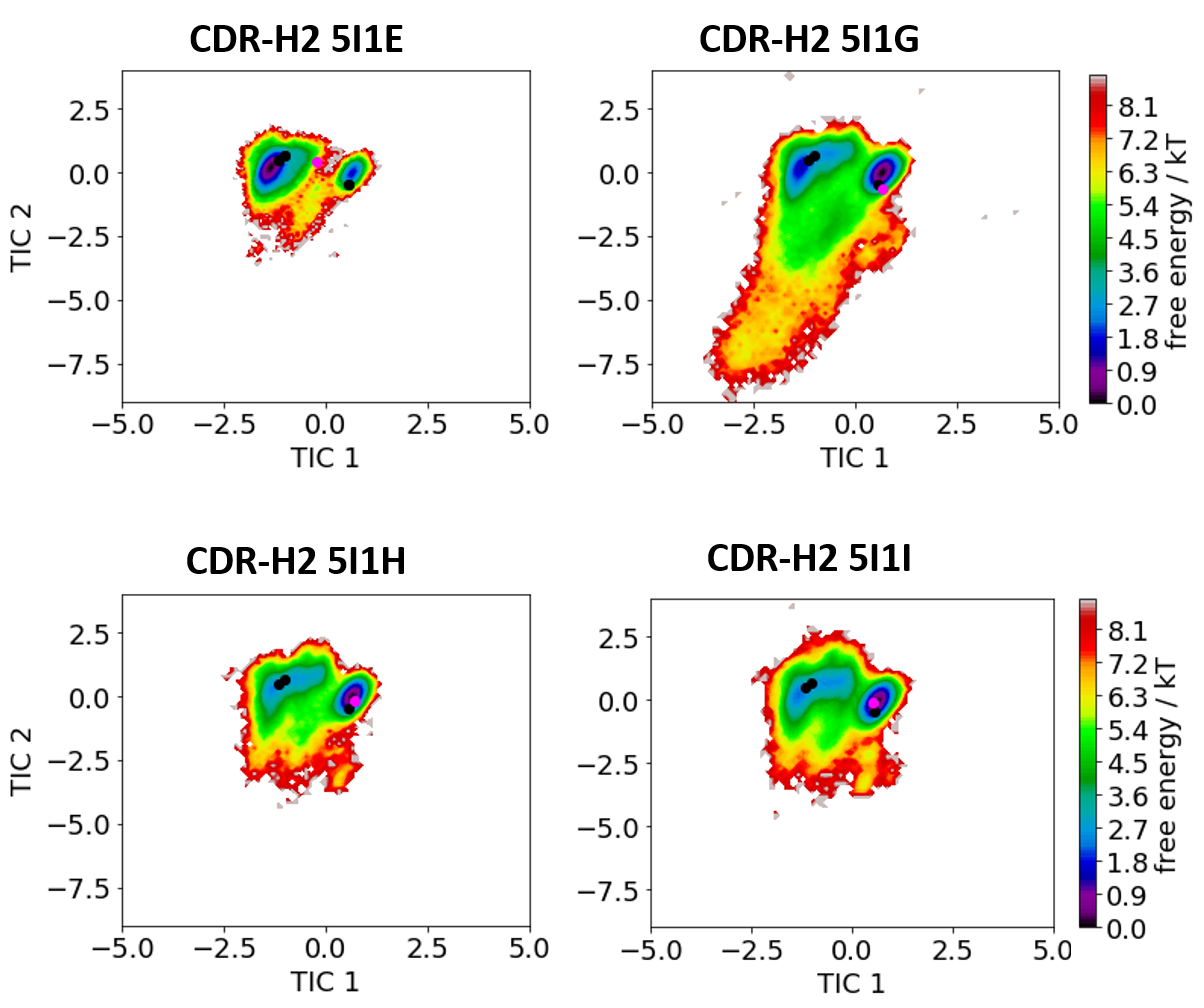
**

SI Figure S11: Comparison of the CDR-H2 loops consisting of the heavy chain germline H3-53. The free energy surface of the CDR-H2 loop paired with different light chain germlines in the same coordinate system is illustrated. The available canonical cluster structure representatives for the CDR-H2 loop of length 9 are projected into the free energy surface and are depicted in black. In pink the respective crystal structures, which were used as starting structures are illustrated (PDB accession codes: 5I1E, 5I1G, 5I1H and 5I1I)

**
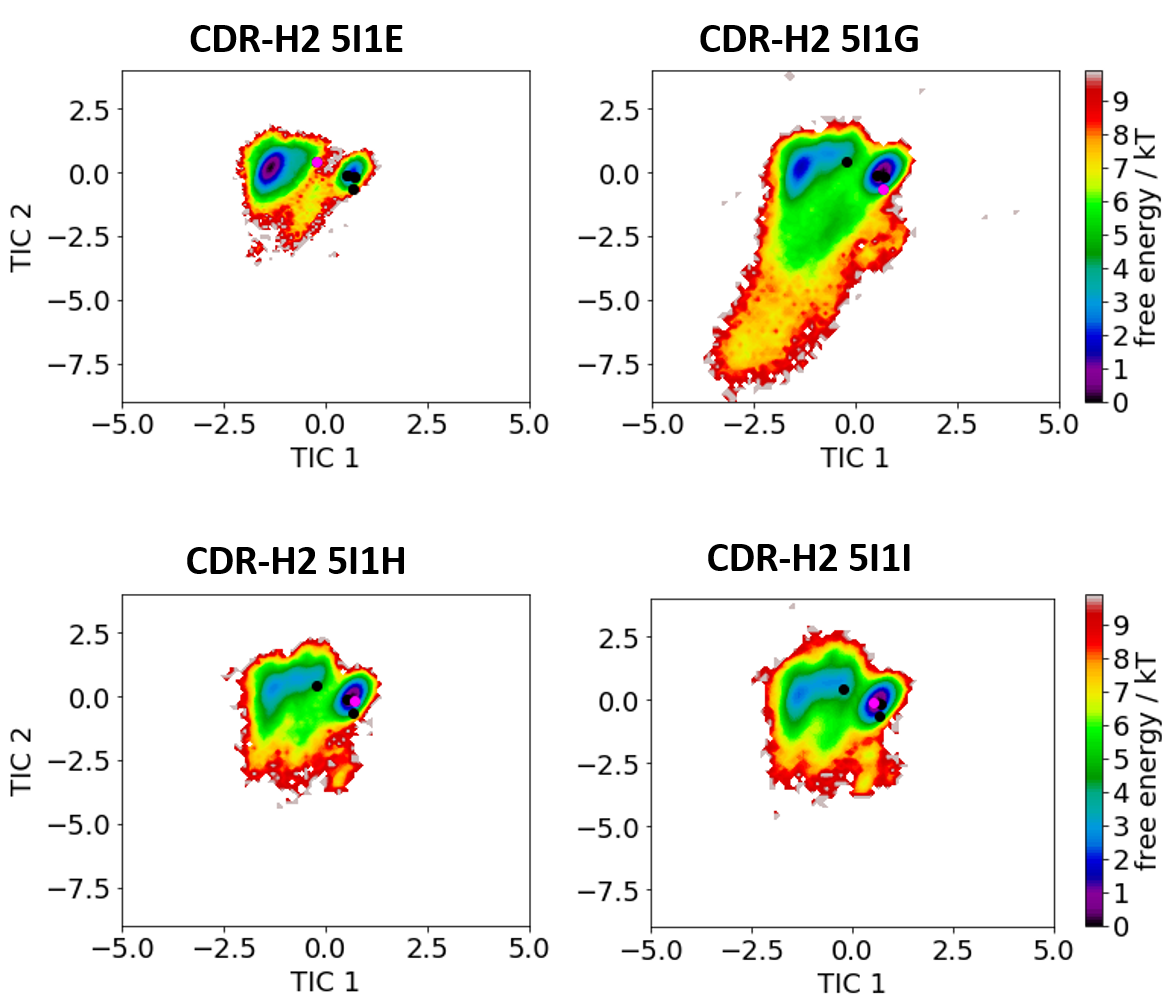
**

SI Figure S12: Comparison of the CDR-H2 loops consisting of the heavy chain germline H3-53. The free 5energy surface of the CDR-H2 loop paired with different light chain germlines in the same coordinate system is illustrated. The available 4 CDR-H2 loop conformations, resulting from different heavy and light chain pairings, are depicted in black. In pink the respective crystal structures, which were used as starting structures are illustrated (PDB accession codes: 5I1E, 5I1G, 5I1H and 5I1I).

**
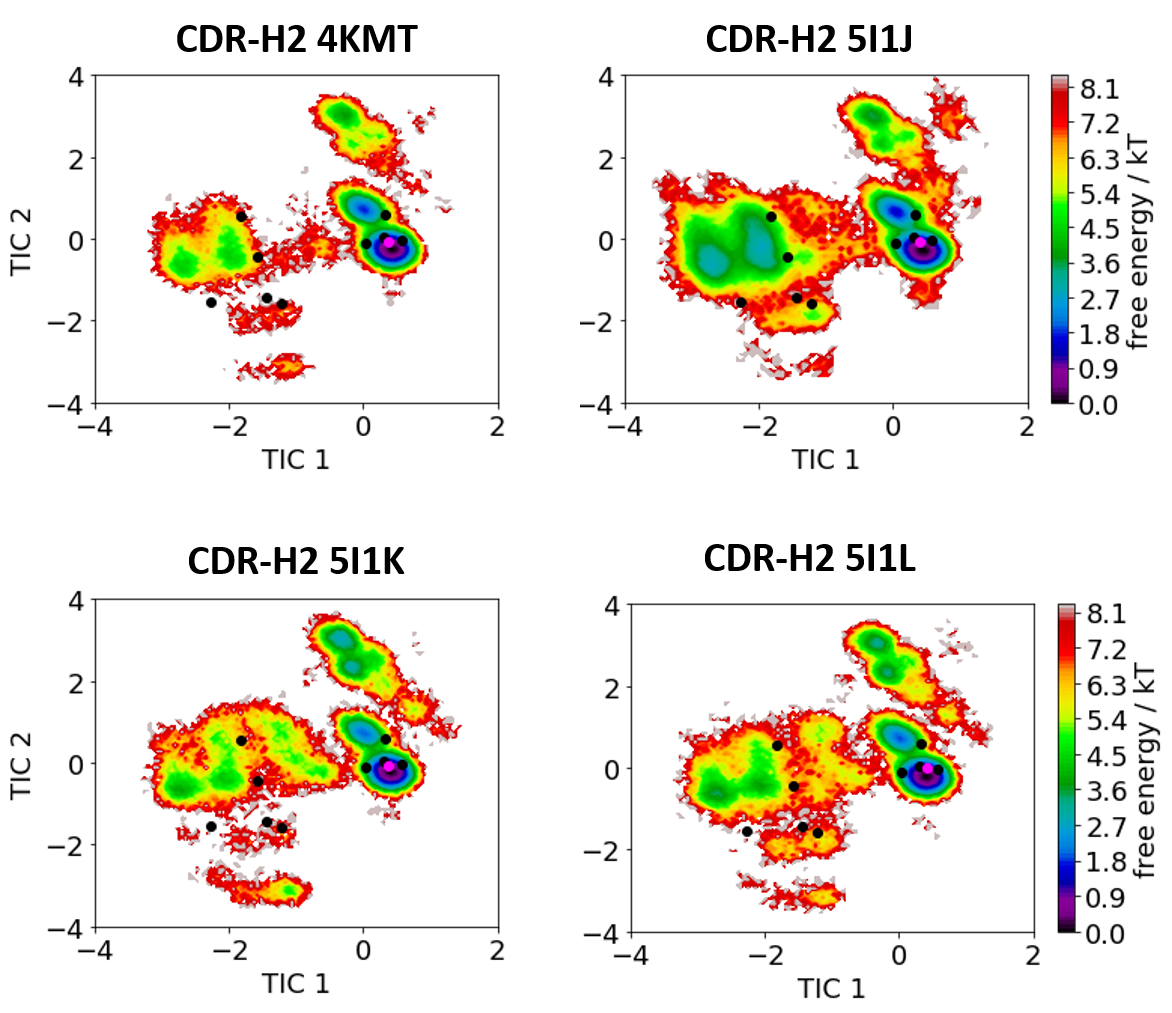
**

SI Figure S13: Comparison of the CDR-H2 loops consisting of the heavy chain germline H5-51. The free energy surface of the CDR-H2 loop paired with different light chain germlines in the same coordinate system is illustrated. The available canonical cluster structure representatives for the CDR-H2 loop of length 10 are projected into the free energy surface and are depicted in black. In pink the respective crystal structures, which were used as starting structures are illustrated (PDB accession codes: 4KMT, 5I1J, 5I1K and 5I1L).

**
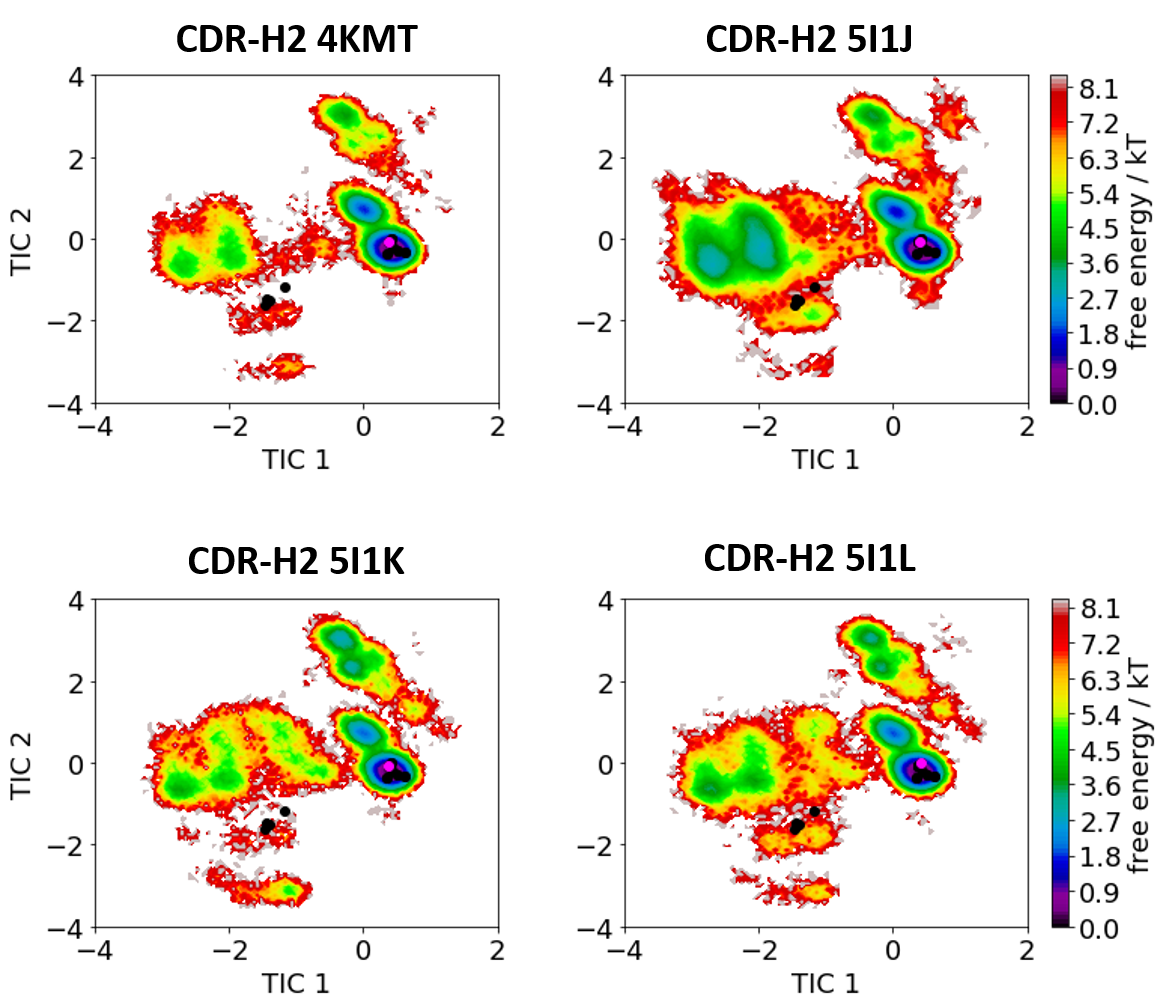
**

SI Figure S14: Comparison of the CDR-H2 loops consisting of the heavy chain germline H5-51. The free 5energy surface of the CDR-H2 loop paired with different light chain germlines in the same coordinate system is illustrated. The available 16 CDR-H2 loop conformations, resulting from different heavy and light chain pairings, are depicted in black. In pink the respective crystal structures, which were used as starting structures are illustrated (PDB accession codes: 4KMT, 5I1J, 5I1K and 5I1L).

**
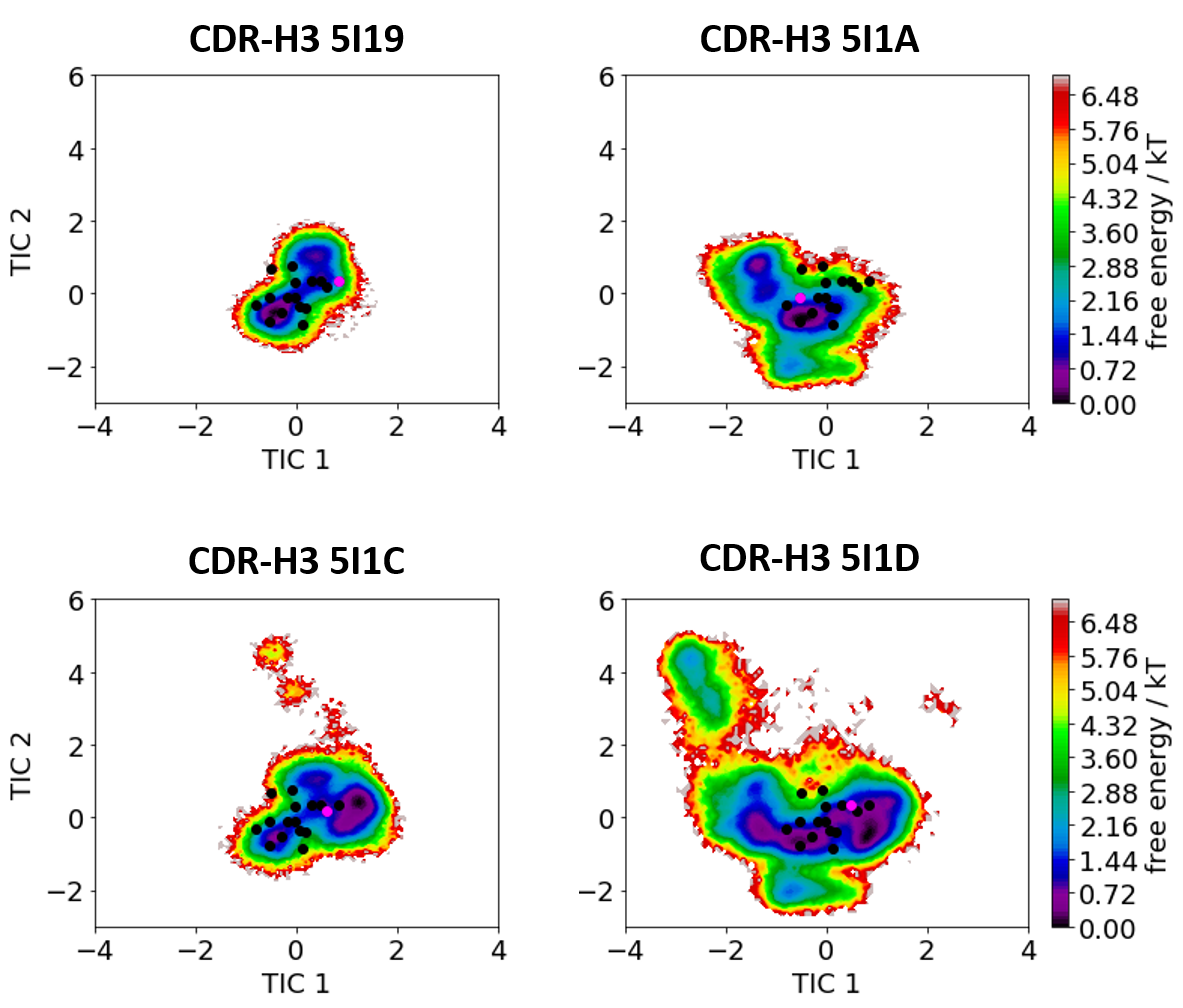
**

SI Figure S15: Comparison of the CDR-H3 loops consisting of the heavy chain germline H3-23. The free energy surface of the CDR-H3 loop paired with different light chain germlines in the same coordinate system is illustrated. The available 16 CDR-H3 loop conformations, resulting from different heavy and light chain pairings, are depicted in black. In pink the respective crystal structures, which were used as starting structures are illustrated (PDB accession codes: 5I19, 5I1A, 5I1C and 5I1D).

**
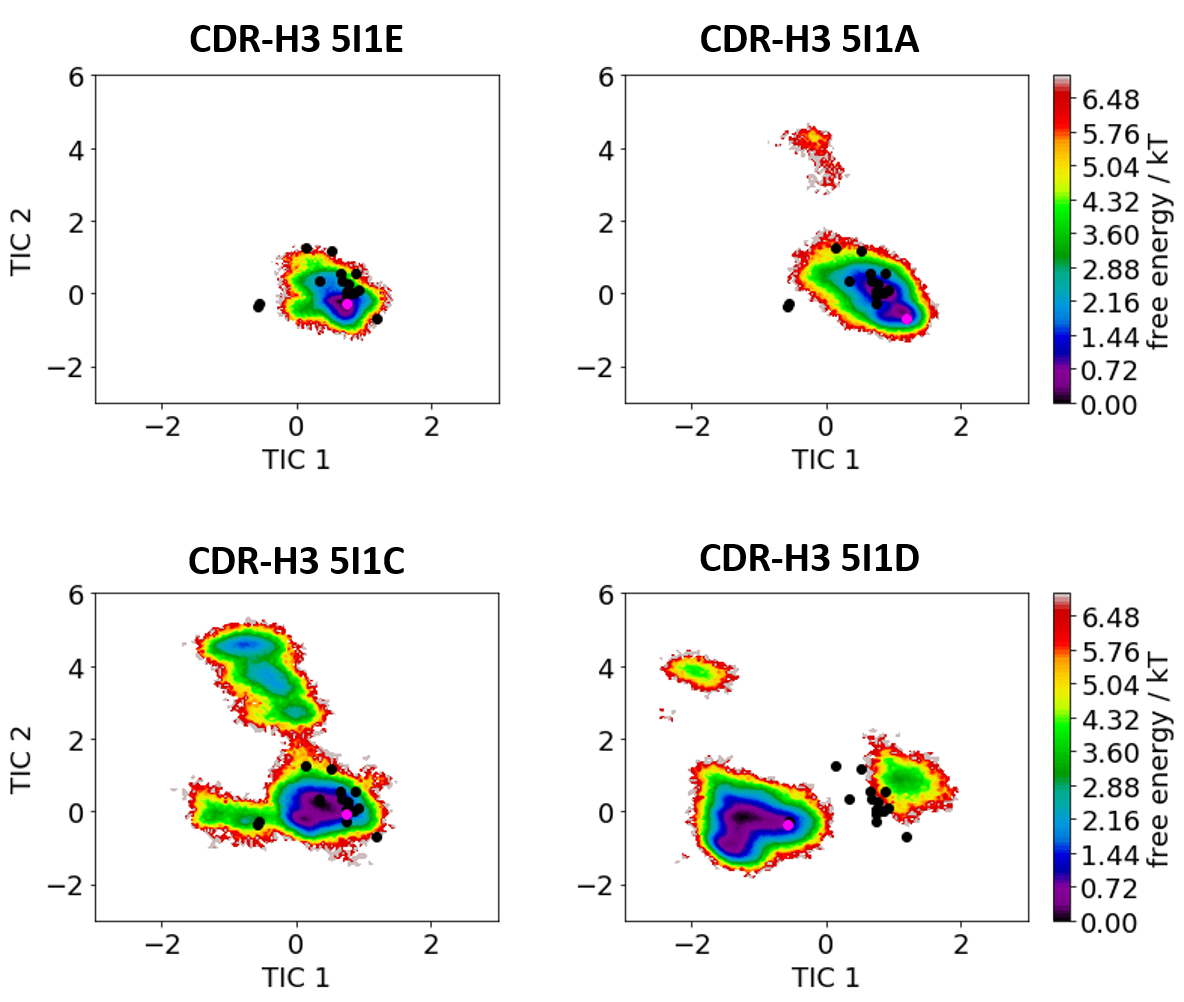
**

SI Figure S16: Comparison of the CDR-H3 loops consisting of the heavy chain germline H3-53. The free energy surface of the CDR-H3 loop paired with different light chain germlines in the same coordinate system is illustrated. The available 16 CDR-H3 loop conformations, resulting from different heavy and light chain pairings, are depicted in black. In pink the respective crystal structures, which were used as starting structures are illustrated (PDB accession codes: 5I1E, 5I1A, 5I1C, 5I1D).

**
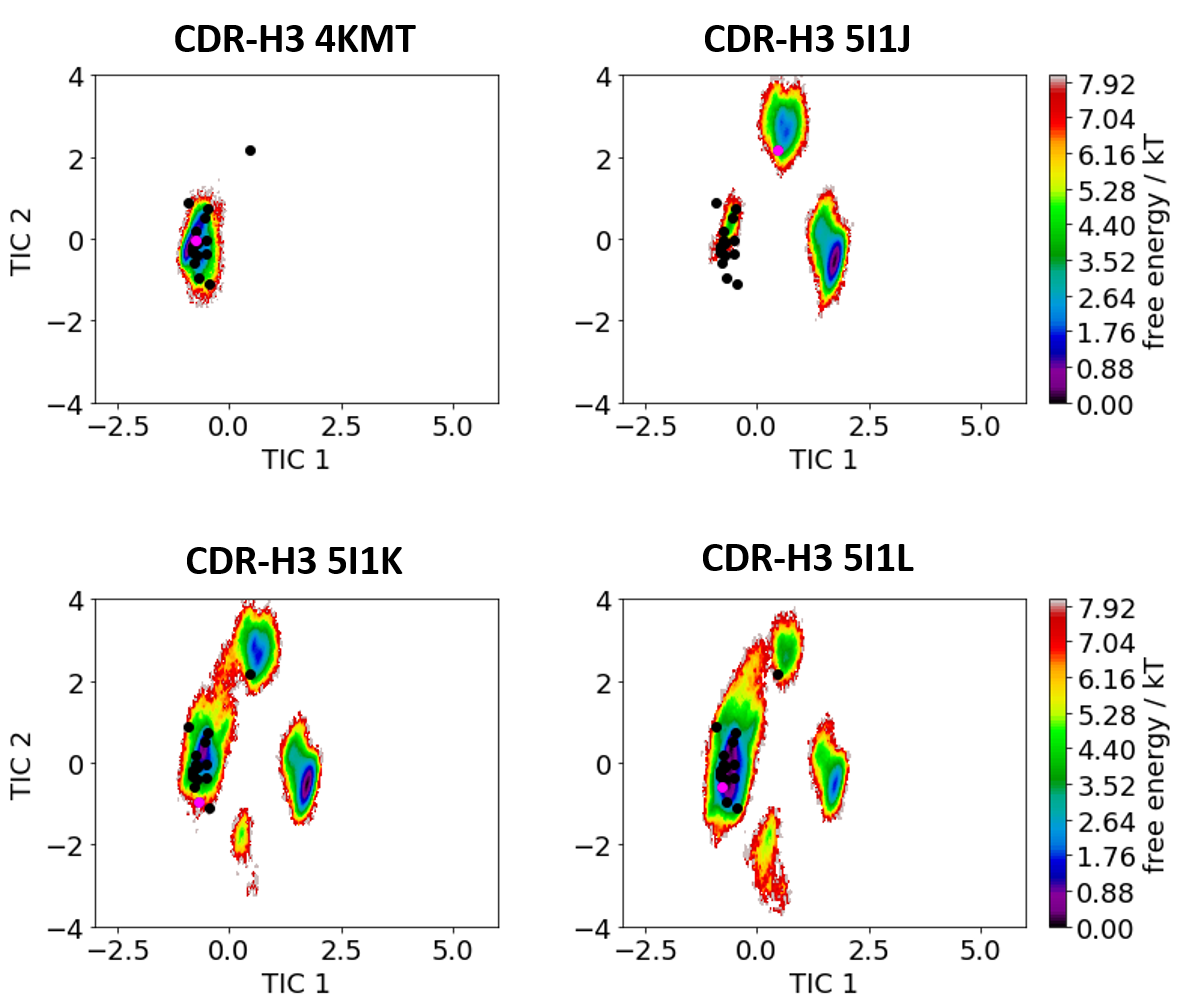
**

SI Figure S17: Comparison of the CDR-H3 loops consisting of the heavy chain germline H5-51. The free energy surface of the CDR-H3 loop paired with different light chain germlines in the same coordinate system is illustrated. The available 16 CDR-H3 loop conformations, resulting from different heavy and light chain pairings, are depicted in black. In pink the respective crystal structures, which were used as starting structures are illustrated (PDB accession codes: 4KMT, 5I1J, 5I1K and 5I1L).

**
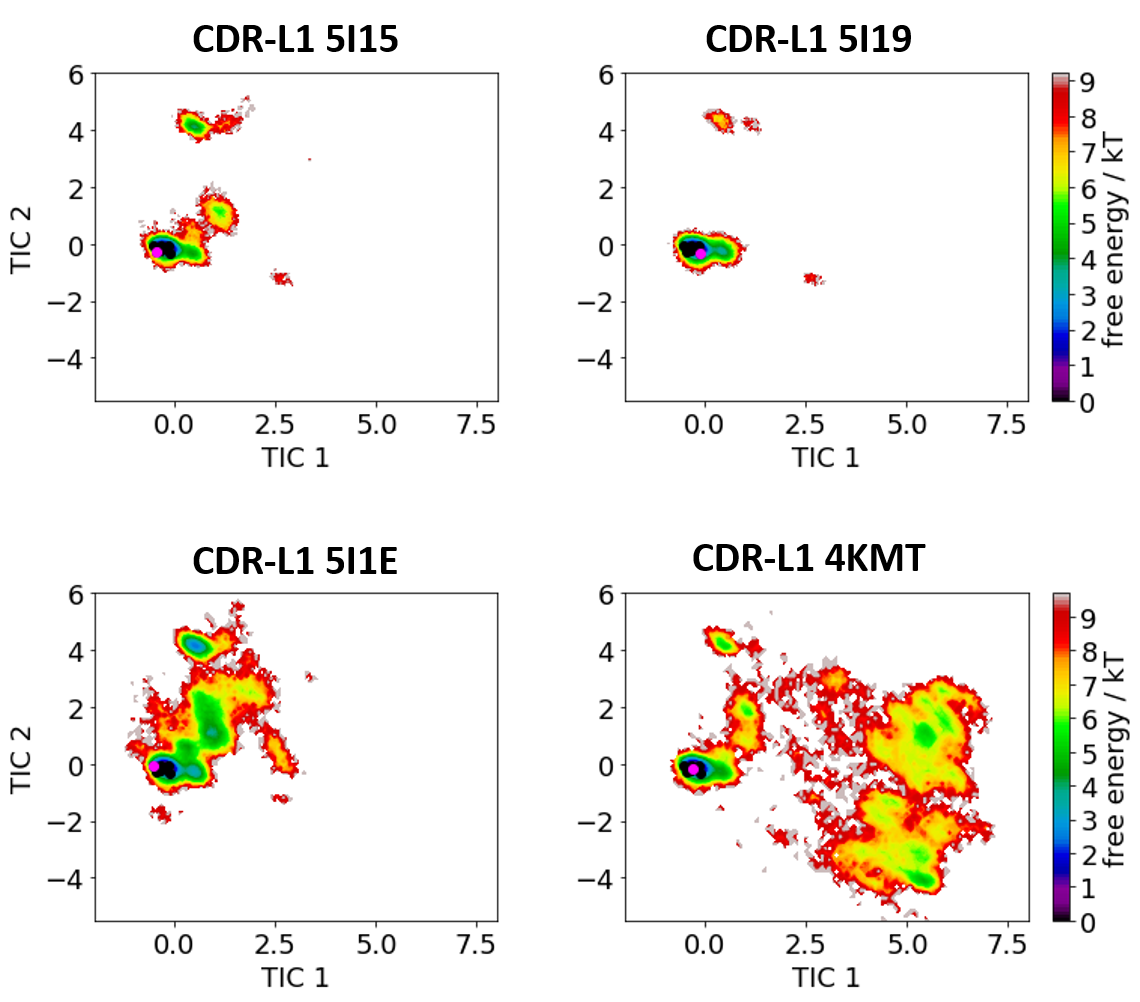
**

SI Figure S18: Comparison of the CDR-L1 loops consisting of the heavy chain germline L1-39. The free energy surface of the CDR-L1 loop paired with different light chain germlines in the same coordinate system is illustrated. The available 8 CDR-L1 loop conformations, resulting from different heavy and light chain pairings, are depicted in black. In pink the respective crystal structures, which were used as starting structures are illustrated (PDB accession codes: 5I15, 5I19, 5I1E and 4KMT).

**
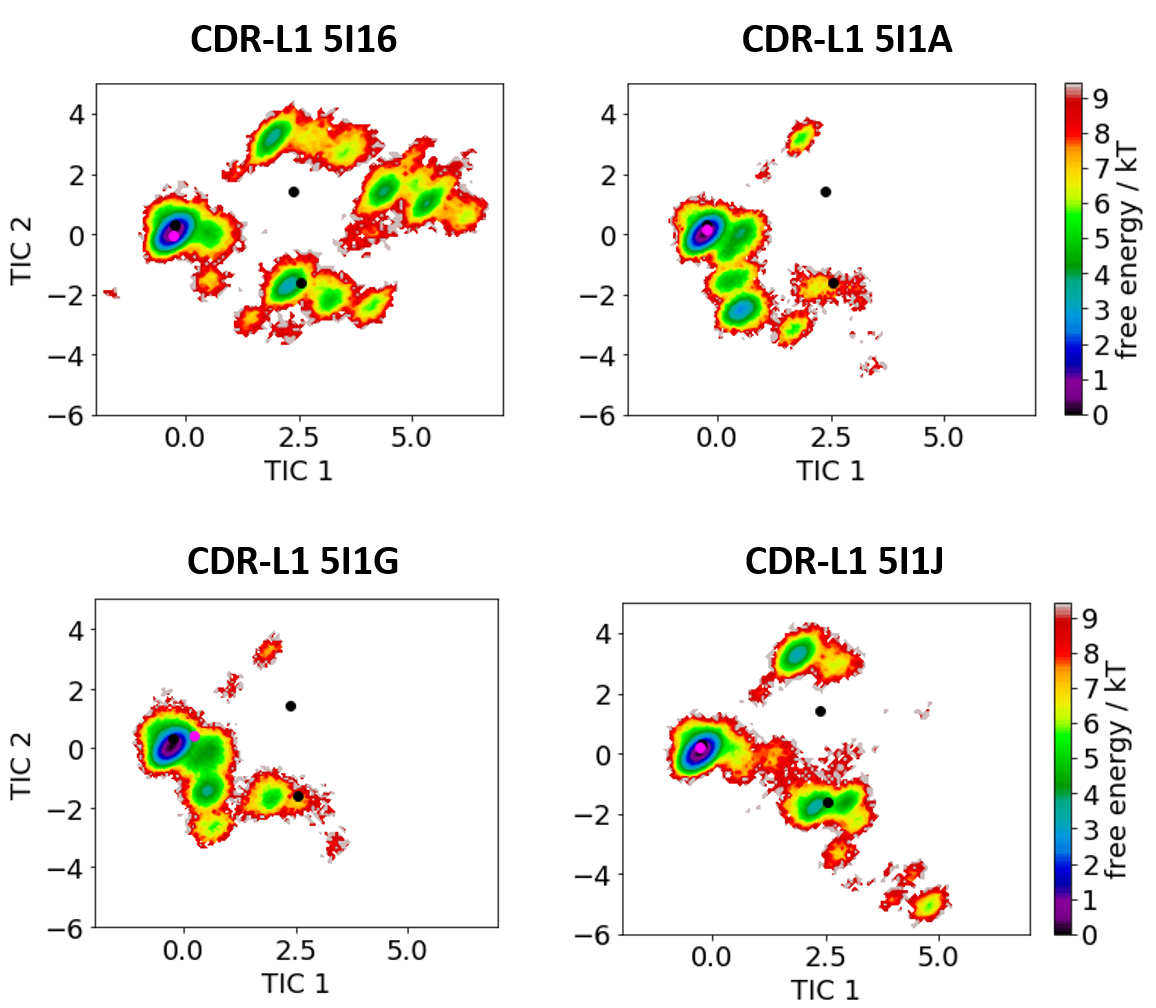
**

SI Figure S19: Comparison of the CDR-L1 loops consisting of the heavy chain germline L3-11. The free energy surface of the CDR-L1 loop paired with different light chain germlines in the same coordinate system is illustrated. The available canonical cluster structure representatives for the CDR-L1 loop of length 11 are projected into the free energy surface and are depicted in black. In pink the respective crystal structures, which were used as starting structures are illustrated (PDB accession codes: 5I16, 5I1A, 5I1G and 5I1J).

**
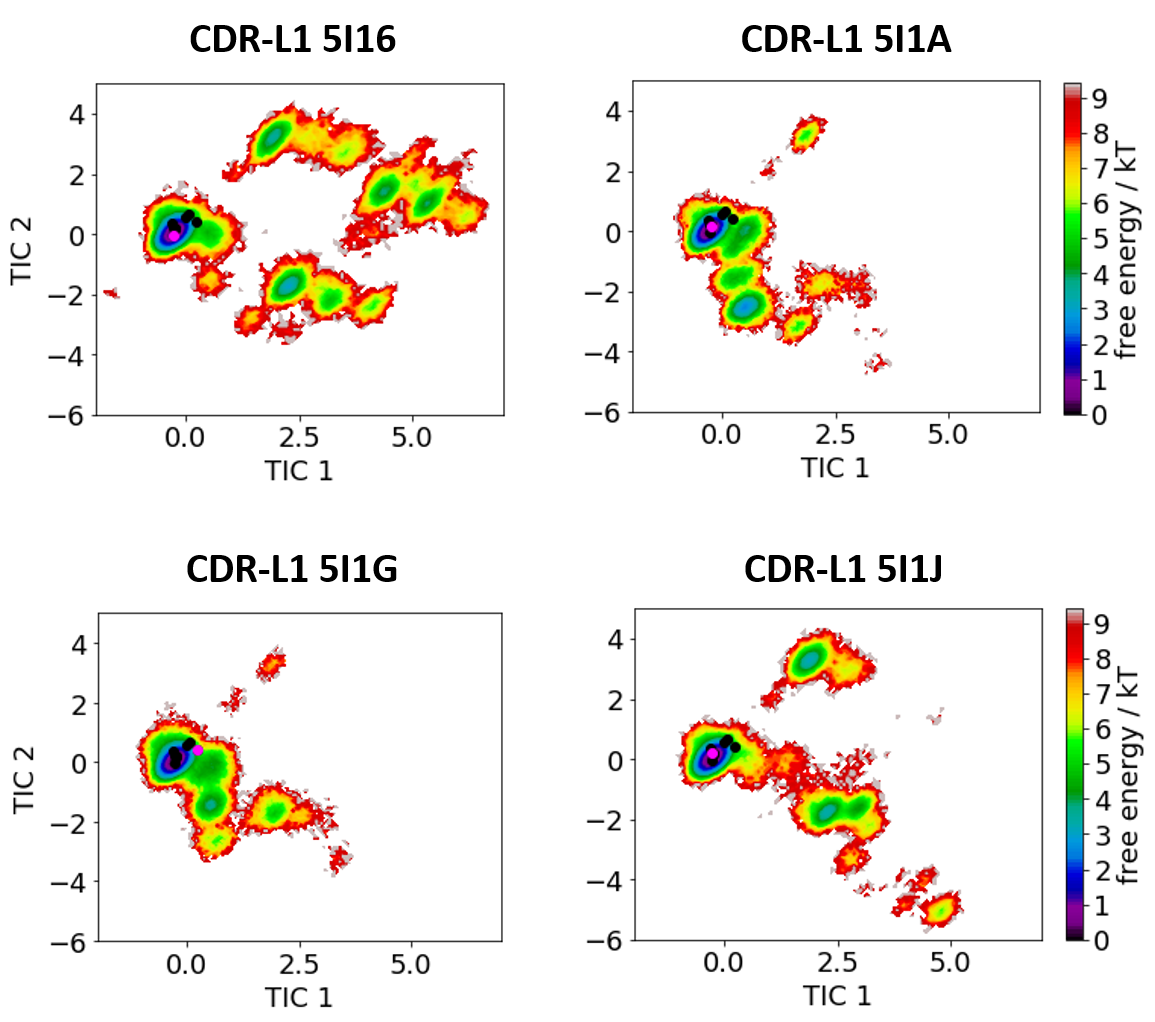
**

SI Figure S20: Comparison of the CDR-L1 loops consisting of the heavy chain germline L3-11. The free energy surface of the CDR-L1 loop paired with different light chain germlines in the same coordinate system is illustrated. The available 8 CDR-L1 loop conformations, resulting from different heavy and light chain pairings, are depicted in black. In pink the respective crystal structures, which were used as starting structures are illustrated (PDB accession codes: 5I16, 5I1A, 5I1G and 5I1J).

**
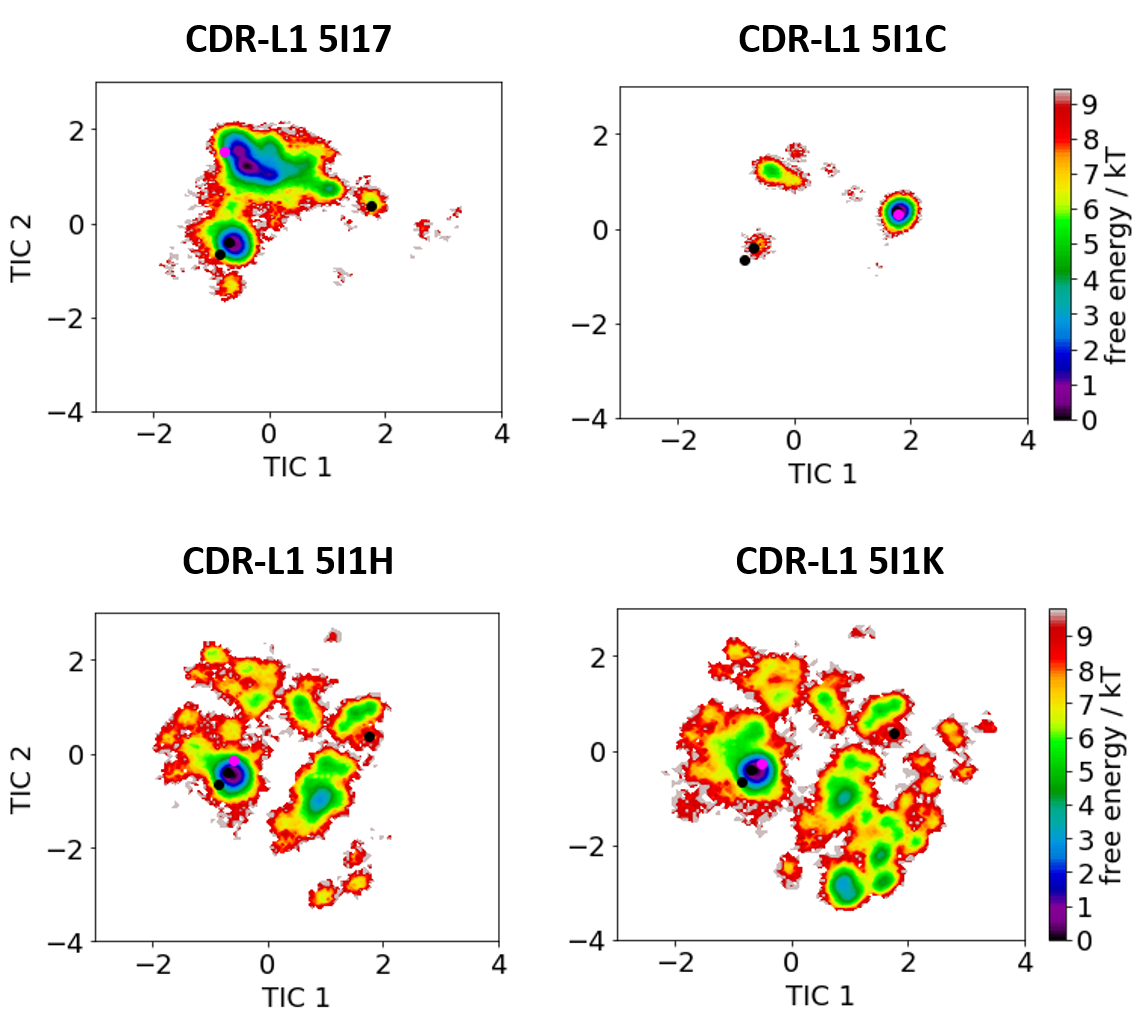
**

SI Figure S21: Comparison of the CDR-L1 loops consisting of the heavy chain germline L3-20. The free energy surface of the CDR-L1 loop paired with different light chain germlines in the same coordinate system is illustrated. The available canonical cluster structure representatives for the CDR-L1 loop of length 12 are projected into the free energy surface and are depicted in black. In pink the respective crystal structures, which were used as starting structures are illustrated (PDB accession codes: 5I17, 5I1C, 5I1H and 5I1K).

**
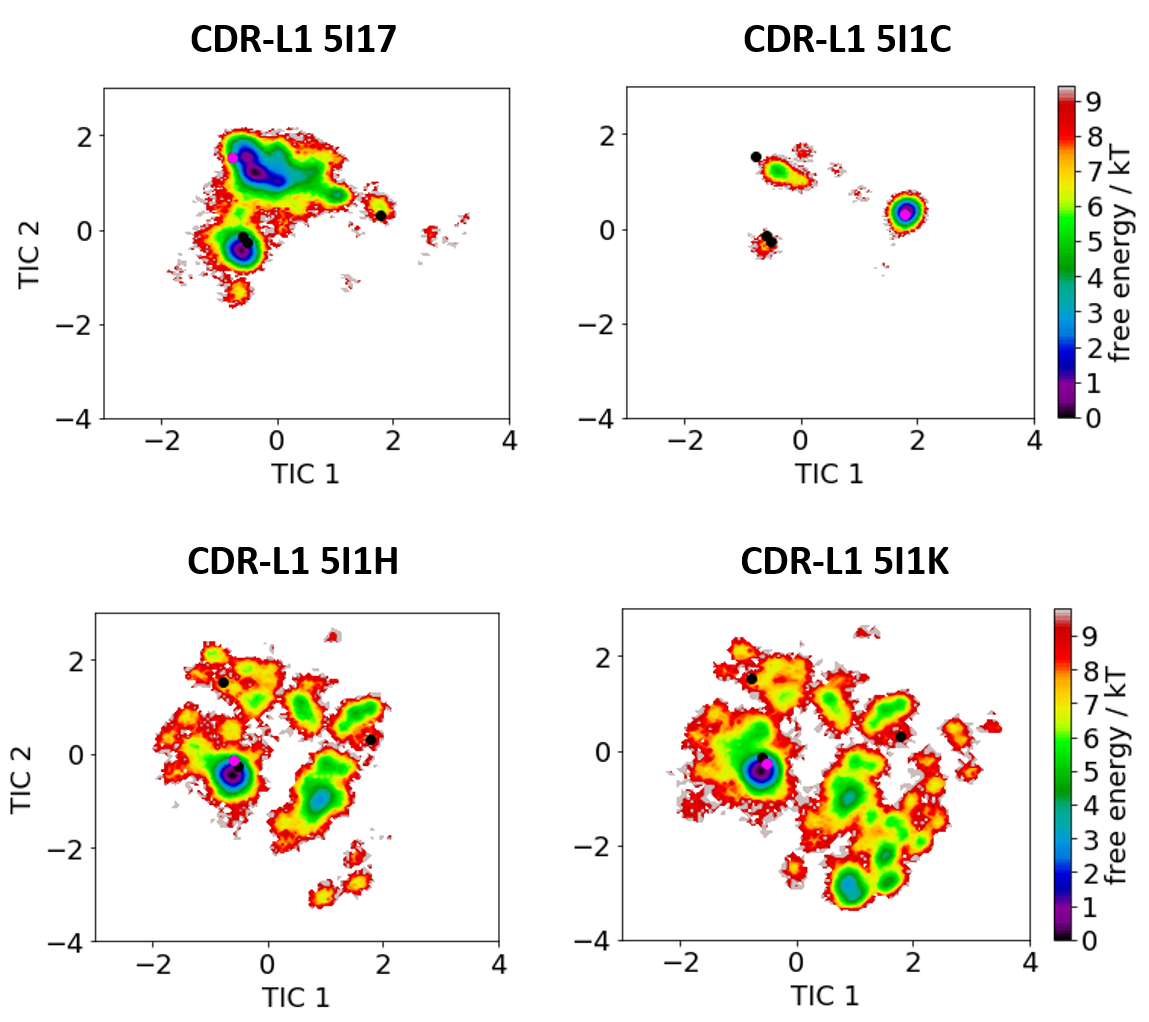
**

SI Figure S22: Comparison of the CDR-L1 loops consisting of the heavy chain germline L3-20. The free energy surface of the CDR-L1 loop paired with different light chain germlines in the same coordinate system is illustrated. The available 4 CDR-L1 loop conformations, resulting from different heavy and light chain pairings, are depicted in black. In pink the respective crystal structures, which were used as starting structures are illustrated (PDB accession codes: 5I17, 5I1C, 5I1H and 5I1K).

**
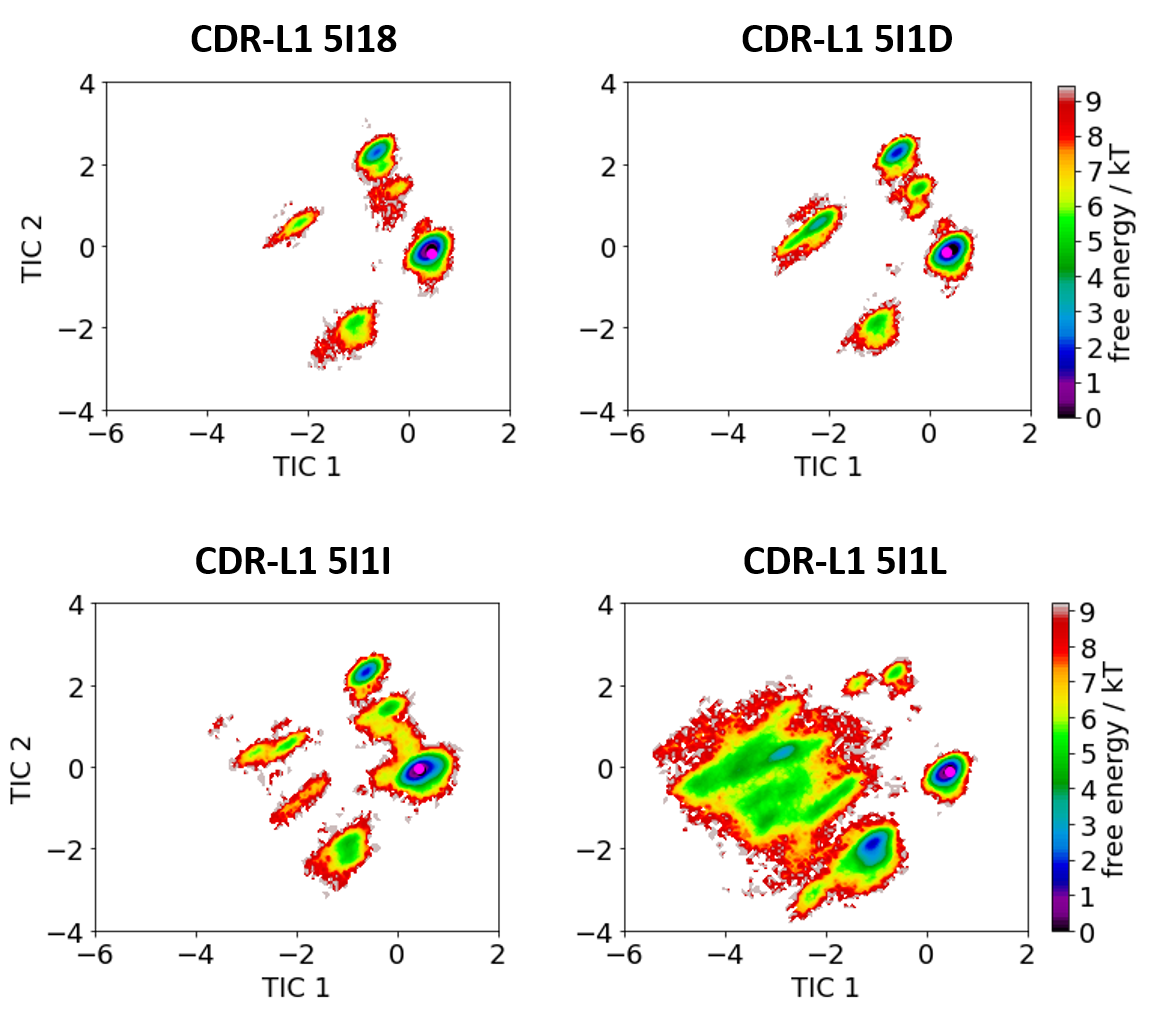
**

SI Figure S23: Comparison of the CDR-L1 loops consisting of the heavy chain germline L4-1. The free energy surface of the CDR-L1 loop paired with different light chain germlines in the same coordinate system is illustrated. The available canonical cluster structure representative for the CDR-L1 loop of length 17 is projected into the free energy surface and is depicted in black. In pink the respective crystal structures, which were used as starting structures are illustrated (PDB accession codes: 5I18, 5I1D, 5I1I and 5I1L).

**
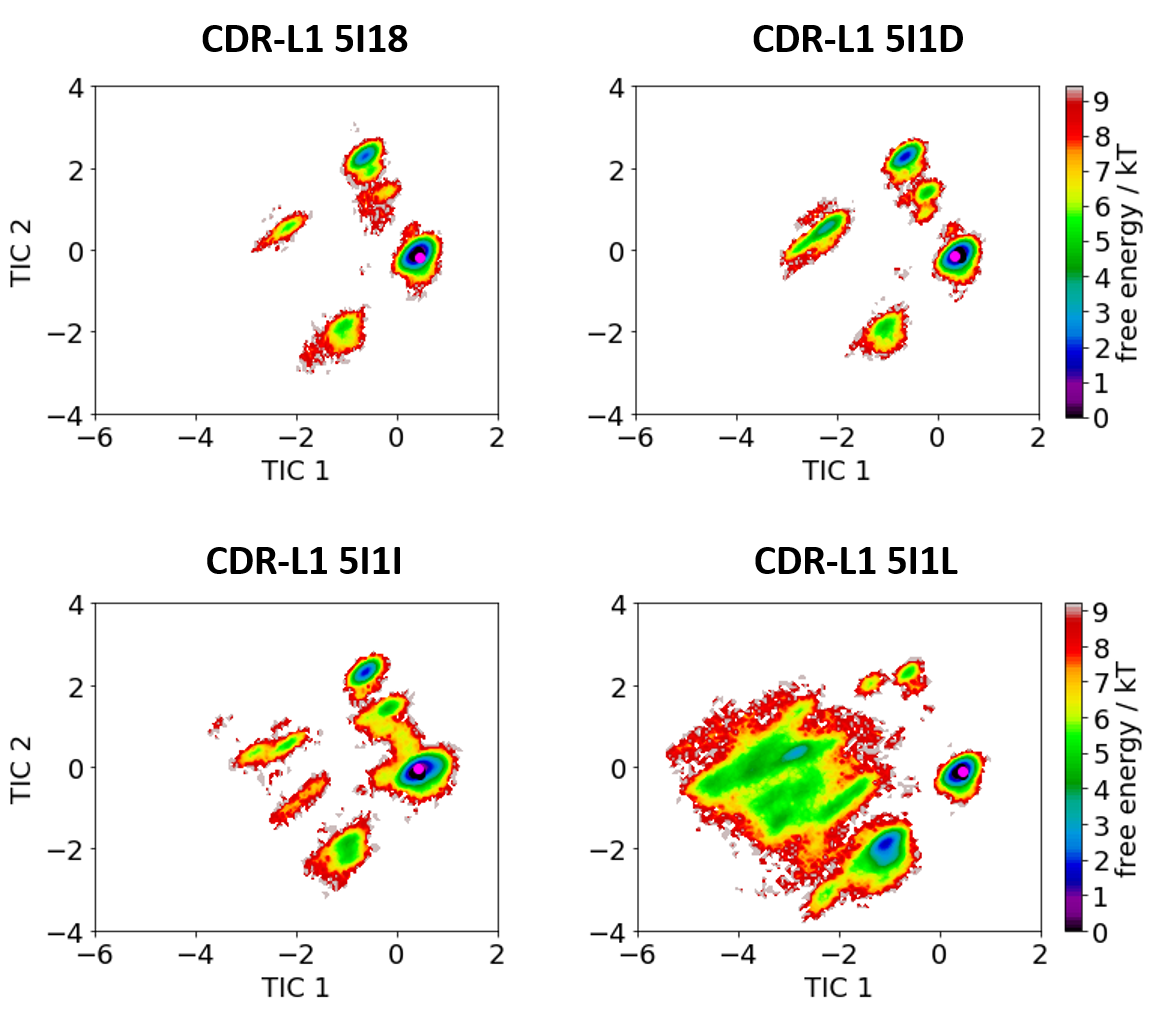
**

SI Figure S24: Comparison of the CDR-L1 loops consisting of the heavy chain germline L4-1. The free energy surface of the CDR-L1 loop paired with different light chain germlines in the same coordinate system is illustrated. The available 4 CDR-L1 loop conformations, resulting from different heavy and light chain pairings, are depicted in black. In pink the respective crystal structures, which were used as starting structures are illustrated (PDB accession codes: 5I18, 5I1D, 5I1I and 5I1L).

**
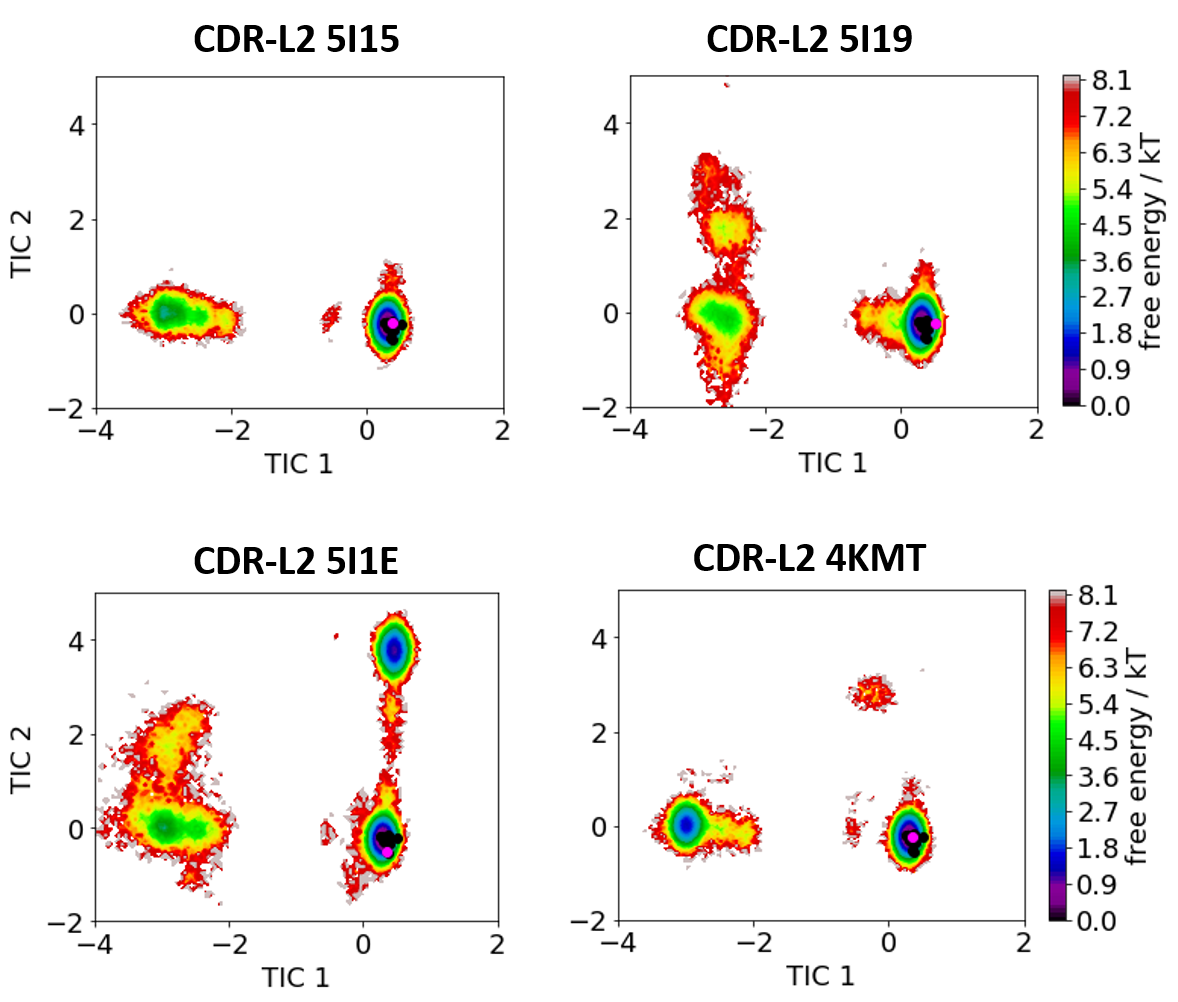
**

SI Figure S25: Comparison of the CDR-L2 loops consisting of the heavy chain germline L1-39. The free energy surface of the CDR-L2 loop paired with different light chain germlines in the same coordinate system is illustrated. The available 16 CDR-L2 loop conformations, resulting from different heavy and light chain pairings, are depicted in black. In pink the respective crystal structures, which were used as starting structures are illustrated (PDB accession codes: 5I15, 5I19, 5I1E and 4KMT).

**
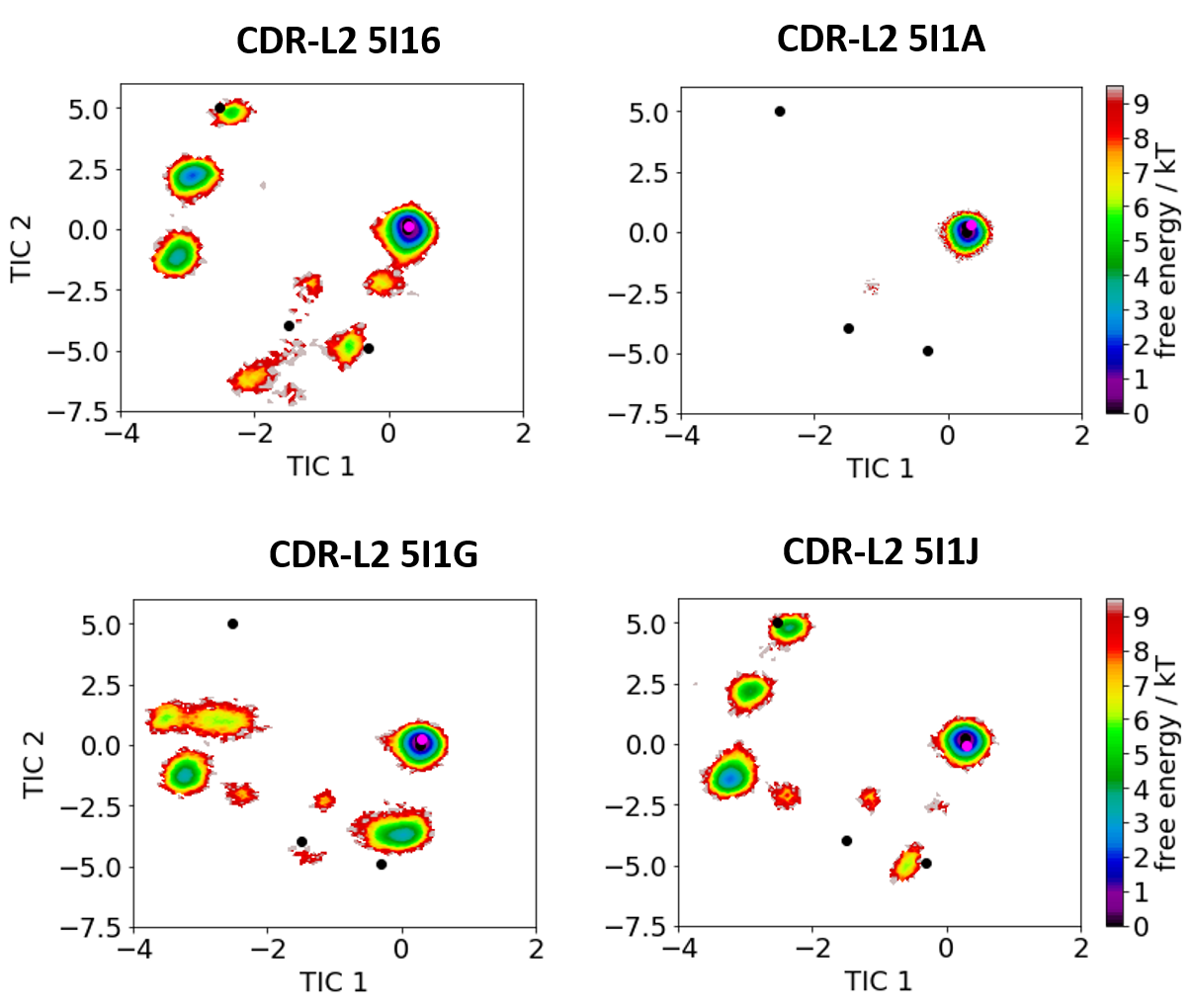
**

SI Figure S26: Comparison of the CDR-L2 loops consisting of the heavy chain germline L3-11. The free energy surface of the CDR-L2 loop paired with different light chain germlines in the same coordinate system is illustrated. The available canonical cluster structure representative for the CDR-L2 loop of length 8 is projected into the free energy surface and is depicted in black. In pink the respective crystal structures, which were used as starting structures are illustrated (PDB accession codes: 5I16, 5I1A, 5I1G and 5I1J).

**
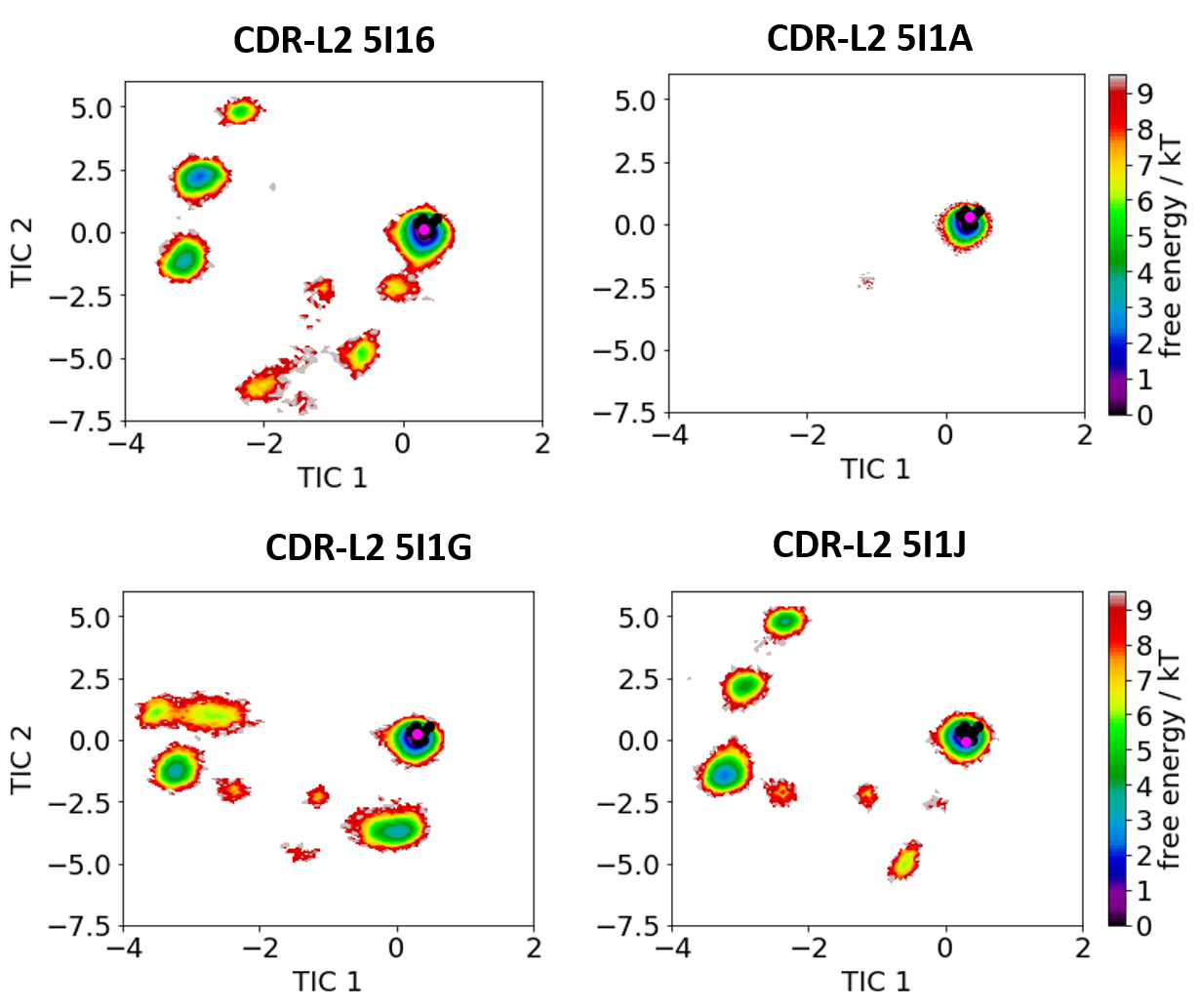
**

SI Figure S27: Comparison of the CDR-L2 loops consisting of the heavy chain germline L3-11. The free energy surface of the CDR-L2 loop paired with different light chain germlines in the same coordinate system is illustrated. The available 16 CDR-L2 loop conformations, resulting from different heavy and light chain pairings, are depicted in black. In pink the respective crystal structures, which were used as starting structures are illustrated (PDB accession codes: 5I16, 5I1A, 5I1G and 5I1J).

**
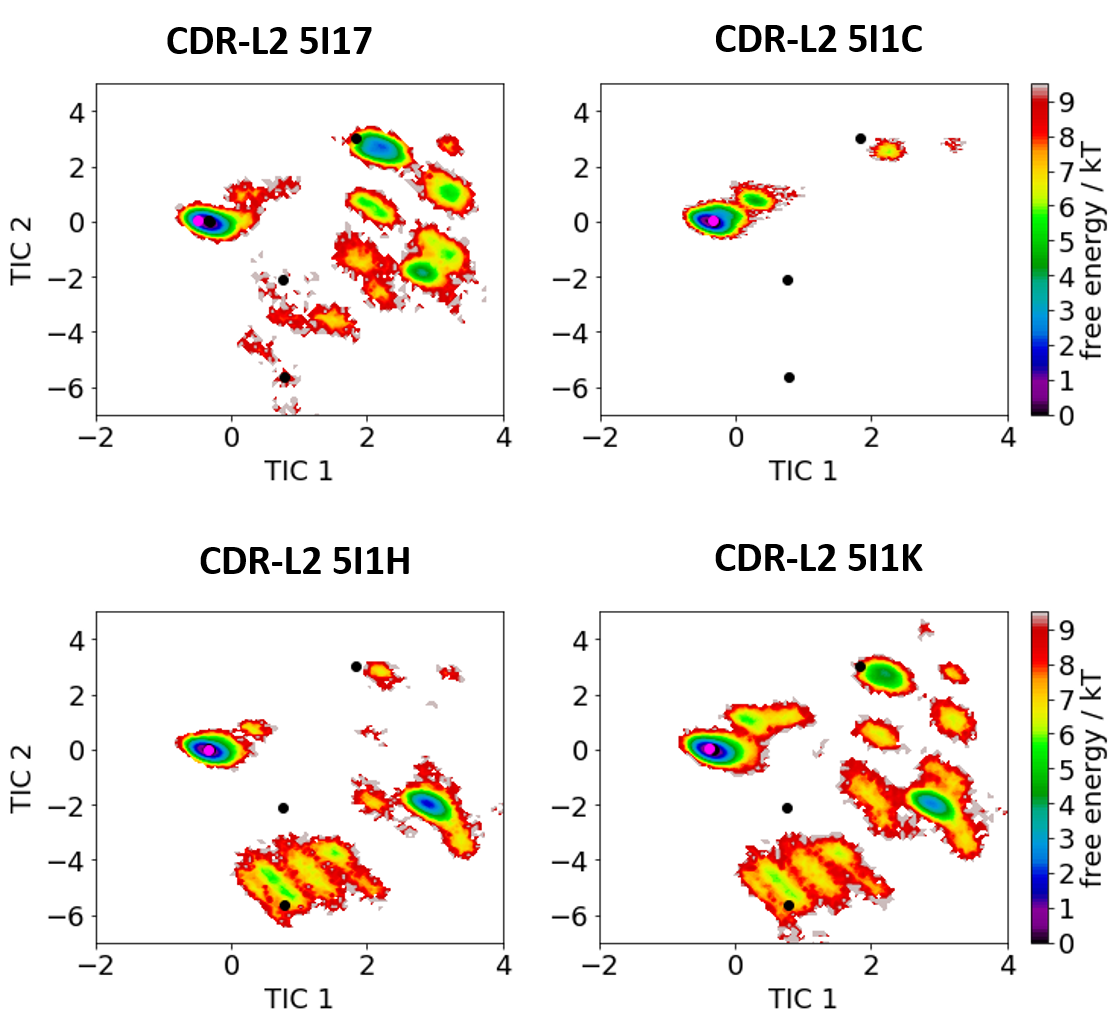
**

SI Figure S28: Comparison of the CDR-L2 loops consisting of the heavy chain germline L3-20. The free energy surface of the CDR-L2 loop paired with different light chain germlines in the same coordinate system is illustrated. The available canonical cluster structure representative for the CDR-L2 loop of length 8 is projected into the free energy surface and is depicted in black. In pink the respective crystal structures, which were used as starting structures are illustrated (PDB accession codes: 5I17, 5I1C, 5I1H and 5I1K).

**
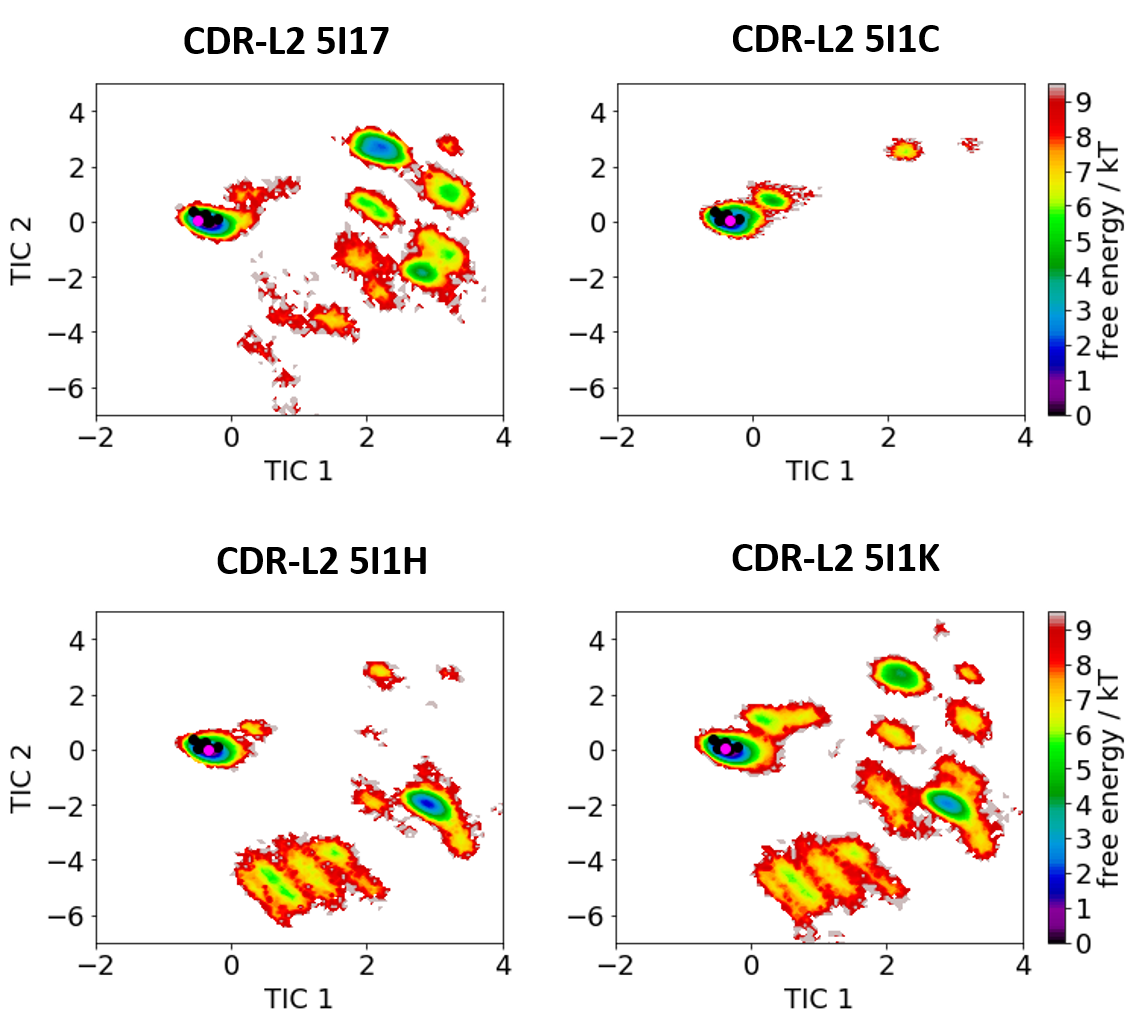
**

SI Figure S29: Comparison of the CDR-L2 loops consisting of the heavy chain germline L3-20. The free energy surface of the CDR-L2 loop paired with different light chain germlines in the same coordinate system is illustrated. The available 16 CDR-L2 loop conformations, resulting from different heavy and light chain pairings, are depicted in black. In pink the respective crystal structures, which were used as starting structures are illustrated (PDB accession codes: 5I17, 5I1C, 5I1H and 5I1K).

**
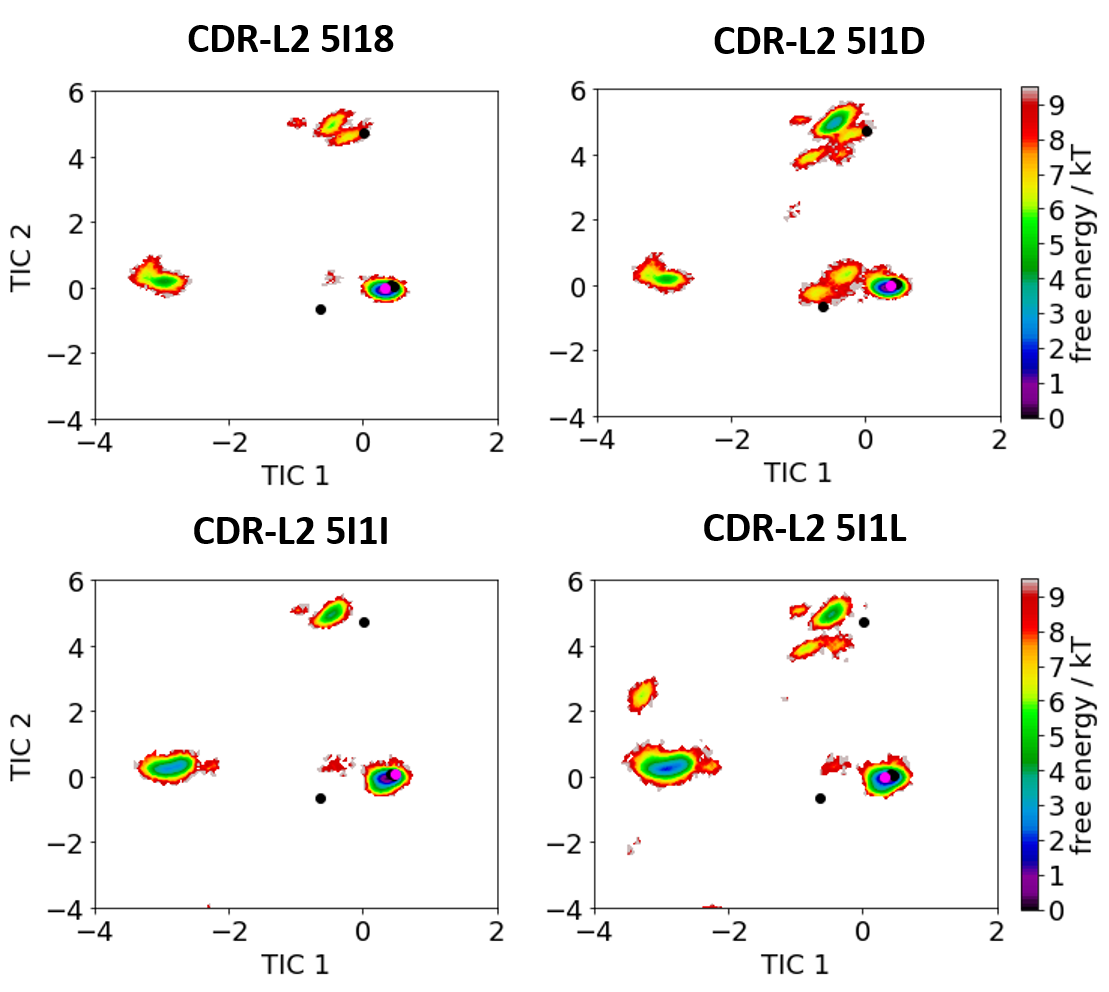
**

SI Figure S30: Comparison of the CDR-L1 loops consisting of the heavy chain germline L4-1. The free energy surface of the CDR-L2 loop paired with different light chain germlines in the same coordinate system is illustrated. The available canonical cluster structure representative for the CDR-L2 loop of length 8 is projected into the free energy surface and is depicted in black. In pink the respective crystal structures, which were used as starting structures are illustrated (PDB accession codes: 5I18, 5I1D, 5I1I and 5I1L).

**
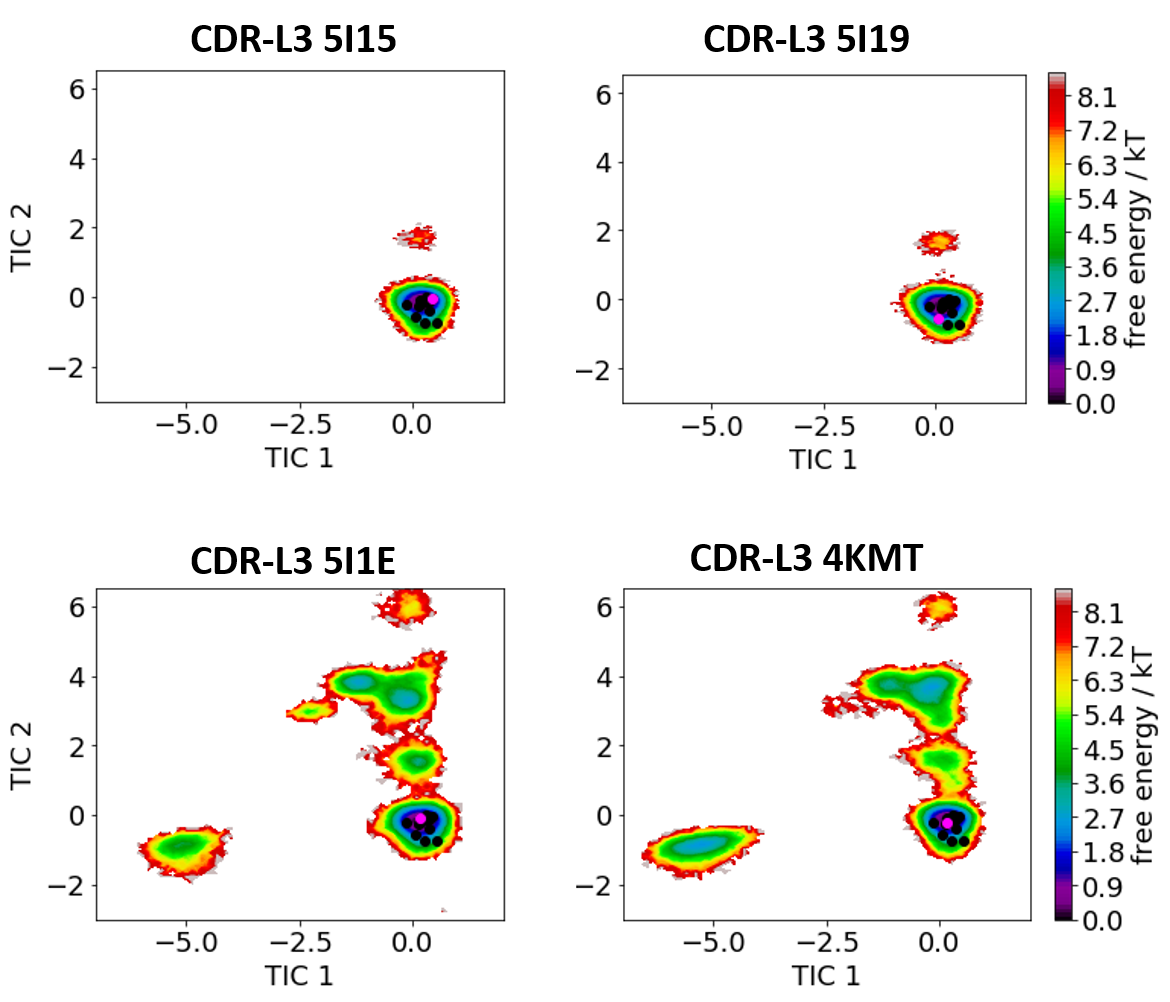
**

SI Figure S31: Comparison of the CDR-L3 loops consisting of the heavy chain germline L1-39. The free energy surface of the CDR-L3 loop paired with different light chain germlines in the same coordinate system is illustrated. The available 16 CDR-L3 loop conformations, resulting from different heavy and light chain pairings, are depicted in black. In pink the respective crystal structures, which were used as starting structures are illustrated (PDB accession codes: 5I15, 5I19, 5I1E and 4KMT).

**
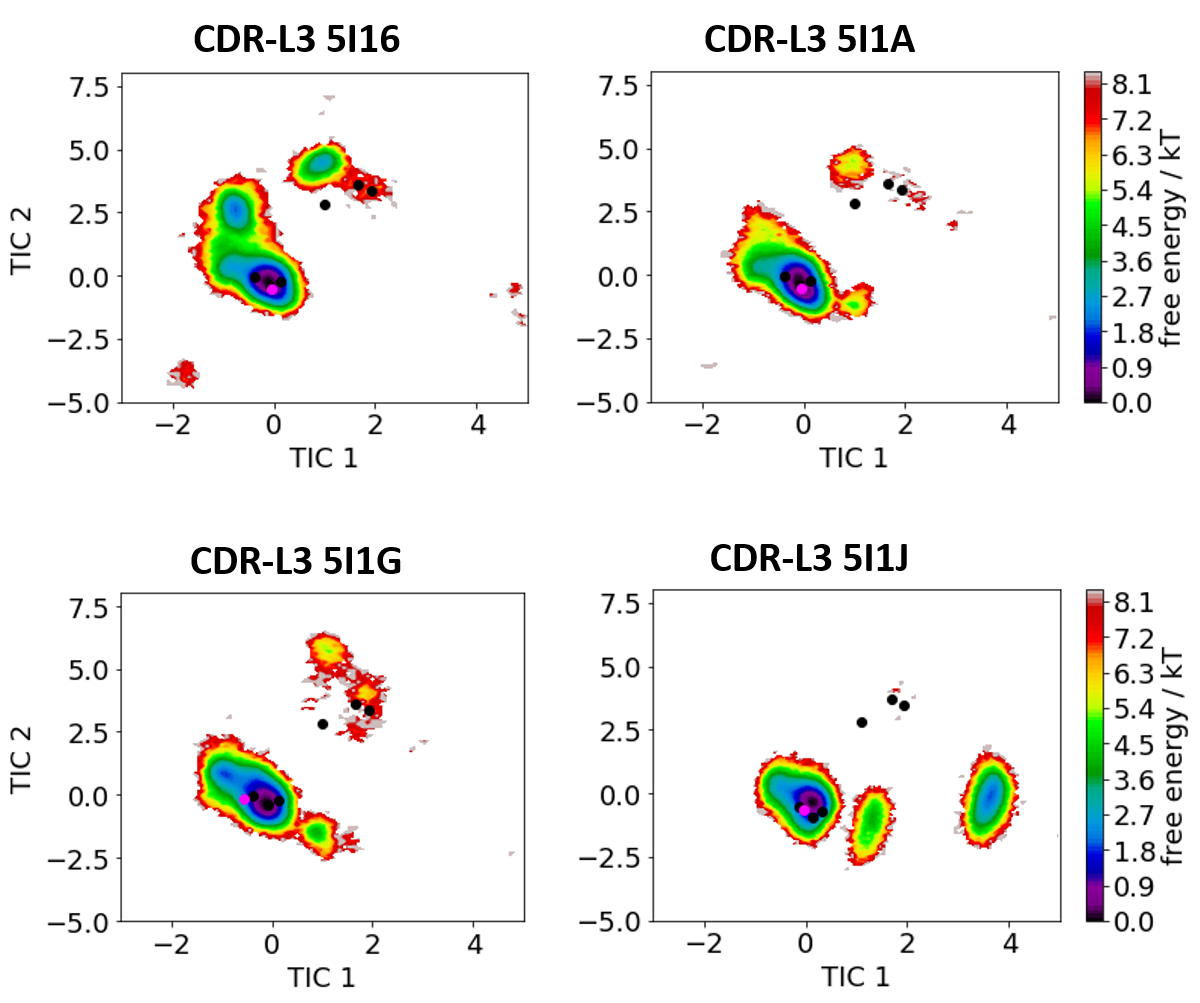
**

SI Figure S32: Comparison of the CDR-L3 loops consisting of the heavy chain germline L1-39. The free energy surface of the CDR-L3 loop paired with different light chain germlines in the same coordinate system is illustrated. The available canonical cluster structure representative for the CDR-L3 loop of length 9 is projected into the free energy surface and is depicted in black. In pink the respective crystal structures, which were used as starting structures are illustrated (PDB accession codes: 5I16, 5I1A, 5I1G and 5I1J).

**
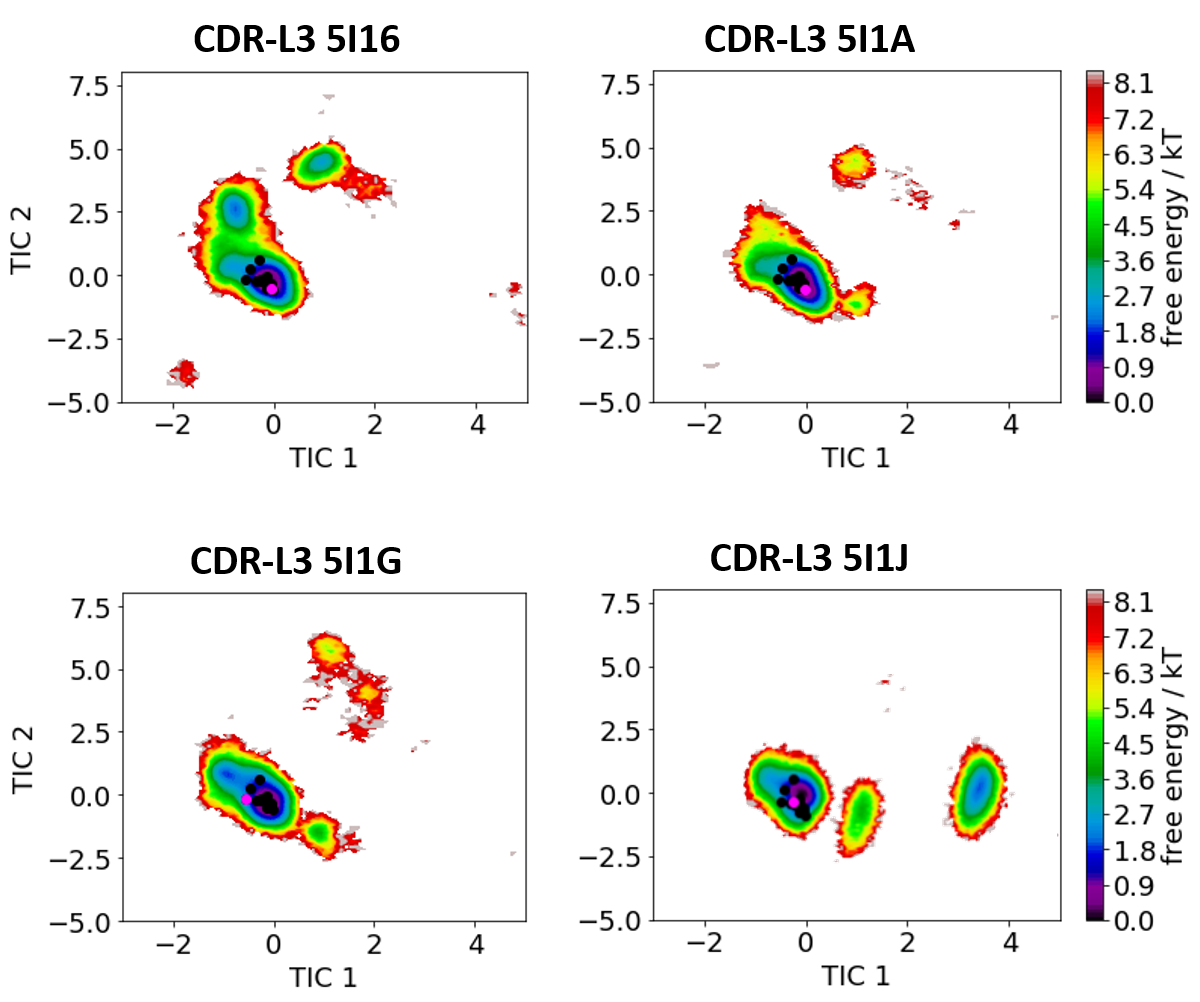
**

SI Figure S33: Comparison of the CDR-L3 loops consisting of the heavy chain germline L3-11. The free energy surface of the CDR-L3 loop paired with different light chain germlines in the same coordinate system is illustrated. The available 16 CDR-L3 loop conformations, resulting from different heavy and light chain pairings, are depicted in black. In pink the respective crystal structures, which were used as starting structures are illustrated (PDB accession codes: 5I16, 5I1A, 5I1G and 5I1J).

**
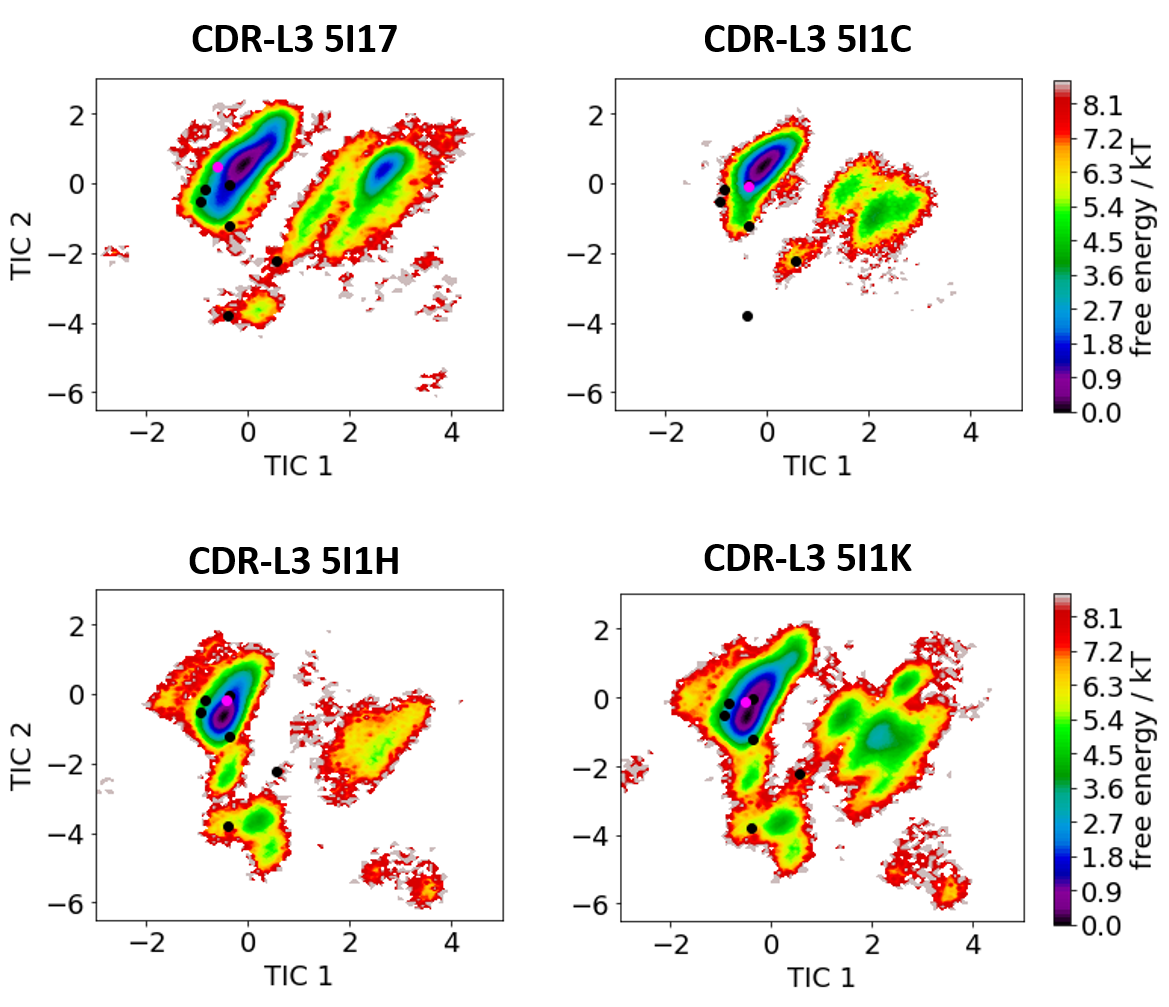
**

SI Figure S34: Comparison of the CDR-L3 loops consisting of the heavy chain germline L3-11. The free energy surface of the CDR-L3 loop paired with different light chain germlines in the same coordinate system is illustrated. The available canonical cluster structure representative for the CDR-L3 loop of length 9 is projected into the free energy surface and is depicted in black. In pink the respective crystal structures, which were used as starting structures are illustrated (PDB accession codes: 5I17, 5I1C, 5I1H and 5I1K).

**
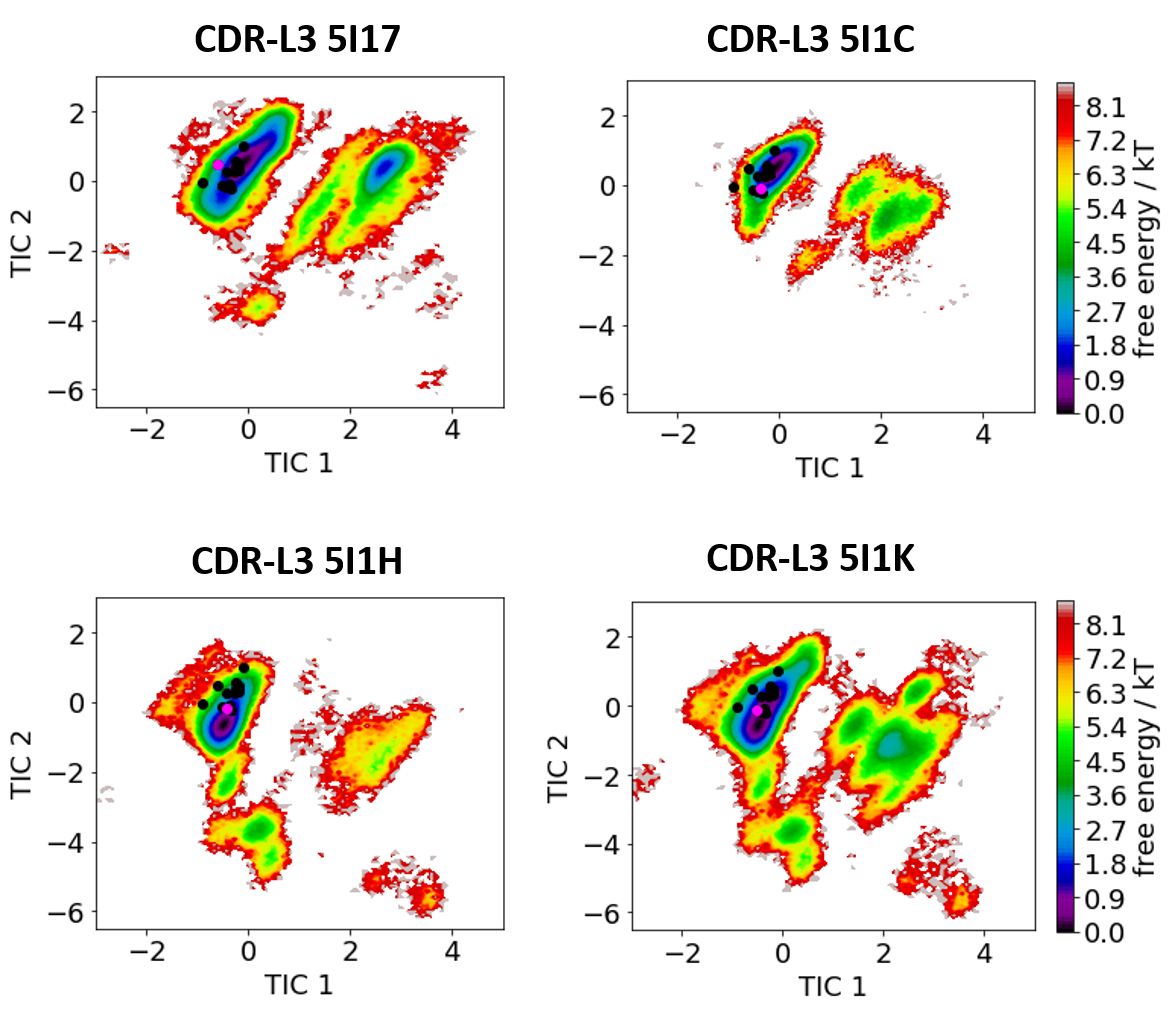
**

SI Figure S35: Comparison of the CDR-L3 loops consisting of the heavy chain germline L3-20. The free energy surface of the CDR-L3 loop paired with different light chain germlines in the same coordinate system is illustrated. The available 16 CDR-L3 loop conformations, resulting from different heavy and light chain pairings, are depicted in black. In pink the respective crystal structures, which were used as starting structures are illustrated (PDB accession codes: 5I17, 5I1C, 5I1H and 5I1K).


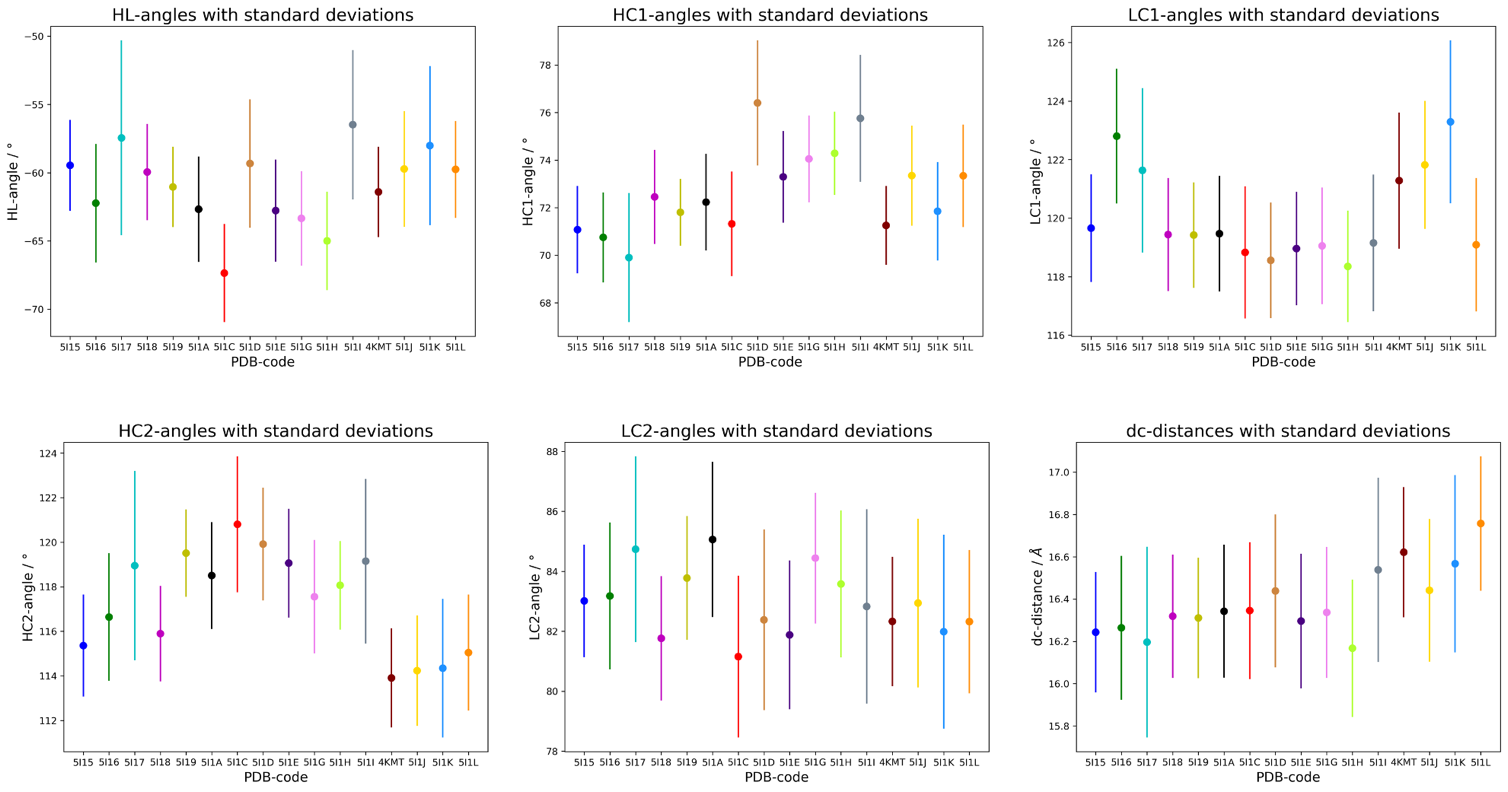


SI Figure S36: Overview of all six ABangle measures for all investigated 16 Fabs, which are used to characterize the relative interdomain orientation. The plots show the average and the standard deviation of each system and shows shifts and distinct favored interdomain orientations among all antibodies and all measures.


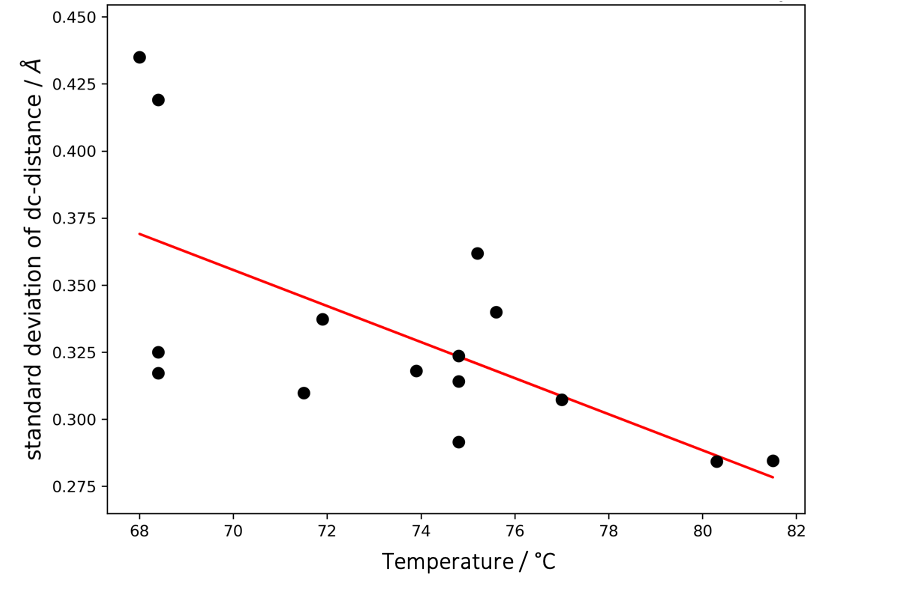


SI Figure S37: Correlation of the standard deviation of the distance vector (dc) with the melting temperature (R=0.64). We find that fluctuations in the distance between the two variable domains seem to influence thermal stability of Fabs.


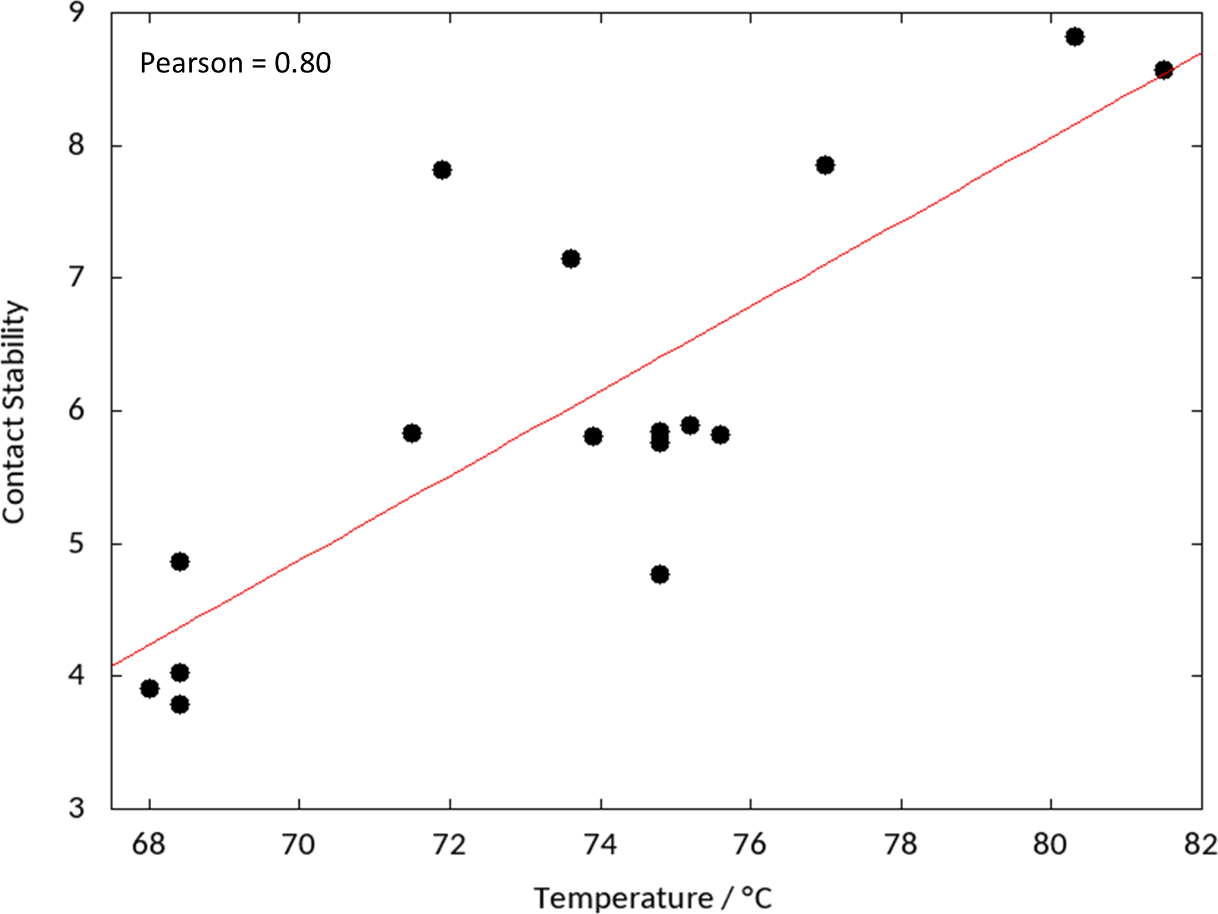


SI Figure S38: Correlation of the contacts stability (determined by the duration and number of contacts) with the melting temperature (R=0.8). We find that the higher the contact stability is, the lower is the experimentally determined melting temperature.


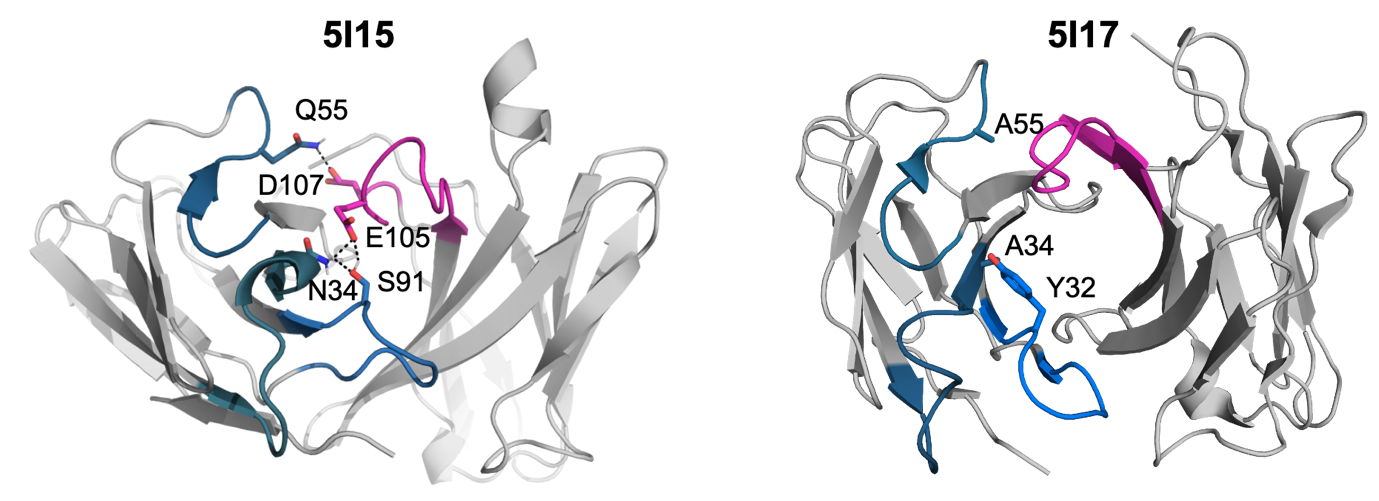


SI Figure S39: Structure representatives for the H1-69:L1-39 (5I15) and the H1-69:L3-20, highlighting the role of Q55, N34 and S91 as important interaction partners with the CDR-H3 loop in the H1-69:L1-39 Fab. The H1-69:L3-20 Fab has on both positions an alanine residue, which does not allow hydrogen bond interactions with the CDR-H3 loop. However, Y32 is still in close proximity to the Y103 located in the CDR-H3 loop and still allows to form hydrogen bond interactions.


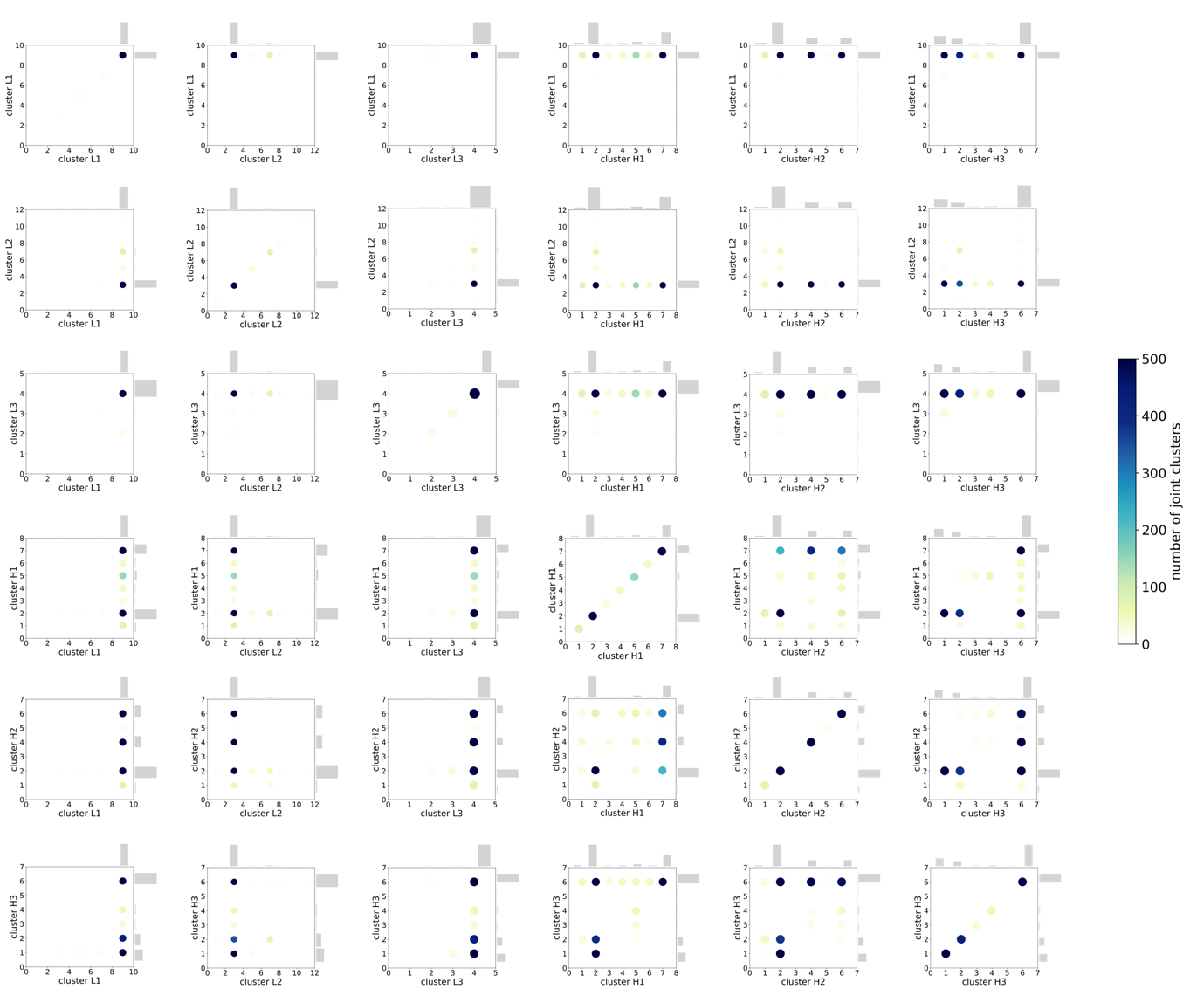


SI Figure S40: Visualization of simultaneous occurrence of CDR loop conformations for all CDR loops of the H1-69:L1-39 antibody. We observe strong correlations between the different CDR loop conformations. The individual CDR loops are clustered geometrically, and the resulting cluster populations are plotted against each other to visualize and describe the simultaneous occurrence of distinct loop conformations. Here, a matrix is illustrated including all individual CDR loops and displays which loop conformations occur at the same time and therefore might be correlated.

Table S2: Quantification of the flexibility for the individual CDR loops, presented in the free energy surfaces in Figure 1 and Figure 2, which is reflected in the number of clusters by using the same RMSD distance cut-off criterion of 1.2 A for all light chain CDR loops and 2.5 A for all heavy chain CDR loops.

| **CDR-L1 loop** | **Number of clusters** |
| --- | --- |
| H1-69:L1-39 | 12 |
| H3-23:L1-39 | 9 |
| H3-53:L1-39 | 52 |
| H5-51:L1-39 | 101 |
|  |  |
| **CDR-L2 loop** | **Number of clusters** |
| H1-69:L1-39 | 10 |
| H3-23:L1-39 | 87 |
| H3-53:L1-39 | 92 |
| H5-51:L1-39 | 24 |
|  |  |
| **CDR-L3 loop** | **Number of clusters** |
| H1-69:L1-39 | 4 |
| H3-23:L1-39 | 14 |
| H3-53:L1-39 | 27 |
| H5-51:L1-39 | 23 |
|  |  |
| **CDR-H1 loop** | **Number of clusters** |
| H1-69:L1-39 | 175 |
| H1-69:L3-11 | 136 |
| H1-69:L3-20 | 147 |
| H1-69:L4-1 | 128 |
|  |  |
| **CDR-H2 loop** | **Number of clusters** |
| H1-69:L1-39 | 16 |
| H1-69:L3-11 | 26 |
| H1-69:L3-20 | 37 |
| H1-69:L4-1 | 29 |
|  |  |
| **CDR-H3 loop** | **Number of clusters** |
| H1-69:L1-39 | 18 |
| H1-69:L3-11 | 18 |
| H1-69:L3-20 | 22 |
| H1-69:L4-1 | 19 |

Table S3: Quantitative overview of the conserved core interactions for all 16 antibody Fabs including the occurrence in %.

| core contacts | 5I19 | 5I15 | 5I16 | 5I17 | 5I18 | 5I1A | 5I1C | 5I1D | 5I1E | 5I1G | 5I1H | 5I1I | 5I1L | 5I1J | 5I1K | 4KMT |
| --- | --- | --- | --- | --- | --- | --- | --- | --- | --- | --- | --- | --- | --- | --- | --- | --- |
| L-Q38:H-Q39 | 98% | 97% | 89% | 95% | 94% | 89% | 79% | 92% | 94% | 93% | 86% | 94% | 87% | 91% | 88% | 92% |
| H-L45:L-Y97 | 82% | 75% | 81% | 85% | 77% | 80% | 76% | 73% | 80% | 79% | 81% | 77% | 74% | 79% | 86% | 75% |
| H-L45:L-F98 | 92% | 92% | 91% | 90% | 93% | 90% | 90% | 90% | 89% | 81% | 91% | 89% | 92% | 90% | 89% | 92% |
| L-P44:H-W103 | 94% | 94% | 94% | 90% | 92% | 92% | 96% | 86% | 94% | 92% | 93% | 79% | 73% | 84% | 85% | 94% |
| L-A43:H-Y91 | 83% | 80% | 71% | <70% | <70% | 73% | <70% | <70% | 77% | 73% | 75% | <70% | <70% | 77% | <70% | <70% |

Table S4: Occurrence in % of the interactions formed between the L1-39 germline specific residue L-N34 and the CDR-H3 loop.

| 5I15 interaction pairs | % | 5I19 interaction pairs | % | 5I1E interaction pairs | % | 4KMT interaction pairs | % |
| --- | --- | --- | --- | --- | --- | --- | --- |
| N34-E105 | 74 | N34-E105 | 85 | N34-E105 | 50 | N34-E105 | 73 |
| N34-G104 | 40 | N34-G104 | 44 | N34-G104 | 54 | N34-G104 | 58 |
| N34-Y103 | 31 | N34-Y103 | 46 | N34-Y103 | 51 | N34-Y103 | 42 |

Table S5: Occurrence in % of the interactions formed between residue L-Q/E55 and the CDR-H3 loop.

| 5I15 | % | 5I19 | % | 5I1E | % | 4KMT | % | 5I18 | % | 5I1L | % | 5I1I | % | 5I1D | % |
| --- | --- | --- | --- | --- | --- | --- | --- | --- | --- | --- | --- | --- | --- | --- | --- |
| Q55-D107 | 42 | Q55-D107 | 53 | Q55-E105 | 18 | Q55-D107 | 29 | E55-D107 | 4 | E55-D107 | 1 | E55-L106 | 14 | E55-D107 | 2 |
| Q55-G316 | 15 | Q55-D100 | 4 | Q55-D107 | 15 |  |  |  |  |  |  |  |  |  |  |

Table S6: Quantitative overview of the interdomain interactions of the H1-69:L1-39 (5I15) Fab.

| Heavy chain | Light chain | Occurence / % |
| --- | --- | --- |
| LEU106 | TYR36 | 97.51 |
| PHE172 | SER176 | 97.39 |
| GLN39 | GLN38 | 96.69 |
| TRP109 | PRO44 | 96.50 |
| TRP47 | LEU96 | 92.61 |
| LEU45 | PHE98 | 92.49 |
| PHE128 | GLN124 | 91.95 |
| TRP47 | PRO95 | 91.09 |
| TYR103 | SER91 | 88.68 |
| PHE172 | THR164 | 84.86 |
| PRO173 | VAL163 | 82.84 |
| PRO173 | SER162 | 81.32 |
| TYR95 | ALA43 | 80.12 |
| GLY110 | ALA43 | 76.69 |
| PHE172 | LEU175 | 76.61 |
| ALA143 | PHE118 | 76.30 |
| PHE172 | SER174 | 76.26 |
| PHE172 | SER162 | 75.99 |
| LEU45 | TYR87 | 75.68 |
| GLU105 | ASN34 | 73.97 |
| PRO129 | SER121 | 72.84 |
| LEU147 | SER131 | 69.22 |
| LEU130 | PHE118 | 68.75 |
| ALA131 | PRO119 | 67.08 |
| TYR95 | GLN38 | 67.04 |
| GLY104 | SER91 | 64.75 |
| ALA131 | PHE118 | 63.97 |
| LEU130 | VAL133 | 63.04 |
| LYS220 | ASP122 | 62.68 |
| ALA143 | PHE116 | 58.99 |
| GLN39 | TYR87 | 58.87 |
| PHE172 | LEU135 | 56.46 |
| TRP109 | TYR36 | 55.91 |
| ASP107 | LEU46 | 54.86 |
| GLU105 | LEU46 | 54.67 |
| LEU144 | PHE118 | 53.81 |
| VAL175 | GLN160 | 50.54 |
| TRP47 | THR94 | 50.47 |
| PHE128 | SER121 | 49.77 |
| GLU105 | TYR49 | 48.91 |
| LYS215 | GLU123 | 48.33 |
| TYR103 | TYR32 | 47.47 |
| TYR103 | ALA50 | 46.38 |
| SER185 | SER176 | 46.30 |
| VAL187 | LEU135 | 45.06 |
| TYR95 | PRO44 | 44.71 |
| GLY104 | GLN89 | 43.23 |
| ASP107 | GLN55 | 42.57 |
| LEU130 | PRO119 | 41.05 |
| GLY104 | ASN34 | 40.43 |
| ASN59 | THR94 | 40.39 |
| LEU106 | LEU46 | 40.19 |

Table S7: Quantitative overview of the interdomain interactions of the H1-69:L4-1 (5I18) Fab.

| Heavy | Light | Percent |
| --- | --- | --- |
| LEU106 | TYR43 | 97.88 |
| TRP109 | PRO51 | 96.31 |
| PHE172 | SER183 | 95.62 |
| GLN39 | GLN45 | 94.36 |
| LEU45 | PHE105 | 93.79 |
| PRO173 | SER169 | 89.78 |
| TRP47 | PRO102 | 89.69 |
| TRP47 | LEU103 | 88.92 |
| GLY110 | PRO50 | 88.52 |
| PHE128 | GLN131 | 86.40 |
| PHE172 | LEU182 | 82.47 |
| GLY104 | TYR98 | 80.27 |
| PHE172 | SER181 | 78.24 |
| LEU45 | TYR94 | 77.35 |
| PHE172 | THR171 | 73.25 |
| ALA131 | PRO126 | 71.22 |
| PRO129 | SER128 | 70.73 |
| TYR95 | PRO50 | 70.42 |
| LEU130 | PHE125 | 70.30 |
| LEU147 | SER138 | 70.22 |
| PRO173 | VAL170 | 68.59 |
| TYR95 | GLN45 | 68.18 |
| PHE172 | LEU142 | 66.87 |
| ALA143 | PHE125 | 66.35 |
| SER185 | SER183 | 65.89 |
| GLN39 | TYR94 | 64.83 |
| LEU130 | VAL140 | 64.49 |
| TYR103 | TYR98 | 60.11 |
| TRP47 | THR101 | 57.96 |
| HIE170 | THR171 | 57.79 |
| ALA131 | PHE125 | 56.79 |
| PHE172 | SER169 | 53.89 |
| LEU144 | PHE125 | 52.61 |
| VAL175 | GLN167 | 50.83 |
| GLU105 | LEU53 | 47.05 |
| VAL175 | GLU168 | 46.25 |
| LYS215 | GLU130 | 45.50 |
| LEU130 | PRO126 | 44.99 |
| TRP109 | TYR43 | 43.64 |
| GLU105 | TYR56 | 42.96 |
| GLU105 | TYR98 | 41.24 |

Table S8: Quantitative overview of the interdomain interactions of the H1-69:L3-20 (5I17) Fab.

| Heavy | Light | Percent |
| --- | --- | --- |
| TRP47 | LEU97 | 96.21 |
| GLN39 | GLN39 | 95.29 |
| PHE172 | SER177 | 93.13 |
| PHE128 | GLN125 | 91.66 |
| LEU45 | PHE99 | 90.76 |
| TRP109 | PRO45 | 90.52 |
| TRP47 | PRO96 | 85.61 |
| LEU45 | TYR88 | 85.40 |
| ALA131 | PHE119 | 77.87 |
| PHE172 | THR165 | 75.42 |
| PHE172 | SER175 | 74.36 |
| PHE172 | LEU176 | 73.65 |
| PRO173 | SER163 | 72.78 |
| ASP107 | LEU47 | 71.74 |
| ALA143 | PHE119 | 71.01 |
| TRP109 | TYR37 | 70.54 |
| LEU130 | PHE119 | 69.32 |
| PRO173 | VAL164 | 68.12 |
| PHE172 | SER163 | 67.14 |
| TYR95 | ALA44 | 63.16 |
| PRO129 | SER122 | 62.62 |
| ALA143 | PHE117 | 58.99 |
| TYR99 | TYR37 | 57.71 |
| ALA131 | PRO120 | 57.33 |
| GLY44 | TYR88 | 56.24 |
| LEU147 | SER132 | 55.86 |
| LEU130 | VAL134 | 53.92 |
| PHE172 | LEU136 | 53.73 |
| ASN59 | SER95 | 53.32 |
| GLY104 | TYR50 | 52.70 |
| GLU105 | TYR50 | 51.42 |
| HIE170 | THR165 | 51.01 |
| LYS215 | GLU124 | 50.79 |
| VAL187 | LEU136 | 47.14 |
| LEU144 | PHE119 | 45.64 |
| GLU105 | THR57 | 45.61 |
| PHE128 | SER122 | 45.18 |
| VAL37 | PHE99 | 44.99 |
| VAL175 | GLN161 | 44.47 |
| LEU45 | PRO45 | 43.73 |
| HIE170 | SER175 | 42.92 |
| GLY110 | ALA44 | 42.92 |
| PHE128 | GLU124 | 42.86 |
| SER185 | SER177 | 42.43 |
| TRP47 | SER95 | 41.91 |
| TYR103 | TYR92 | 40.27 |

Table S9: Quantitative overview of the interdomain interactions of the H1-69:L3-11 (5I16) Fab.

| Heavy | Light | Percent |
| --- | --- | --- |
| LEU106 | TYR36 | 95.51 |
| TRP109 | PRO44 | 95.34 |
| PHE172 | SER176 | 92.95 |
| LEU45 | PHE98 | 91.68 |
| PHE128 | GLN124 | 91.44 |
| GLN39 | GLN38 | 89.73 |
| TRP47 | LEU96 | 89.01 |
| LEU45 | TYR87 | 81.44 |
| TRP47 | PRO95 | 80.79 |
| TRP47 | TRP94 | 78.49 |
| GLY110 | ALA43 | 77.50 |
| ALA143 | PHE118 | 76.54 |
| PHE172 | SER174 | 76.16 |
| PHE172 | THR164 | 75.38 |
| PRO173 | VAL163 | 72.77 |
| LEU130 | PHE118 | 72.53 |
| ALA143 | PHE116 | 72.29 |
| TYR95 | ALA43 | 71.92 |
| GLY104 | ARG91 | 71.92 |
| PHE172 | LEU175 | 69.14 |
| ALA131 | PHE118 | 68.84 |
| PHE172 | SER162 | 68.80 |
| GLU105 | LEU46 | 66.75 |
| TYR95 | GLN38 | 66.61 |
| TRP109 | TYR36 | 64.04 |
| PRO129 | SER121 | 61.13 |
| PRO173 | SER162 | 60.96 |
| GLU105 | TYR49 | 60.34 |
| TYR103 | ARG91 | 59.35 |
| ALA131 | PRO119 | 58.36 |
| ASN59 | TRP94 | 54.49 |
| GLN39 | TYR87 | 54.42 |
| LEU130 | VAL133 | 51.78 |
| PHE128 | GLU123 | 50.89 |
| TYR103 | TYR49 | 50.55 |
| PHE172 | LEU135 | 50.17 |
| VAL187 | LEU135 | 47.40 |
| TYR95 | PRO44 | 46.75 |
| GLU105 | TYR36 | 46.51 |
| LEU144 | PHE118 | 46.20 |
| LYS215 | GLU123 | 45.96 |
| ALA142 | PHE116 | 45.82 |
| PHE128 | SER121 | 45.10 |
| THR189 | ASN137 | 43.46 |
| TYR99 | ARG91 | 41.92 |
| HIE170 | SER174 | 41.92 |
| LEU147 | SER131 | 40.99 |
| HIE170 | THR164 | 40.89 |

Table S10: Quantitative overview of the interdomain interactions of the H3-23:L1-39 (5I19) Fab.

| Heavy | Light | Percent |
| --- | --- | --- |
| LEU106 | TYR36 | 99.48 |
| GLN39 | GLN38 | 97.38 |
| PHE172 | SER176 | 96.85 |
| PRO173 | SER162 | 96.06 |
| PHE128 | GLN124 | 95.80 |
| TRP109 | PRO44 | 94.49 |
| LEU45 | PHE98 | 91.86 |
| TRP47 | PRO95 | 91.86 |
| TYR95 | ALA43 | 87.66 |
| TYR103 | SER91 | 87.40 |
| TRP47 | LEU96 | 86.88 |
| GLU105 | ASN34 | 85.30 |
| GLU105 | TYR49 | 84.51 |
| PHE172 | LEU175 | 83.73 |
| LEU45 | TYR87 | 81.89 |
| PHE172 | THR164 | 80.84 |
| GLY110 | ALA43 | 80.31 |
| PHE172 | SER174 | 77.17 |
| LEU147 | SER131 | 76.38 |
| ALA143 | PHE118 | 76.12 |
| PHE172 | SER162 | 74.54 |
| PRO173 | VAL163 | 72.70 |
| TYR95 | GLN38 | 71.65 |
| PRO129 | SER121 | 70.87 |
| HIE170 | THR164 | 70.34 |
| GLU105 | LEU46 | 68.77 |
| TYR103 | ALA50 | 67.45 |
| TYR99 | LEU96 | 67.45 |
| LEU130 | PHE118 | 65.88 |
| LYS220 | ASP122 | 65.35 |
| LEU130 | VAL133 | 64.30 |
| SER185 | SER176 | 64.30 |
| TRP109 | TYR36 | 63.52 |
| TYR103 | TYR32 | 63.52 |
| TRP47 | THR94 | 61.42 |
| LYS135 | ILE117 | 60.89 |
| PHE172 | LEU135 | 60.37 |
| SER136 | PHE116 | 59.06 |
| LEU45 | PRO44 | 58.53 |
| VAL175 | GLN160 | 58.27 |
| ALA131 | PHE118 | 57.48 |
| LYS135 | SER208 | 57.22 |
| LYS215 | GLU123 | 55.91 |
| GLY104 | SER91 | 55.38 |
| GLN39 | TYR87 | 54.07 |
| SER136 | PHE118 | 54.07 |
| ASP107 | GLN55 | 53.28 |
| LEU144 | PHE118 | 52.23 |
| VAL175 | GLU161 | 50.66 |
| ALA143 | PHE116 | 48.03 |
| TYR103 | ASN34 | 46.98 |
| LEU106 | LEU46 | 44.88 |
| LYS149 | THR180 | 44.62 |
| LYS149 | SER131 | 44.36 |
| GLY104 | ASN34 | 43.83 |
| LYS135 | PHE116 | 43.04 |
| PHE128 | SER121 | 42.78 |
| ASP107 | LEU46 | 42.26 |
| ALA131 | PRO119 | 41.73 |
| TYR95 | PRO44 | 41.73 |

Table S11: Quantitative overview of the interdomain interactions of the H3-23:L3-11 (5I1A) Fab.

| Heavy | Light | Percent |
| --- | --- | --- |
| PHE172 | SER176 | 95.37 |
| TRP109 | PRO44 | 92.28 |
| PHE128 | GLN124 | 91.95 |
| LEU45 | PHE98 | 91.87 |
| LEU106 | TYR36 | 91.14 |
| GLN39 | GLN38 | 89.43 |
| GLY110 | ALA43 | 84.31 |
| PHE172 | THR164 | 82.40 |
| LEU45 | TYR87 | 81.91 |
| TRP47 | TRP94 | 78.17 |
| GLY104 | ARG91 | 75.49 |
| TYR95 | GLN38 | 75.12 |
| TYR95 | ALA43 | 74.96 |
| PRO173 | VAL163 | 74.47 |
| TRP47 | LEU96 | 74.47 |
| ALA143 | PHE118 | 74.31 |
| PHE172 | LEU175 | 73.70 |
| PHE172 | SER174 | 73.13 |
| TYR59 | TRP94 | 72.97 |
| PRO129 | SER121 | 72.89 |
| PHE172 | SER162 | 72.72 |
| LEU130 | PHE118 | 69.07 |
| PRO173 | SER162 | 68.98 |
| TRP109 | TYR36 | 68.78 |
| TRP47 | PRO95 | 68.33 |
| LYS220 | ASP122 | 66.22 |
| ALA131 | PHE118 | 61.54 |
| TYR99 | TRP94 | 58.82 |
| ALA143 | PHE116 | 55.16 |
| LEU130 | VAL133 | 54.51 |
| HIE170 | THR164 | 53.01 |
| LYS215 | GLU123 | 52.80 |
| TYR99 | ARG91 | 52.44 |
| GLN39 | TYR87 | 52.40 |
| LEU147 | SER131 | 52.36 |
| GLU105 | TYR49 | 51.75 |
| PHE172 | LEU135 | 51.54 |
| TYR103 | TYR49 | 51.34 |
| ALA131 | PRO119 | 50.93 |
| GLY104 | GLN89 | 50.33 |
| LEU45 | PRO44 | 48.86 |
| PHE128 | GLU123 | 47.11 |
| TYR103 | ARG91 | 46.87 |
| PHE128 | SER121 | 46.75 |
| GLU105 | LEU46 | 45.04 |
| LEU144 | PHE118 | 44.15 |
| VAL187 | LEU135 | 42.93 |
| GLU105 | TYR36 | 41.99 |
| SER185 | SER176 | 41.26 |
| GLY44 | TYR87 | 40.53 |

Table S12: Quantitative overview of the interdomain interactions of the H3-23:L3-20 (5I1C) Fab.

| Heavy | Light | Percent |
| --- | --- | --- |
| TRP109 | PRO45 | 96.86 |
| PHE128 | GLN125 | 94.73 |
| LEU45 | PHE99 | 92.81 |
| LEU106 | TYR37 | 92.33 |
| PHE172 | SER177 | 88.34 |
| LEU45 | TYR88 | 86.32 |
| TRP47 | LEU97 | 85.20 |
| PRO173 | SER163 | 84.77 |
| GLY110 | ALA44 | 83.71 |
| GLY104 | TYR92 | 82.85 |
| PHE172 | SER175 | 79.98 |
| GLN39 | GLN39 | 79.34 |
| PHE172 | LEU176 | 78.59 |
| TRP47 | PRO96 | 76.14 |
| GLU105 | TYR92 | 74.49 |
| TYR103 | TYR92 | 74.28 |
| LEU130 | PHE119 | 72.42 |
| PHE172 | THR165 | 69.86 |
| PRO173 | VAL164 | 69.86 |
| ALA143 | PHE119 | 69.28 |
| PRO129 | SER122 | 67.94 |
| TYR95 | GLN39 | 66.61 |
| LEU106 | LEU47 | 65.87 |
| PHE172 | LEU136 | 62.19 |
| LYS220 | ASP123 | 61.29 |
| LEU147 | SER132 | 61.02 |
| ALA131 | PHE119 | 60.65 |
| TYR103 | TYR33 | 60.44 |
| GLU105 | TYR50 | 59.64 |
| ALA131 | PRO120 | 59.11 |
| TRP109 | TYR37 | 58.95 |
| TYR95 | ALA44 | 56.39 |
| SER185 | SER177 | 55.86 |
| LEU130 | VAL134 | 54.42 |
| PHE172 | SER163 | 53.67 |
| VAL187 | LEU136 | 53.09 |
| VAL175 | GLN161 | 53.09 |
| GLY44 | TYR88 | 52.13 |
| LYS215 | GLU124 | 51.38 |
| GLN39 | TYR88 | 51.06 |
| ALA143 | PHE117 | 50.69 |
| LEU144 | PHE119 | 49.36 |
| PHE128 | SER122 | 48.62 |
| LYS149 | SER132 | 47.39 |
| HIE170 | SER175 | 46.33 |
| HIE170 | THR165 | 45.85 |
| GLY145 | PHE119 | 42.60 |
| GLY104 | GLN90 | 41.91 |
| VAL175 | GLU162 | 41.27 |
| PHE128 | GLU124 | 40.52 |
| LEU45 | PRO45 | 40.15 |

Table S13: Quantitative overview of the interdomain interactions of the H3-23:L4-1 (5I1D) Fab.

| Heavy | Light | Percent |
| --- | --- | --- |
| CYX222 | CYX220 | 100.00 |
| GLN39 | GLN44 | 95.51 |
| PHE172 | SER182 | 94.91 |
| PHE128 | GLN130 | 93.48 |
| LEU45 | PHE104 | 92.06 |
| TRP47 | PRO101 | 91.12 |
| TRP109 | PRO50 | 87.00 |
| PRO173 | SER168 | 82.17 |
| TRP47 | LEU102 | 81.57 |
| PHE172 | LEU181 | 80.86 |
| LYS220 | ASP128 | 78.46 |
| PHE172 | SER180 | 77.19 |
| PRO129 | SER127 | 75.47 |
| ALA143 | PHE124 | 74.38 |
| LEU45 | TYR93 | 73.22 |
| ALA131 | PRO125 | 73.07 |
| PHE172 | THR170 | 70.94 |
| LEU130 | PHE124 | 70.45 |
| PRO173 | VAL169 | 66.44 |
| GLY110 | PRO49 | 64.04 |
| LEU147 | SER137 | 63.67 |
| LEU130 | VAL139 | 62.66 |
| TRP47 | THR100 | 62.55 |
| TYR95 | PRO49 | 62.10 |
| PHE172 | LEU141 | 61.05 |
| PHE172 | SER168 | 60.15 |
| GLN39 | TYR93 | 59.10 |
| ALA131 | PHE124 | 57.94 |
| HIE170 | THR170 | 57.04 |
| SER185 | SER182 | 55.39 |
| LEU144 | PHE124 | 55.39 |
| TYR95 | GLN44 | 54.42 |
| LYS215 | GLU129 | 52.02 |
| TYR103 | TYR97 | 51.16 |
| LEU106 | TYR42 | 50.97 |
| ALA143 | PHE122 | 50.97 |
| TRP109 | TYR42 | 50.15 |
| GLN111 | PRO49 | 44.94 |
| TYR99 | LEU102 | 44.91 |
| VAL175 | GLN166 | 44.42 |
| PHE128 | SER127 | 43.63 |
| LEU130 | PRO125 | 43.30 |
| LEU45 | PRO50 | 43.22 |
| VAL187 | LEU141 | 42.81 |

Table S14: Quantitative overview of the interdomain interactions of the H3-53:L1-39 (5I1E) Fab.

| Heavy | Light | Percent |
| --- | --- | --- |
| LEU105 | TYR36 | 95.94 |
| PHE171 | SER176 | 95.82 |
| GLN39 | GLN38 | 94.88 |
| PHE127 | GLN124 | 94.65 |
| TRP108 | PRO44 | 94.24 |
| LEU45 | PHE98 | 89.47 |
| TRP47 | PRO95 | 87.47 |
| TYR102 | SER91 | 87.29 |
| PRO172 | SER162 | 83.88 |
| TRP47 | LEU96 | 83.71 |
| LEU45 | TYR87 | 80.88 |
| PHE171 | LEU175 | 80.41 |
| GLY109 | ALA43 | 79.94 |
| TYR94 | ALA43 | 77.88 |
| PRO128 | SER121 | 77.12 |
| PHE171 | SER174 | 75.71 |
| PHE171 | THR164 | 75.18 |
| PRO172 | VAL163 | 74.12 |
| TYR94 | GLN38 | 70.18 |
| LEU129 | PHE118 | 67.65 |
| ALA142 | PHE118 | 66.41 |
| LEU146 | SER131 | 66.12 |
| TYR102 | TYR32 | 63.71 |
| GLY103 | SER91 | 61.82 |
| PHE171 | LEU135 | 60.76 |
| SER184 | SER176 | 58.94 |
| PHE171 | SER162 | 57.35 |
| LYS214 | GLU123 | 56.65 |
| TYR102 | TYR49 | 56.18 |
| LEU129 | VAL133 | 56.18 |
| ALA130 | PHE118 | 56.12 |
| GLN39 | TYR87 | 54.24 |
| GLY103 | ASN34 | 54.06 |
| HIE169 | THR164 | 52.82 |
| GLU104 | LEU46 | 52.41 |
| TYR102 | ASN34 | 51.76 |
| GLU104 | TYR49 | 51.71 |
| LYS219 | ASP122 | 51.29 |
| TRP108 | TYR36 | 50.88 |
| GLU104 | ASN34 | 50.53 |
| PHE127 | SER121 | 50.35 |
| LEU45 | PRO44 | 48.41 |
| GLY103 | GLN89 | 46.94 |
| VAL186 | LEU135 | 46.59 |
| VAL174 | GLN160 | 45.71 |
| ALA130 | PRO119 | 45.29 |
| GLY44 | TYR87 | 45.18 |
| LYS148 | SER131 | 44.47 |
| SER135 | PHE116 | 42.59 |
| TYR102 | ALA50 | 42.35 |
| VAL174 | GLU161 | 41.00 |
| GLN176 | GLN160 | 40.65 |

Table S15: Quantitative overview of the interdomain interactions of the H3-53:L3-11 (5I1G) Fab.

| Heavy | Light | Percent |
| --- | --- | --- |
| LEU105 | TYR36 | 96.68 |
| PHE171 | SER176 | 94.99 |
| GLN39 | GLN38 | 93.60 |
| TRP108 | PRO44 | 92.96 |
| LEU45 | PHE98 | 91.54 |
| PHE127 | GLN124 | 88.95 |
| TYR94 | ALA43 | 83.38 |
| GLY103 | ARG91 | 82.96 |
| GLY109 | ALA43 | 80.52 |
| LEU45 | TYR87 | 79.59 |
| TYR102 | ARG91 | 78.93 |
| PRO128 | SER121 | 78.12 |
| PHE171 | THR164 | 77.78 |
| PHE171 | SER174 | 75.16 |
| ALA130 | PRO119 | 74.87 |
| PHE171 | LEU175 | 74.41 |
| TYR94 | GLN38 | 73.06 |
| ALA142 | PHE118 | 72.97 |
| PRO172 | VAL163 | 72.87 |
| TRP47 | LEU96 | 72.16 |
| TRP47 | PRO95 | 71.30 |
| PRO172 | SER162 | 70.77 |
| ALA130 | PHE118 | 67.22 |
| PHE171 | SER162 | 65.73 |
| LEU129 | PHE118 | 65.41 |
| TYR58 | TRP94 | 64.39 |
| TRP108 | TYR36 | 61.43 |
| LEU129 | VAL133 | 60.74 |
| GLN39 | TYR87 | 59.59 |
| PHE171 | LEU135 | 58.76 |
| TRP47 | TRP94 | 58.67 |
| GLU104 | TYR49 | 57.88 |
| LEU146 | SER131 | 54.78 |
| GLU104 | ARG91 | 54.36 |
| LYS214 | GLU123 | 52.09 |
| LYS219 | ASP122 | 50.40 |
| ALA142 | PHE116 | 50.13 |
| LEU143 | PHE118 | 50.06 |
| HIE169 | THR164 | 49.03 |
| LEU129 | PRO119 | 48.06 |
| TYR94 | PRO44 | 47.47 |
| SER184 | SER176 | 47.30 |
| GLU104 | LEU46 | 46.52 |
| TYR98 | ARG91 | 44.37 |
| LEU45 | PRO44 | 44.29 |
| VAL186 | LEU135 | 43.19 |
| TYR98 | LEU96 | 42.26 |
| TRP108 | ARG45 | 41.46 |
| GLY109 | ARG45 | 41.07 |
| PHE127 | SER121 | 41.02 |
| GLN110 | ALA43 | 40.31 |

Table S16: Quantitative overview of the interdomain interactions of the H3-53:L3-20 (5I1H) Fab.

| Heavy | Light | Percent |
| --- | --- | --- |
| LEU105 | TYR37 | 96.40 |
| PHE171 | SER177 | 94.67 |
| TRP108 | PRO45 | 93.72 |
| LEU45 | PHE99 | 91.07 |
| PHE127 | GLN125 | 87.95 |
| GLN39 | GLN39 | 86.65 |
| PHE171 | THR165 | 85.89 |
| GLY109 | ALA44 | 82.38 |
| LEU45 | TYR88 | 81.59 |
| PRO172 | VAL164 | 79.49 |
| ALA142 | PHE119 | 78.82 |
| PHE171 | SER163 | 75.90 |
| TYR94 | GLN39 | 75.74 |
| TYR94 | ALA44 | 75.31 |
| LEU129 | PHE119 | 72.62 |
| PHE171 | SER175 | 71.75 |
| GLN39 | TYR88 | 68.43 |
| TRP108 | TYR37 | 67.84 |
| PHE171 | LEU176 | 67.48 |
| ALA130 | PRO120 | 67.33 |
| TRP47 | PRO96 | 67.33 |
| ALA130 | PHE119 | 67.29 |
| ALA142 | PHE117 | 66.61 |
| TYR102 | TYR92 | 62.70 |
| LEU129 | VAL134 | 56.93 |
| PRO128 | SER122 | 56.62 |
| TRP47 | LEU97 | 55.35 |
| GLY103 | TYR92 | 52.86 |
| GLU104 | TYR50 | 52.51 |
| PRO172 | SER163 | 52.43 |
| LEU143 | PHE119 | 52.03 |
| GLU104 | TYR37 | 50.53 |
| TYR94 | PRO45 | 50.14 |
| GLU104 | LEU47 | 49.43 |
| HIE169 | THR165 | 48.48 |
| THR188 | ASN138 | 47.14 |
| ALA141 | PHE117 | 45.59 |
| LYS214 | GLU124 | 45.28 |
| VAL186 | LEU136 | 45.04 |
| PHE127 | GLU124 | 44.37 |
| LEU105 | LEU47 | 44.37 |
| PHE171 | LEU136 | 43.97 |
| LEU45 | PRO45 | 41.76 |
| TYR102 | TYR33 | 41.64 |
| HIE169 | ASP168 | 40.14 |

Table S17: Quantitative overview of the interdomain interactions of the H3-53:L4-1 (5I1I) Fab.

| Heavy | Light | Percent |
| --- | --- | --- |
| GLN39 | GLN44 | 96.22 |
| PHE171 | SER182 | 95.56 |
| PHE127 | GLN130 | 93.33 |
| LEU45 | PHE104 | 89.78 |
| TRP47 | PRO101 | 88.00 |
| PRO172 | SER168 | 86.89 |
| PHE171 | LEU181 | 83.56 |
| TRP47 | LEU102 | 83.33 |
| TRP108 | PRO50 | 79.33 |
| PHE171 | SER180 | 79.11 |
| LEU129 | PHE124 | 77.78 |
| LEU45 | TYR93 | 77.56 |
| PHE171 | THR170 | 73.11 |
| PRO128 | SER127 | 72.67 |
| PRO172 | VAL169 | 69.11 |
| LEU146 | SER137 | 68.89 |
| ALA142 | PHE124 | 65.33 |
| ALA130 | PRO125 | 65.11 |
| ALA130 | PHE124 | 63.78 |
| LYS219 | ASP128 | 62.44 |
| ASP106 | TYR42 | 62.00 |
| LEU129 | VAL139 | 61.78 |
| TYR94 | PRO49 | 61.11 |
| PHE171 | SER168 | 60.67 |
| PHE171 | LEU141 | 57.56 |
| GLY109 | PRO49 | 57.56 |
| LYS214 | GLU129 | 56.44 |
| SER184 | SER182 | 56.00 |
| HIE169 | THR170 | 52.67 |
| ALA142 | PHE122 | 51.33 |
| VAL174 | GLN166 | 50.89 |
| GLN39 | TYR93 | 49.78 |
| LEU143 | PHE124 | 49.11 |
| PHE127 | SER127 | 48.44 |
| TYR58 | THR100 | 47.78 |
| TYR94 | GLN44 | 47.56 |
| GLU104 | TRP56 | 47.11 |
| VAL186 | LEU141 | 47.11 |
| LEU105 | TYR55 | 45.78 |
| LYS148 | SER137 | 44.89 |
| GLU104 | TYR97 | 43.11 |
| GLN110 | PRO49 | 42.89 |
| VAL174 | GLU167 | 41.56 |
| TRP47 | THR100 | 40.44 |
| LEU175 | GLN166 | 40.44 |

Table S18: Quantitative overview of the interdomain interactions of the H5-51:L1-39 (4KMT) Fab.

| Heavy | Light | Percent |
| --- | --- | --- |
| LEU106 | TYR36 | 99.17 |
| TRP109 | PRO44 | 94.68 |
| PHE172 | SER176 | 93.07 |
| PHE128 | GLN124 | 92.63 |
| GLN39 | GLN38 | 92.59 |
| LEU45 | PHE98 | 92.34 |
| TYR103 | SER91 | 91.80 |
| TRP47 | LEU96 | 87.12 |
| TYR95 | ALA43 | 83.56 |
| TRP47 | PRO95 | 83.32 |
| GLY110 | ALA43 | 80.00 |
| ALA143 | PHE118 | 79.95 |
| GLU105 | ASN34 | 79.37 |
| TYR95 | GLN38 | 77.37 |
| LYS220 | ASP122 | 77.22 |
| LEU45 | TYR87 | 75.71 |
| ALA143 | PHE116 | 74.78 |
| PHE172 | THR164 | 72.20 |
| PHE172 | LEU175 | 71.71 |
| GLY104 | SER91 | 71.12 |
| LEU130 | PHE118 | 70.49 |
| PHE172 | SER174 | 69.51 |
| PHE172 | SER162 | 69.12 |
| PRO129 | SER121 | 67.02 |
| GLY104 | GLN89 | 66.73 |
| PRO173 | VAL163 | 66.00 |
| ALA131 | PRO119 | 63.76 |
| GLU105 | LEU46 | 62.68 |
| ALA131 | PHE118 | 59.56 |
| GLN39 | TYR87 | 59.51 |
| GLY104 | ASN34 | 58.20 |
| PRO173 | SER162 | 57.76 |
| TYR95 | PRO44 | 57.51 |
| LEU130 | VAL133 | 54.73 |
| TRP109 | TYR36 | 54.59 |
| SER61 | PRO95 | 52.15 |
| PHE128 | GLU123 | 51.51 |
| HIE170 | THR164 | 51.22 |
| LEU144 | PHE118 | 51.02 |
| ALA142 | PHE116 | 50.54 |
| GLU105 | TYR36 | 50.44 |
| TYR99 | LEU96 | 49.12 |
| LYS220 | PRO120 | 48.73 |
| PHE172 | LEU135 | 48.59 |
| VAL187 | LEU135 | 46.98 |
| HIE170 | SER174 | 46.39 |
| THR189 | ASN137 | 45.32 |
| PHE128 | SER121 | 44.63 |
| LYS215 | GLU123 | 44.59 |
| TYR103 | TYR49 | 44.39 |
| GLU105 | TYR49 | 43.51 |
| ASP107 | LEU46 | 43.46 |
| TYR103 | ASN34 | 42.93 |
| LEU147 | SER131 | 42.15 |
| TRP47 | THR94 | 41.37 |
| TYR103 | ALA50 | 40.24 |
| TYR103 | TYR32 | 40.10 |

Table S19: Quantitative overview of the interdomain interactions of the H5-51:L3-11 (5I1J) Fab.

| Heavy | Light | Percent |
| --- | --- | --- |
| CYX222 | CYX214 | 100.00 |
| GLU105 | ARG91 | 97.30 |
| TRP47 | TRP94 | 96.36 |
| LEU45 | PHE98 | 95.10 |
| ARG59 | TRP94 | 94.74 |
| TYR60 | TRP94 | 93.97 |
| GLN39 | GLN38 | 91.88 |
| TRP47 | LEU96 | 90.11 |
| PHE128 | GLN124 | 89.04 |
| LEU106 | TYR36 | 87.85 |
| PHE172 | SER176 | 86.75 |
| TRP109 | PRO44 | 84.80 |
| PRO129 | SER121 | 80.17 |
| ILE50 | TRP94 | 80.06 |
| ALA143 | PHE118 | 79.90 |
| LEU45 | TYR87 | 79.54 |
| SER61 | PRO95 | 77.20 |
| TYR95 | ALA43 | 77.17 |
| PHE172 | SER174 | 72.16 |
| TYR95 | GLN38 | 71.85 |
| ALA131 | PHE118 | 69.93 |
| ALA143 | PHE116 | 68.14 |
| PRO173 | VAL163 | 67.23 |
| TRP109 | TYR36 | 67.03 |
| PHE172 | THR164 | 66.79 |
| PHE172 | SER162 | 65.55 |
| LEU130 | PHE118 | 65.46 |
| ALA131 | PRO119 | 65.08 |
| GLY104 | ARG91 | 64.94 |
| PHE172 | LEU175 | 63.18 |
| LYS220 | ASP122 | 63.15 |
| GLU105 | LEU46 | 61.94 |
| LEU144 | PHE118 | 57.37 |
| GLY110 | ALA43 | 56.93 |
| GLN39 | TYR87 | 56.79 |
| LEU106 | ARG91 | 56.24 |
| PRO173 | SER162 | 54.78 |
| TYR103 | TYR32 | 54.28 |
| LEU130 | VAL133 | 53.48 |
| PHE128 | GLU123 | 52.99 |
| LYS215 | GLU123 | 52.71 |
| PHE172 | LEU135 | 52.08 |
| PHE128 | SER121 | 51.72 |
| LEU106 | GLN89 | 50.29 |
| TRP47 | PRO95 | 48.77 |
| ASP107 | LEU46 | 48.17 |
| GLU105 | TYR49 | 47.67 |
| VAL187 | LEU135 | 47.62 |
| TYR95 | PRO44 | 46.08 |
| TYR103 | ARG91 | 45.03 |
| ALA142 | PHE116 | 44.45 |
| HIE170 | SER174 | 43.27 |
| GLY44 | TYR87 | 41.64 |

Table S20: Quantitative overview of the interdomain interactions of the H5-51:L3-20 (5I1K) Fab.

| Heavy | Light | Percent |
| --- | --- | --- |
| TRP47 | LEU97 | 95.86 |
| LEU106 | TYR37 | 93.48 |
| PHE128 | GLN125 | 91.45 |
| LEU45 | PHE99 | 89.76 |
| GLN39 | GLN39 | 88.62 |
| LEU45 | TYR88 | 86.33 |
| TRP109 | PRO45 | 85.71 |
| GLY104 | TYR92 | 84.63 |
| PHE172 | SER175 | 77.25 |
| PRO173 | SER163 | 74.78 |
| LEU130 | PHE119 | 73.67 |
| PHE172 | SER177 | 71.11 |
| PRO129 | SER122 | 69.36 |
| TYR95 | GLN39 | 67.39 |
| TYR95 | ALA44 | 67.21 |
| LYS220 | ASP123 | 66.68 |
| ALA143 | PHE119 | 66.05 |
| PHE172 | LEU136 | 65.13 |
| PHE172 | LEU176 | 63.43 |
| PRO173 | VAL164 | 61.47 |
| ALA143 | PHE117 | 61.41 |
| ALA131 | PRO120 | 60.51 |
| TYR95 | PRO45 | 56.82 |
| ALA131 | PHE119 | 56.82 |
| SER61 | PRO96 | 56.61 |
| GLN39 | TYR88 | 55.36 |
| GLU105 | LEU47 | 55.30 |
| HIE170 | SER175 | 54.26 |
| GLY110 | ALA44 | 54.14 |
| TRP109 | TYR37 | 53.63 |
| VAL175 | GLN161 | 52.68 |
| VAL187 | LEU136 | 52.11 |
| TYR99 | LEU97 | 52.03 |
| PHE128 | SER122 | 51.79 |
| PHE172 | THR165 | 50.60 |
| GLY44 | TYR88 | 50.27 |
| GLU105 | TYR50 | 49.17 |
| TRP47 | PRO96 | 47.86 |
| TYR103 | TYR92 | 47.62 |
| GLU105 | TYR92 | 46.87 |
| LEU130 | VAL134 | 46.84 |
| PHE128 | GLU124 | 46.63 |
| LEU144 | PHE119 | 45.71 |
| LEU147 | SER132 | 45.09 |
| PHE172 | SER163 | 44.79 |
| HIE170 | ASN138 | 44.40 |
| LYS149 | SER132 | 41.69 |
| PRO173 | THR165 | 40.41 |
| LYS215 | GLU124 | 40.17 |

Table S21: Quantitative overview of the interdomain interactions of the H5-51:L4-1 (5I1L) Fab.

| Heavy | Light | Percent |
| --- | --- | --- |
| CYX222 | CYX220 | 100.00 |
| TRP109 | PRO50 | 97.54 |
| LEU45 | PHE104 | 94.06 |
| PHE128 | GLN130 | 92.52 |
| PHE172 | SER182 | 89.05 |
| TRP47 | LEU102 | 89.02 |
| GLN39 | GLN44 | 87.51 |
| TRP47 | PRO101 | 82.52 |
| LEU106 | TYR42 | 80.71 |
| TYR103 | TYR97 | 79.45 |
| PHE172 | SER180 | 78.71 |
| ALA143 | PHE124 | 76.52 |
| GLY104 | TYR97 | 75.23 |
| PHE172 | LEU181 | 74.98 |
| LEU45 | TYR93 | 74.83 |
| PRO173 | SER168 | 74.37 |
| GLY110 | PRO49 | 73.69 |
| TYR95 | PRO49 | 73.60 |
| TYR95 | GLN44 | 73.51 |
| PRO173 | VAL169 | 71.75 |
| LEU130 | PHE124 | 70.15 |
| PHE172 | THR170 | 67.63 |
| ALA131 | PRO125 | 66.77 |
| PRO129 | SER127 | 66.22 |
| GLN39 | TYR93 | 62.46 |
| ALA143 | PHE122 | 62.25 |
| ALA131 | PHE124 | 61.97 |
| SER61 | PRO101 | 59.78 |
| PHE172 | LEU141 | 59.11 |
| PHE172 | SER168 | 58.03 |
| LYS220 | ASP128 | 56.15 |
| LEU130 | VAL139 | 54.28 |
| TRP109 | TYR42 | 54.25 |
| LEU147 | SER137 | 51.66 |
| LEU144 | PHE124 | 50.74 |
| GLU105 | TYR55 | 49.35 |
| VAL187 | LEU141 | 48.58 |
| LYS135 | GLU219 | 48.25 |
| LYS215 | GLU129 | 47.17 |
| HIE170 | SER180 | 46.80 |
| VAL175 | GLN166 | 46.71 |
| PHE128 | GLU129 | 46.34 |
| TRP47 | THR100 | 45.66 |
| PHE128 | SER127 | 43.94 |
| SER185 | SER182 | 41.08 |
